# To ${d}$, or not to ${d}$: recent developments and comparisons of regularization schemes

**DOI:** 10.1140/epjc/s10052-017-5023-2

**Published:** 2017-07-14

**Authors:** C. Gnendiger, A. Signer, D. Stöckinger, A. Broggio, A. L. Cherchiglia, F. Driencourt-Mangin, A. R. Fazio, B. Hiller, P. Mastrolia, T. Peraro, R. Pittau, G. M. Pruna, G. Rodrigo, M. Sampaio, G. Sborlini, W. J. Torres Bobadilla, F. Tramontano, Y. Ulrich, A. Visconti

**Affiliations:** 1 Paul Scherrer Institut, 5232 Villigen, PSI Switzerland; 2 Physik-Institut, Universität Zürich, 8057 Zürich, Switzerland; 3 Institut für Kern- und Teilchenphysik, TU Dresden, 01062 Dresden, Germany; 4 Physik Department T31, Technische Universität München, 85748 Garching, Germany; 5 Centro de Ciências Naturais e Humanas, UFABC, 09210-170 Santo André, Brazil; 6 Insituto de Física Corpuscular, UVEG–CSIC, Universitat de València, 46980 Paterna, Spain; 7 Departamento de Física, Universidad Nacional de Colombia, Bogotá D.C., Colombia; 8 CFisUC, Department of Physics, University of Coimbra, 3004-516 Coimbra, Portugal; 9 Dipartimento di Fisica ed Astronomia, Università di Padova, 35131 Padua, Italy; 10 INFN, Sezione di Padova, 35131 Padua, Italy; 11 Higgs Centre for Theoretical Physics, The University of Edinburgh, Edinburgh, EH9 3FD UK; 12 Dep. de Física Teórica y del Cosmos and CAFPE, Universidad de Granada, 18071 Granada, Spain; 13 Departamento de Fïsica, ICEX, UFMG, 30161-970 Belo Horizonte, Brazil; 14 Dipartimento di Fisica, Università di Milano, 20133 Milan, Italy; 15 INFN, Sezione di Milano, 20133 Milan, Italy; 16 Dipartimento di Fisica, Università di Napoli, 80126 Naples, Italy; 17 INFN, Sezione di Napoli, 80126 Naples, Italy

## Abstract

We give an introduction to several regularization schemes that deal with ultraviolet and infrared singularities appearing in higher-order computations in quantum field theories. Comparing the computation of simple quantities in the various schemes, we point out similarities and differences between them.

## Introduction

Higher-order calculations in quantum field theories usually involve ultraviolet (UV) and/or infrared (IR) divergences which need to be regularized at intermediate steps. Only after renormalization and proper combination of real and virtual corrections, a finite and regularization-scheme independent result can be obtained. The choice of the regularization scheme matters in several respects of conceptual and practical relevance:

- Mathematical consistency: It must be excluded that the calculational rules lead to internal inconsistencies such as final expressions contradicting each other.
- Unitarity and causality: The final finite result must be compatible with the basic quantum field theoretical properties of unitarity and causality. In practice this compatibility can be shown by proving the equivalence of a given scheme with ${\overline{{{\textsc {ms}}}}}$ or bphz renormalization, which are known to have these properties.
- Symmetries: It is desirable that symmetries like Lorentz invariance, non-Abelian gauge invariance, or supersymmetry are manifestly preserved by the regularization to the largest possible extent. Symmetry breaking by the regularization which does not correspond to anomalies must be compensated by special, symmetry-restoring counterterms.
- Quantum action principle: The regularized quantum action principle is a relation between symmetries of the regularized Lagrangian and Ward/Slavnov–Taylor identities of regularized Green functions. If it is valid in a given regularization scheme, the study of symmetry properties is strongly simplified.
- Computational efficiency: The regularization scheme should allow for efficient calculational techniques and ideally reduce the technical complexity as much as possible.

In recent years, the understanding of traditional regularization schemes has further improved, and novel schemes have been proposed and developed. The motivation for this progress has been to broaden the conceptual basis as well as to enable new efficient, automated analytical and numerical calculational methods. It appears timely to present a uniform and up-to-date description of all schemes and to collect and compare all established properties, definitions, and calculational procedures. This is the goal of the present report. The covered schemes are the following:

- traditional dimensional schemes: conventional dimensional regularization (cdr), the ‘t Hooft–Veltman scheme (hv), the four-dimensional helicity scheme (fdh), and dimensional reduction (dred),
- new, distinctive (re-)formulations of dimensional schemes: the four-dimensional formulation of the fdh scheme (fdf), the six-dimensional formalism (sdf),
- non-dimensional schemes: implicit regularization (ireg), four-dimensional regularization/renormalization (fdr), four-dimensional unsubtraction (fdu).

In the following we present introductions to all these schemes. Having applications and practitioners in mind we will perform some simple calculations to illustrate the differences as well as common features of the schemes. In particular, we aim to sketch the computation of the cross section for $e^+e^-\rightarrow \gamma ^*\rightarrow q\bar{q}$ at next-to-leading order and the fermion self-energy. The quantities are chosen such that potential technical disadvantages of the traditional schemes are exposed and the properties of novel schemes with respect to UV and IR divergences and (sub)renormalization can be illustrated. In a number of footnotes we will directly compare intermediate results and features of the different schemes and comment on their relation.

Of course, much more detailed information is available in the literature and we refer to the references listed in the individual sections for a more in-depth discussion. However, we also have to warn the reader that, unfortunately, the nomenclature and notation used in the literature is far from being unique. This often leads to misunderstandings. In an attempt to avoid these in the future, we have adopted a unified description in this article. As a result, the notation and terms used here will differ in parts from the notation used in the specialized literature referred to. To help further with clearing out some of the misunderstandings and elucidating the relation between the schemes, we will conclude in Sect. 7 by giving a list of concrete statements.

## DS: dimensional schemes CDR, HV, FDH, DRED

### Integration in ${d}$ dimensions and dimensional schemes

Dimensional regularization [1, 2] and variants are the most common regularization schemes for practical calculations in gauge theories of elementary particle physics. In the following we summarize the basic definitions common to all dimensional schemes (ds) discussed in Sects. 2 and 3 and then provide specific definitions for four variants of ds which differ by the rules for the numerator algebra in analytical expressions.

The basic idea of all ds is to regularize divergent integrals by formally changing the dimensionality of space-time and of momentum space. In the present report we always denote the modified space-time dimension by ${d}$, and we set

2.1 $$\begin{aligned} {d}&\equiv 4-2\epsilon . \end{aligned}$$

Correspondingly, a four-dimensional loop integration is replaced by a *d*-dimensional one,^1^

2.2 $$\begin{aligned} \int \frac{{d}^4k_{[4]}}{(2\pi )^4}&\rightarrow {\mu _{\textsc {ds}}^{4-{d}}}\int \frac{{d}^{d}k_{[{d}]}}{(2\pi )^{d}}, \end{aligned}$$

including the scale of dimensional regularization, ${\mu _{\textsc {ds}}}$. After this replacement, UV and IR divergent integrals lead to poles of the form $1/\epsilon ^n$. In Refs. [3, 4], it is shown that such an operation can indeed be defined in a mathematical consistent way and that this operation has the expected properties such as linearity and invariance under shifts of the integration momentum.

To define a complete regularization scheme for realistic quantum field theories, it must be specified how to deal with $\gamma$ matrices, metric tensors, and other objects appearing in analytical expressions. Likewise, it should be specified how to deal with vector fields in the regularized Lagrangian. On a basic level, two decisions need to be made,

- regularize only those parts of diagrams which can lead to divergences, or regularize everything;
- regularize algebraic objects like metric tensors, $\gamma$ matrices, and momenta in ${d}$ dimensions, or in a different dimensionality.

It turns out that there is an elegant way to unify essentially all common variants of ds in a single framework, where all definitions can easily be formulated and where the differences and relations between the schemes become transparent. This framework is based on distinguishing strictly four-dimensional objects, formally ${d}$-dimensional objects, and formally ${d_{s}}$-dimensional objects.^2^ These objects can be mathematically realized [3–5] by introducing a strictly four-dimensional Minkowski space $\text {S}_{[4]}$ and *infinite*-dimensional vector spaces $\text {QS}_{[{d_{s}}]}$, $\text {QS}_{[{d}]}$, $\text {QS}_{[n_\epsilon ]}$, which satisfy the relations

2.3 $$\begin{aligned} \text {QS}_{[{d_{s}}]}=\text {QS}_{[d]}\oplus \text {QS}_{[n_\epsilon ]} , \quad \text {S}_{[4]}\subset \text {QS}_{[d]} . \end{aligned}$$

The space $\text {QS}_{[d]}$ is the natural domain of cdr and of momentum integration in all considered schemes. Using

2.4 $$\begin{aligned} {d}_s \equiv d +n_\epsilon =4-2\epsilon +n_\epsilon , \end{aligned}$$

it is enlarged to $\text {QS}_{[{d_{s}}]}$ via a direct (orthogonal) sum with $\text {QS}_{[n_\epsilon ]}$.^3^

The structure of the vector spaces in Eq. (2.3) gives rise to the following decomposition of metric tensors and $\gamma$ matrices:

2.5 $$\begin{aligned} g_{[{d_{s}}]}^{\mu \nu }=g_{[{d}]}^{\mu \nu }+g_{[n_\epsilon ]}^{\mu \nu } , \quad \gamma _{[{d_{s}}]}^{\mu }=\gamma _{[{d}]}^{\mu }+\gamma _{[n_\epsilon ]}^{\mu } . \end{aligned}$$

Since the quantities in Eq. (2.5) do not have a finite-dimensional representation, in most of the practical calculations only their algebraic properties are relevant,

2.6a $$\begin{aligned} (g_{[\mathrm{dim}]})^{\mu }_{\phantom {\mu }\mu }&= \mathrm{dim} ,\quad (g_{[{d}]} g_{[n_\epsilon ]})^{\mu }_{\phantom {\mu }\nu } = 0 , \end{aligned}$$

2.6b $$\begin{aligned} \{\gamma _{[\mathrm{dim}]}^{\mu }, \gamma _{[\mathrm{dim}]}^{\nu \phantom {\mu }}\}&= 2 g_{[\mathrm{dim}]}^{\mu \nu } ,\quad \{\gamma _{[{d}]}^{\mu }, \gamma _{[n_\epsilon ]}^{\nu \phantom {\mu }}\} = 0 , \end{aligned}$$

with $$\mathrm{dim}\in \{4, {d_{s}}, {d}, n_\epsilon \}$$.

Furthermore, a complete definition of the various dimensional schemes requires one to distinguish two classes of vector fields (VF):^4^

- Vector fields associated with particles in 1PI diagrams or with soft and collinear particles in the initial/final state are in the following called *singular* VF.
- All other vector fields are called *regular* VF.

**Table 1:** Treatment of vector fields in the four different regularization schemes, i.e. prescription which metric tensor has to be used in propagator numerators and polarization sums. The quantity $d_s$ is usually taken to be 4. This table is taken from Ref. [6]

	cdr	hv	fdh	dred
Singular VF	$g_{[{d}]}^{\mu \nu }$	$g_{[{d}]}^{\mu \nu }$	$g_{[{d_{s}}]}^{\mu \nu }$	$g_{[{d_{s}}]}^{\mu \nu }$
Regular VF	$g_{[{d}]}^{\mu \nu }$	$g_{[4]}^{\mu \nu }$	$g_{[4]}^{\mu \nu }$	$g_{[{d_{s}}]}^{\mu \nu }$

**Fig. 1:**
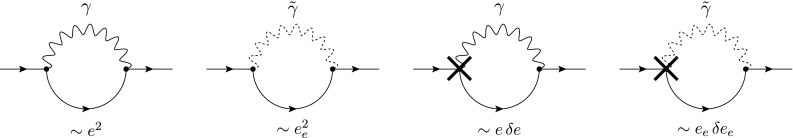
Diagrams contributing to the electron self-energy at the one- and two-loop level including a quasi-${d}$-dimensional photon (*solid wavy line*) and a quasi-$n_\epsilon$-dimensional $\epsilon$-scalar (*dashed wavy line*), respectively. The insertion of a coupling counterterm is denoted by a *cross*. The $\epsilon$-scalar diagrams only exist in fdh and dred

Since UV and IR divergences are only related to *singular* VF there is some freedom in the treatment of the regular ones. In this report, we distinguish the following four ds:

- cdr and hv are two flavours of what is commonly called ‘dimensional regularization’. They regularize algebraic objects in ${d}$ dimensions, $n_\epsilon$-dimensional objects are not used. In cdr, all VF are regularized, in hv only singular ones.
- fdh and dred are two flavours of what is commonly called ‘dimensional reduction’. They regularize algebraic objects in ${d_{s}}$ dimensions. Sometimes ${d_{s}}$ is identified as ${d_{s}}\equiv 4$ from the beginning, but it is possible to keep it as a free parameter, which is set to 4 only at the end of a calculation. In dred, all VF are regularized, in fdh only singular ones.

The definitions of these four schemes can be essentially reduced to the treatment of vector fields; see Table 1. This unified formulation of the four schemes makes obvious that a calculation in dred covers all elements of a calculation in the other schemes.

In fdh and dred, where singular vector fields are treated in ${d_{s}}$ dimensions, the split of Eq. (2.5) can be applied to the regularized Lagrangian and to covariant derivatives. As an illustration, we provide here the regularized covariant derivatives in QED and QCD,

2.7a $$\begin{aligned} \text {QED:}\quad D_{[{d_{s}}]}^{\mu } \psi _i&=\partial _{[d]}^{\mu } \psi _i +i (e A_{[{d}]}^{\mu }+e_e A_{[n_\epsilon ]}^{\mu } ) Q \psi _i , \nonumber \\\end{aligned}$$

2.7b $$\begin{aligned} \text {QCD:}\quad D_{[{d_{s}}]}^{\mu } \psi _i&=\partial _{[d]}^{\mu } \psi _i +i (g_s A_{[{d}]}^{\mu ,a}+g_e A_{[n_\epsilon ]}^{\mu ,a} ) T^a_{ij} \psi _j . \end{aligned}$$

It is important that the gauge-field part is *not* written as a complete ${d_{s}}$-dimensional entity but is split into ${d}$-dimensional and $n_\epsilon$-dimensional parts, and particularly with independent couplings. Conventionally, the $n_\epsilon$-dimensional fields are called ‘$\epsilon$-scalars’, the associated couplings are called ‘evanescent couplings’. This split is strictly necessary at the multi-loop level in non-supersymmetric theories since the evanescent couplings are not protected by ${d}$-dimensional Lorentz and gauge invariance and renormalize differently compared to the corresponding gauge couplings. As an example, we provide the (minimal) renormalization of the QED gauge coupling and the corresponding evanescent coupling in fdh/dred,

2.8a $$\begin{aligned} \beta _{\phantom {e}}&=\mu ^2\frac{\text {d}}{\text {d}\mu ^2}\left( \frac{e}{4\pi }\right) ^2 =-\left( \frac{e}{4\pi }\right) ^4 \left[ - \frac{4}{3}N_F\right] +\cdots , \end{aligned}$$

2.8b $$\begin{aligned} \beta _e&=\mu ^2\frac{\text {d}}{\text {d}\mu ^2}\left( \frac{e_e}{4\pi }\right) ^2 =-\left( \frac{e_e}{4\pi }\right) ^4 [ - 4 - 2 N_F]\nonumber \\&\quad -\left( \frac{e}{4\pi }\right) ^2\left( \frac{e_e}{4\pi }\right) ^2 [ + 6 ] +\cdots . \end{aligned}$$

These values can be obtained e.g. from Ref. [7] by setting $C_A\rightarrow 0$, $N_F\rightarrow 2 N_F$. It is obvious that even for $e_e=e$, the values of $\beta$ and $\beta _e$ are not the same.

### Application example 1: electron self-energy at NLO

To illustrate the different treatment of the Lorentz algebra in the various ds, we consider the electron self-energy at NLO in dred; see Fig. 1. As mentioned in the previous section, this can be seen as the most comprehensive case of the four considered ds. For simplicity, we use massless QED as underlying theory. On the one hand, the Lorentz algebra can then be evaluated by applying the split of Eq. (2.5),

2.9 $$\begin{aligned} -i \Sigma _{{{\textsc {dred}}}}^{(1)}&= -i \{\Sigma ^{(1)}(e^2)+\tilde{\Sigma }^{(1)}(e_e^2) \} \nonumber \\&={\mu _{\textsc {ds}}^{4-{d}}}\int \frac{{d}^{{d}}k_{[{d}]}}{(2\pi )^{{d}}} \{ e^2 \gamma ^{\mu \phantom {\mu }}_{[{d}]} \gamma ^{\nu \phantom {\mu }}_{[{d}]} \gamma ^{\rho \phantom {\mu }}_{[{d}]} (g_{[{d}]}^{\phantom {\mu }})_{\mu \rho }\nonumber \\&\quad + e_e^2 \gamma ^{\mu \phantom {\mu }}_{[n_\epsilon ]} \gamma ^{\nu \phantom {\mu }}_{[{d}]} \gamma ^{\rho \phantom {\mu }}_{[n_\epsilon ]} (g_{[n_\epsilon ]}^{\phantom {\mu }})_{\mu \rho } \} \frac{(k_{[{d}]}^{\phantom {\mu }})_{\nu } }{[k_{[{d}]}^{2}] [(k_{[{d}]}^{\phantom {2}} +p_{[{d}]}^{\phantom {2}})^{2}]} \nonumber \\&= {\mu _{\textsc {ds}}^{4-{d}}}\int \frac{{d}^{{d}}k_{[{d}]}}{(2\pi )^{{d}}} \{ e^2 ( - \gamma ^{\mu \phantom {\mu }}_{[{d}]} (\gamma _{[{d}]}^{\phantom {\mu }})_{\mu } \gamma ^{\nu \phantom {\mu }}_{[{d}]} + 2 \gamma ^{\nu \phantom {\mu }}_{[{d}]} )\nonumber \\&\quad + e_e^2 ( - \gamma ^{\mu \phantom {\mu }}_{[n_\epsilon ]} (\gamma _{[n_\epsilon ]}^{\phantom {\mu }})_{\mu } \gamma ^{\nu \phantom {\mu }}_{[{d}]} ) \} \frac{(k_{[{d}]}^{\phantom {\mu }})_{\nu }}{[ \dots ] [ \dots ]} \nonumber \\&= {\mu _{\textsc {ds}}^{4-{d}}}\int \frac{{d}^{{d}}k_{[{d}]}}{(2\pi )^{{d}}} \{ e^2 ( - {d}+ 2 ) +e_e^2 ({d}- {d_{s}}) \} \nonumber \\&\qquad \times \frac{\gamma ^{\nu }_{[{d}]} (k_{[{d}]}^{\phantom {\mu }})_{\nu }}{[ \dots ] [ \dots ]} , \end{aligned}$$

where Feynman gauge and the equality $n_\epsilon = (d_s - {d})$ have been used. Setting $n_\epsilon = 0$ then corresponds to the results in cdr and hv.

On the other hand, for $e_e=e$, the amplitude can also be evaluated more directly by using a quasi-${d_{s}}$-dimensional algebra,

2.10 $$\begin{aligned} -i \Sigma _{{{\textsc {dred}}}}^{(1)}&= {\mu _{\textsc {ds}}^{4-{d}}}\int \frac{{d}^{{d}}k_{[{d}]}}{(2\pi )^{{d}}} \{ e^2 \gamma ^{\mu \phantom {\mu }}_{[{d_{s}}]} \gamma ^{\nu \phantom {\mu }}_{[{d}]} \gamma ^{\rho \phantom {\mu }}_{[{d_{s}}]} (g_{[{d_{s}}]}^{\phantom {\mu }})_{\mu \rho } \} \nonumber \\&\quad \times \frac{(k_{[{d}]}^{\phantom {\mu }})_{\nu }}{[k_{[{d}]}^{2}] [(k_{[{d}]}^{\phantom {2}} +p_{[{d}]}^{\phantom {2}})^{2}]} \nonumber \\&= {\mu _{\textsc {ds}}^{4-{d}}}\int \frac{{d}^{{d}}k_{[{d}]}}{(2\pi )^{{d}}} \{ e^2 ( - \gamma ^{\mu \phantom {\mu }}_{[{d_{s}}]} (\gamma _{[{d_{s}}]}^{\phantom {\mu }})_{\mu } \gamma ^{\nu \phantom {\mu }}_{[{d_{s}}]} + 2 \gamma ^{\nu \phantom {\mu }}_{[{d_{s}}]}) \}\nonumber \\&\quad \times \frac{(k_{[{d}]}^{\phantom {\mu }})_{\nu }}{[ \dots ] [ \dots ]} \nonumber \\&= {\mu _{\textsc {ds}}^{4-{d}}}\int \frac{{d}^{{d}}k_{[{d}]}}{(2\pi )^{{d}}} \{ e^2 (- {d_{s}}+ 2)\} \frac{\gamma ^{\nu }_{[{d}]} (k_{[{d}]}^{\phantom {\mu }})_{\nu }}{[ \dots ] [ \dots ]} . \end{aligned}$$

In the second line, the identity $\gamma ^{\nu }_{[{d}]} (k_{[{d}]}^{\phantom {\nu }})_{\nu } =\gamma ^{\nu }_{[{d_{s}}]} (k_{[{d}]}^{\phantom {\nu }})_{\nu }$ is used which directly follows from the structure of the vector spaces in Eq. (2.3).

When setting $d_s =4$, one obtains the result in fdh/dred. Moreover, setting $e_e =e$ with $\alpha =e^2/(4\pi )$, it follows that the different treatment of the algebra in Eqs. (2.9) and (2.10) yields the same result,2.11As long as no distinction between gauge and evanescent couplings is required, both approaches are therefore equivalent.

At the two-loop level, however, the different UV renormalization of *e* and $e_e$ enters via the counterterm diagrams shown on the right of Fig. 1,

2.12 $$\begin{aligned} -i \Sigma ^{(2,{{\textsc {ct}}})}_{{{\textsc {dred}}}} =-i \{ \delta ^{(1)} e^2 \times \Sigma ^{(1)}(e^2) +\delta ^{(1)} e_e^2 \times \tilde{\Sigma }^{(1)}(e_e^2) \} . \end{aligned}$$

Since no distinction between the couplings is possible when using a quasi-${d_{s}}$-dimensional algebra, in this case it is mandatory to apply the split of Eq. (2.5). Generalizing to an arbitrary $\ell$-loop calculation, the introduction and separate treatment of $\epsilon$-scalars has to be considered up to $(\ell -1)$ loops. Genuine $\ell$-loop diagrams, on the other hand, can either be evaluated by using the split of Eq. (2.5) or by using a quasi-${d_{s}}$-dimensional Lorentz algebra. Further details regarding the UV renormalization in the various ds can be found in Refs. [7–11].

### Application example 2: $e^{+} e^{-}\rightarrow \gamma ^{*}\rightarrow q\bar{q}$ at NLO

Any physical observable has to be independent of the regularization scheme. What is usually done in computing NLO cross sections is to obtain the virtual corrections in cdr (either directly, or first in another scheme and then translated to cdr) and combine them with the real corrections calculated in cdr. As shown in Ref. [6], it is also possible to compute the real corrections directly in schemes other than cdr.

We use the very simple process $e^{+}e^{-} \rightarrow \gamma ^{*} \rightarrow q\bar{q}$ with massless quarks to illustrate the interplay between the scheme dependence in the real and virtual corrections at NLO in QCD. To simplify further, we average over the directions of the incoming leptons (with momenta *p* and $p'$) and actually consider only $\gamma ^{*} \rightarrow q \bar{q}$. This is achieved by replacing the (regularization-scheme dependent) leptonic tensor by2.13where $s\equiv q^2=(p+p')^2$. In the first step, the average is taken in $\mathrm{dim}$ dimensions. However, the prefactor will be an overall factor of the full cross section. Hence, for this prefactor we set $\mathrm{dim}=4$ from the beginning and the only scheme dependence that is left in $L_{{{\textsc {ds}}}}^{\mu \nu }$ is in the one in $g^{\mu \nu }_{[\mathrm{dim}]}$. The following discussion might create the impression that schemes other than cdr are complicated to use. However, this is simply because we will give the details of the field-theoretic background. This results in many apparent ‘complications’ that can actually be avoided at a practical level.

**Fig. 2:**
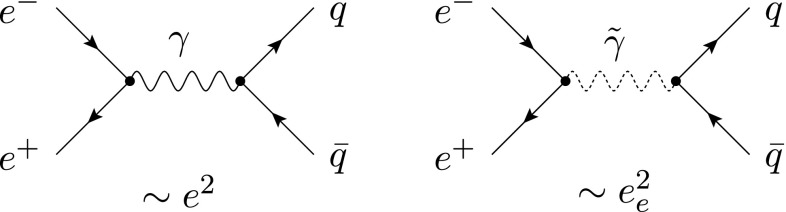
Tree-level diagrams contributing to the process $e^{+}e^{-} \rightarrow \gamma ^{*} \rightarrow q\bar{q}$. The interaction is mediated by a photon $\gamma$ (*left*) and an $\epsilon$-scalar photon $\tilde{\gamma }$ (*right*), respectively. The *left diagram* is present in all considered schemes, whereas the *right* one only exists in dred

**Fig. 3:**
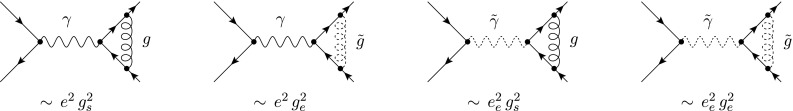
Virtual diagrams for $e^{+}e^{-} \rightarrow \gamma ^{*} \rightarrow q\bar{q}$ including a gluon *g* or an $\epsilon$-scalar $\tilde{g}$. In cdr and hv, only the *first diagram* contributes, whereas in fdh also the *second diagram* is present. In dred, all diagrams contribute

Let us begin with the most straightforward case of cdr, where the regular photon is treated in ${d}$ dimensions. Here, only the left diagram in Fig. 2 contributes. According to Table 1, the metric tensor of the photon propagator – and hence in Eq. (2.13) – is $g^{\mu \nu }_{[{d}]}$, the coupling at the vertices is the gauge coupling *e*. Using Eq. (2.13), we get for the (spin summed/averaged) squared matrix element $M_{{{\textsc {ds}}}}^{(0)}=\langle \mathcal {A}_{{{\textsc {ds}}}}^{(0)} | \mathcal {A}_{{{\textsc {ds}}}}^{(0)}\rangle$

2.14a $$\begin{aligned} M_{{\textsc {cdr}}}^{(0)} =\frac{{Q_{q}^{2}}N_c}{3 s} e^4 (d-2) \equiv \omega ^{(0)} e^4 (d-2) , \end{aligned}$$

where ${Q_{q}}=-1/3, 2/3$ and $N_c$ are the electric charge and the colour number of the quark, respectively, and the flux factor 1 / (2*s*) is included.

In hv and fdh, the regular photon is kept unregularized; the related metric tensor is therefore $g^{\mu \nu }_{[4]}$. The squared amplitudes are then given by

2.14b $$\begin{aligned} M_{{\textsc {hv}}}^{(0)} =M_{\textsc {fdh}}^{(0)} =\omega ^{(0)} e^4 (4-2) . \end{aligned}$$

In contrast to this, in dred, the regular photon is treated in ${d}_s$ dimensions and thus contains a gauge-field part and an $\epsilon$-scalar part. It is therefore possible to decompose the Born amplitude into the two diagrams of Fig. 2. The crucial point is that the diagrams involve different couplings; the left diagram is proportional to the square of the electric gauge coupling *e* as in the other schemes, whereas the right diagram is proportional to $e_e^2$. The result of the squared matrix element in dred therefore reads

2.14c $$\begin{aligned} M_{\textsc {dred}}^{(0)}&= M_{\textsc {dred}}^{(0,\gamma )} + M_{\textsc {dred}}^{(0,\tilde{\gamma })} = M_{\textsc {cdr}}^{(0)} + M_{\textsc {dred}}^{(0,\tilde{\gamma })}\nonumber \\&= \omega ^{(0)} [ e^4 (d-2) + e^4_{e} (n_\epsilon ) ] . \end{aligned}$$

The appearance of a second contributions in dred is one of those apparent complications mentioned above. In practice, one usually sets $e_e=e$ from the beginning and computes the two processes in a combined way like in Eq. (2.10). This is possible since the different UV renormalizations of *e* and $e_e$ are irrelevant in this case.

Using the results in Eq. (2.14) and integrating over the phase space, we obtain the (scheme-independent) Born cross section

2.15 $$\begin{aligned} \sigma ^{(0)}&=\frac{\Phi _2(\epsilon )}{8\pi } M_{{{\textsc {ds}}}}^{(0)} \bigg |_{d\rightarrow 4} =\frac{{Q_{q}^{2}}N_c}{3 s}\left( \frac{e^4}{4\pi }\right) , \end{aligned}$$

where we separate the ${d}$-dependent two-body phase space

2.16 $$\begin{aligned} \Phi _2(\epsilon ) =\left( \frac{4\pi }{s}\right) ^\epsilon \frac{\Gamma (1-\epsilon )}{\Gamma (2-2\epsilon )} =1+\mathcal{O}(\epsilon ) . \end{aligned}$$

#### Virtual contributions

In a next step we consider the virtual corrections to the (spin summed/averaged) squared matrix element, $M_{{{\textsc {ds}}}}^{(1)}=2{\text {Re}} \langle \mathcal {A}_{{{\textsc {ds}}}}^{(0)} | \mathcal {A}_{{{\textsc {ds}}}}^{(1)}\rangle$. To obtain the results of the corresponding one-loop amplitudes, we have to evaluate the diagrams shown in Fig. 3. There are two different vector fields in the one-loop diagrams, a virtual photon that is ‘regular’ and a virtual gluon that is ‘singular’. According to this, the treatment of the photon is as for the Born amplitude. For dred, this results in two contributions, one proportional to the gauge coupling *e*, the other proportional to the evanescent coupling $e_e$. Due to the Ward identity, only the latter coupling gets renormalized. In the ${\overline{{{\textsc {ms}}}}}$ scheme, we obtain

2.17 $$\begin{aligned} ({Q_{q}}e_{e})^2&\rightarrow ({Q_{q}}e_{e})^2 \left\{ 1 + \bigg (\frac{\alpha _s}{4\pi }\bigg ) {C_F}\left[ - \frac{3}{\epsilon }\right] \right. \nonumber \\&\quad \left. +\left( \frac{\alpha _e}{4\pi }\right) {C_F}\frac{4-n_\epsilon }{2 \epsilon }\right\} . \end{aligned}$$

We remark that in schemes other than cdr, the ${\overline{{{\textsc {ms}}}}}$ counterterms in general can have $\mathcal {O}(n_\epsilon )$ terms, as discussed e.g. in Ref. [12]. In dred, one therefore has to consider the (finite) counterterm

2.18 $$\begin{aligned} \mathrm{CT}_{\textsc {dred}}= M_{\textsc {dred}}^{(0,\tilde{\gamma })} {C_F}\bigg \{\bigg (\frac{\alpha _s}{4\pi }\bigg ) \bigg [ -\frac{6}{\epsilon }\bigg ] +\bigg (\frac{\alpha _e}{4\pi }\bigg ) \frac{4-n_\epsilon }{\epsilon }\bigg \} ; \end{aligned}$$

**Fig. 4:**
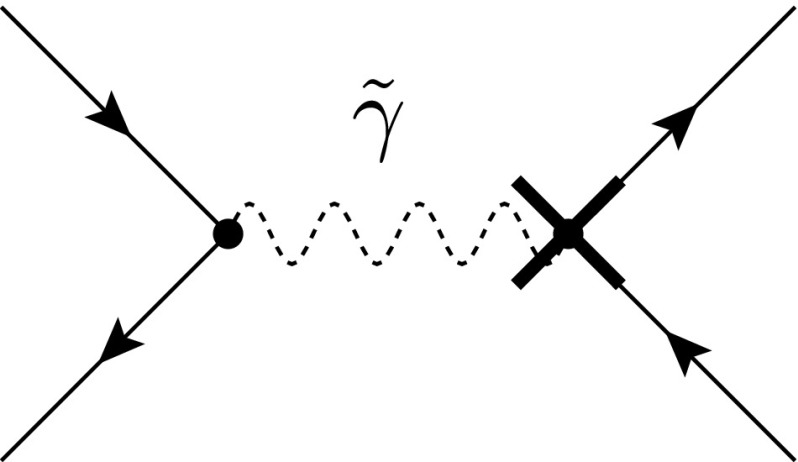
Counterterm diagram for $e^{+}e^{-} \rightarrow \gamma ^{*} \rightarrow q\bar{q}$ which only contributes in dred

**Fig. 5:**
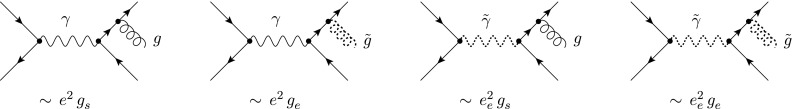
Real diagrams for $e^+ e^- \rightarrow q\bar{q} g$ and $e^+ e^- \rightarrow q\bar{q} \tilde{g}$. In cdr and hv there is only the first diagram, whereas in fdh also the second diagram is present. In dred, all diagrams contribute. An analogous diagram where the gluon couples to the other quark leg is understood

see also Fig. 4. In the same way, when using fdh or dred, the gluon can be split according to Eq. (2.5). Thus, in these schemes we get terms proportional to $\alpha _s =g_s^2/(4\pi )$ and terms proportional to $\alpha _e =g_e^2/(4\pi )$. The unrenormalized virtual one-loop corrections are given by

2.19a $$\begin{aligned} M_{{\textsc {cdr}}\phantom {J}}^{(1)}&=\ \omega ^{(1)} M_{\textsc {cdr}}^{(0)} \big (\frac{\alpha _s}{\pi }\bigg ) \bigg [ -\frac{1}{\epsilon ^2}-\frac{3}{2 \epsilon }-4\bigg ] + \mathcal {O}(\epsilon ) ,\phantom {\bigg \Vert }\end{aligned}$$

2.19b $$\begin{aligned} M_{{\textsc {hv}}\phantom {JJ}}^{(1)}&=\ \omega ^{(1)} M_{\textsc {hv}}^{(0)} \bigg (\frac{\alpha _s}{\pi }\bigg ) \bigg [ -\frac{1}{\epsilon ^2}-\frac{3}{2 \epsilon }-4\bigg ] + \mathcal {O}(\epsilon ) ,\phantom {\bigg \Vert } \end{aligned}$$

2.19c $$\begin{aligned} M_{{\textsc {fdh}}\phantom {J}}^{(1)}&= \omega ^{(1)} M_{\textsc {fdh}}^{(0)} \bigg \{\bigg (\frac{\alpha _s}{\pi }\bigg ) \bigg [ -\frac{1}{\epsilon ^2}-\frac{3}{2 \epsilon }-4\bigg ]\nonumber \\&\quad +\bigg (\frac{\alpha _e}{\pi }\bigg ) \bigg [\frac{n_\epsilon }{4 \epsilon }\bigg ] \bigg \} +\mathcal {O}(\epsilon ) , \end{aligned}$$

2.19d $$\begin{aligned} M_{\textsc {dred}}^{(1)}&=\ \omega ^{(1)} M_{\textsc {dred}}^{(0,\gamma )} \bigg \{\bigg (\frac{\alpha _s}{\pi }\bigg ) \bigg [ -\frac{1}{\epsilon ^2}-\frac{3}{2 \epsilon }-4\bigg ]\nonumber \\&\quad +\bigg (\frac{\alpha _e}{\pi }\bigg ) \bigg [\frac{n_\epsilon }{4 \epsilon }\bigg ] \bigg \} \nonumber \\&\quad + \omega ^{(1)} M_{\textsc {dred}}^{(0,\tilde{\gamma })} \bigg \{\bigg (\frac{\alpha _s}{\pi }\bigg ) \bigg [ -\frac{1}{\epsilon ^2}\bigg ] + \bigg (\frac{\alpha _e}{\pi }\bigg ) \bigg [ -\frac{1}{\epsilon }\bigg ] \bigg \}\nonumber \\&\quad + \mathcal {O}(\epsilon ) , \end{aligned}$$

with

2.20a $$\begin{aligned} \omega ^{(1)}&\equiv {C_F}c_\Gamma (\epsilon ) \mathrm{Re} (-s)^{-\epsilon } = {C_F}c_\Gamma (\epsilon ) s^{-\epsilon } \nonumber \\&\quad \times \bigg [1-\epsilon ^2\frac{\pi ^2}{2}+\mathcal {O}(\epsilon ^4)\bigg ] ,\end{aligned}$$

2.20b $$\begin{aligned} c_\Gamma (\epsilon )&=(4\pi )^{\epsilon } \frac{\Gamma (1+\epsilon ) \Gamma ^2(1-\epsilon )}{\Gamma (1-2\epsilon )} =1+\mathcal {O}(\epsilon ) . \end{aligned}$$

In Eq. (2.19), we have dropped $n_\epsilon$ terms that vanish after setting $n_\epsilon = 2\epsilon$ and taking the subsequent limit $\epsilon \rightarrow 0$.

In particular, the dred result looks awfully complicated. However, from a practical point of view the situation is much simpler. As discussed in the previous section, the virtual contributions can be computed without distinguishing the various couplings and without splitting the photon or the gluon. We can simply evaluate the algebra of the single vertex diagram according to the scheme and perform the integration. The only part where the split is crucial so far is to obtain the UV counterterm, Eq. (2.18). Thus, the computation in schemes other than cdr is not significantly more extensive.

Computing the (IR divergent) virtual cross section by integrating the properly(!) renormalized matrix element squared over the two-parton phase space, Eq. (2.16), we get

2.21a $$\begin{aligned} \sigma ^{(v)}_{\textsc {cdr}}&= \sigma ^{(0)} \bigg (\frac{\alpha _s}{\pi }\bigg ) {C_F}\Phi _2(\epsilon ) c_\Gamma (\epsilon ) s^{-\epsilon }\nonumber \\&\quad \times \bigg [ -\frac{1}{\epsilon ^2} -\frac{1}{2 \epsilon } -\frac{5-\pi ^2}{2} + \mathcal {O}(\epsilon ) \bigg ] , \phantom {\bigg |} \end{aligned}$$

2.21b $$\begin{aligned} \sigma ^{(v)}_{{\textsc {hv}}\phantom {J}}&= \sigma ^{(0)} \bigg (\frac{\alpha _s}{\pi }\bigg ) {C_F}\Phi _2(\epsilon ) c_\Gamma (\epsilon ) s^{-\epsilon }\nonumber \\&\quad \times \bigg [ -\frac{1}{\epsilon ^2} -\frac{3}{2 \epsilon } -\frac{8-\pi ^2}{2} + \mathcal {O}(\epsilon ) \bigg ] , \phantom {\bigg |} \end{aligned}$$

2.21c $$\begin{aligned} \sigma ^{(v)}_{\textsc {fdh}}=\ \sigma ^{(v)}_{\textsc {dred}}&= \sigma ^{(0)} \bigg (\frac{\alpha _s}{\pi }\bigg ) {C_F}\Phi _2(\epsilon ) c_\Gamma (\epsilon ) s^{-\epsilon }\nonumber \\&\quad \times \bigg [ -\frac{1}{\epsilon ^2} -\frac{3}{2 \epsilon } -\frac{7-\pi ^2}{2} + \mathcal {O}(\epsilon )\bigg ] , \phantom {\bigg |} \end{aligned}$$

where we have set $n_\epsilon =2\epsilon$ and $g_e=g_s$.

#### Real contributions

Finally we have to face the real corrections. In cdr, the amplitude consists of two diagrams (one of which is depicted in Fig. 5). The matrix element squared, expressed in terms of $s_{ij} \equiv 2 p_i\cdot p_j$ reads

2.22a $$\begin{aligned}&M^{(0)}_{\textsc {cdr}}(q\bar{q} g) \nonumber \\&\quad = \omega ^{(r)} e^4 g_s^2 (d-2)\nonumber \\&\qquad \times \bigg \{ \bigg [\frac{(s_{12}+s_{13})^2}{s_{13} s_{23}} + \frac{d-4}{2} \frac{s_{13}+s_{23}}{s_{23}}\bigg ] + [1\leftrightarrow 2 ] \bigg \} , \end{aligned}$$

where $\omega ^{(r)} =\omega ^{(0)} 2 {C_F}/s$. In hv, the same diagrams contribute. One might be tempted to assume that $M^{(0)}_{\textsc {hv}}(q\bar{q} g)$ can be obtained from Eq. (2.22a) simply by setting $d\rightarrow 4$. However, this is incorrect. In the regime where the gluons become collinear, they have to be treated as singular gluons. Thus, in hv they are *d*-dimensional. The same is true in principle for the soft region, but at one loop, there is no scheme dependence in the soft singularities. This corresponds to the statement that the cusp anomalous dimension is scheme independent at the one-loop level [13, 14]. Treating the gluons properly, we obtain

2.22b $$\begin{aligned} M^{(0)}_{\textsc {hv}}(q\bar{q} g)&= \frac{2}{d-2} M^{(0)}_{\textsc {cdr}}(q\bar{q} g) . \end{aligned}$$

In the case of fdh we get contributions ${\sim }g_s$ and ${\sim }g_e$. Again, the gluon has to be treated as a singular one. Hence, it is split into a *d*-dimensional gluon and an $\epsilon$-scalar, resulting in

2.22c $$\begin{aligned} M^{(0)}_{\textsc {fdh}}(q\bar{q} g) + M^{(0)}_{\textsc {fdh}}(q\bar{q} \tilde{g})&= M^{(0)}_{\textsc {hv}}(q\bar{q} g)\nonumber \\&\quad + \omega ^{(r)} e^4 g_e^2 n_\epsilon \frac{(s_{13} + s_{23})^2}{s_{13} s_{23}} . \end{aligned}$$

Finally, as illustrated in Fig. 5, in dred the matrix element squared is formally decomposed into four parts,

2.22d $$\begin{aligned} M^{(0)}_{\textsc {dred}}(q\bar{q} g)&= M^{(0, \gamma )}_{\textsc {dred}}(q\bar{q} g) + M^{(0,\gamma )}_{\textsc {dred}}(q\bar{q}\tilde{g})\nonumber \\&\quad + M^{(0, \tilde{\gamma })}_{\textsc {dred}}(q\bar{q} g) + M^{(0,\tilde{\gamma })}_{\textsc {dred}}(q\bar{q}\tilde{g}) \phantom {\bigg |} \nonumber \\&= M^{(0)}_{\textsc {cdr}}(q\bar{q} g) + e_e^4 g_s^2 n_\epsilon \nonumber \\&\quad \times \frac{4 s s_{12} + (2-n_\epsilon ) (s_{13} + s_{23})^2}{2 s_{13} s_{23}} \nonumber \\&\quad +\frac{d-2}{2} M^{(0)}_{\textsc {fdh}}(q\bar{q} \tilde{g}) + e_e^4 g_e^2 n_\epsilon \nonumber \\&\quad \times \frac{-4 s_{13} s_{23} + n_\epsilon (s_{13} + s_{23})^2}{2 s_{13} s_{23}} . \end{aligned}$$

Note that if we set $e_e =e$ and $g_e =g_s$, the matrix element in dred corresponds to the usual four-dimensional matrix element,

2.23 $$\begin{aligned}&M^{(0)}_{\textsc {dred}}(q\bar{q} g) \bigg |_{\begin{array}{c} e_e=e_{\phantom {s}}\\ g_e=g_s \end{array}} = M^{(0)}_{\textsc {cdr}}(q\bar{q} g) \bigg |_{d=4} \nonumber \\&\quad = \omega ^{(r)} e^4 g_{s}^2 4 \bigg ( -\frac{1}{y_{13}} -\frac{1}{y_{23}} +\frac{y_{13}}{2 y_{23}} +\frac{y_{23}}{2 y_{13}} +\frac{1}{y_{13} y_{23}} \bigg ) ,\nonumber \\ \end{aligned}$$

with $y_{ij} \equiv s_{ij}/s$. This is generally true for arbitrary tree-level amplitudes in dred, but not necessarily in any of the other schemes. For the considered process, it happens to be true also in fdh.

The real cross section can now be obtained in any scheme by integrating the corresponding matrix element over the *d*-dimensional real phase space,

2.24a $$\begin{aligned} \sigma ^{(r)}_{{{\textsc {ds}}}}&= \frac{s}{2 (4\pi )^3} \Phi _3(\epsilon ) \int _0^1 {d}y_{13}\nonumber \\&\quad \times \int _{0}^{1-y_{13}} {d}y_{23}\ y_{13}^{-\epsilon }\ y_{23}^{-\epsilon }\ (1-y_{13}-y_{23})^{-\epsilon } M^{(0)}_{{{\textsc {ds}}}}(q\bar{q} g) \end{aligned}$$

2.24b $$\begin{aligned}&\equiv \frac{s}{2 (4\pi )^3} \Phi _3(\epsilon ) \iint \limits _{y_{13} y_{23}} y_{13}^{-\epsilon }\ y_{23}^{-\epsilon }\ (1-y_{13}-y_{23})^{-\epsilon } M^{(0)}_{{{\textsc {ds}}}}(q\bar{q} g) . \end{aligned}$$

Similar to the two-particle phase space, we extract a ${d}$-dependent factor

2.25 $$\begin{aligned} \Phi _3(\epsilon ) =\bigg (\frac{4\pi }{s}\bigg )^{2\epsilon }\frac{1}{\Gamma (2-2\epsilon )} = 1 + \mathcal{O}(\epsilon ) . \end{aligned}$$

For future reference, we explicitly list the integrals needed to evaluate Eq. (2.24),

2.26a $$\begin{aligned} \iint \limits _{y_{13} y_{23}} y_{13}^{-\epsilon } y_{23}^{-\epsilon } (1-y_{13}-y_{23})^{-\epsilon } \frac{1}{y_{13}}&= -\frac{1}{\epsilon } - 3 +\mathcal {O}(\epsilon ) , \end{aligned}$$

2.26b $$\begin{aligned} \iint \limits _{y_{13} y_{23}} y_{13}^{-\epsilon } y_{23}^{-\epsilon } (1-y_{13}-y_{23})^{-\epsilon } \frac{y_{23}}{y_{13}}&= -\frac{1}{2 \epsilon } - \frac{7}{4} +\mathcal {O}(\epsilon ) , \end{aligned}$$

2.26c $$\begin{aligned} \iint \limits _{y_{13} y_{23}} y_{13}^{-\epsilon } y_{23}^{-\epsilon } (1-y_{13}-y_{23})^{-\epsilon } \frac{1}{y_{13} y_{23}}&= \frac{1}{\epsilon ^2} - \frac{\pi ^2}{2} +\mathcal {O}(\epsilon ) . \end{aligned}$$

Using these results for the calculation of the real corrections in the various schemes and setting $e_e = e$, $g_e=g_s$, $n_\epsilon =2\epsilon$, we obtain

2.27a $$\begin{aligned} \sigma ^{(r)}_{\textsc {cdr}}&= \sigma ^{(0)} \bigg (\frac{\alpha _s}{\pi }\bigg ) {C_F}\Phi _3(\epsilon ) \nonumber \\&\quad \times \bigg [ \frac{1}{\epsilon ^2} +\frac{1}{2 \epsilon } +\frac{13}{4}-\frac{\pi ^2}{2} + \mathcal {O}(\epsilon ) \bigg ] ,\phantom {\bigg |} \end{aligned}$$

2.27b $$\begin{aligned} \sigma ^{(r)}_{{\textsc {hv}}\phantom {J}}&= \sigma ^{(0)} \bigg (\frac{\alpha _s}{\pi }\bigg ) {C_F}\Phi _3(\epsilon ) \nonumber \\&\quad \times \bigg [ \frac{1}{\epsilon ^2} +\frac{3}{2 \epsilon } +\frac{19}{4}-\frac{\pi ^2}{2} + \mathcal {O}(\epsilon ) \bigg ] ,\phantom {\bigg |} \end{aligned}$$

2.27c $$\begin{aligned} \sigma ^{(r)}_{\textsc {fdh}}= \sigma ^{(r)}_{\textsc {dred}}&= \sigma ^{(0)} \bigg (\frac{\alpha _s}{\pi }\bigg ) {C_F}\Phi _3(\epsilon ) \nonumber \\&\quad \times \bigg [ \frac{1}{\epsilon ^2} +\frac{3}{2 \epsilon } +\frac{17}{4}-\frac{\pi ^2}{2} + \mathcal {O}(\epsilon ) \bigg ] . \phantom {\bigg |} \end{aligned}$$

And, at long last, we find the well-known regularization-scheme independent physical cross section

2.28 $$\begin{aligned} \sigma ^{(1)}&= \sigma ^{(0)} + \sigma ^{(v)}_{{{\textsc {ds}}}} + \sigma ^{(r)}_{{{\textsc {ds}}}}\bigg |_{d\rightarrow 4} = \frac{{Q_{q}^{2}}N_c}{3 s}\bigg (\frac{e^4}{4\pi }\bigg )\nonumber \\&\quad \times \bigg [ 1+\bigg (\frac{\alpha _s}{4\pi }\bigg ) 3 {C_F}\bigg ] . \end{aligned}$$

The expressions for the virtual and the real cross sections, Eqs. (2.21) and (2.27), have been obtained setting $e_e=e$ and $g_e=g_s$. We reiterate that the fdh/dred computation can be done in a much simpler way by directly identifying these couplings from the beginning. The only place where it is crucial to distinguish them is for the proper UV (sub)renormalization, i.e. to obtain the counterterm in Eq. (2.18). If we had kept the couplings apart to the very end, the final result would have been unaffected. In other words, terms involving the ‘unphysical’ couplings $e_e$ and $g_e$ drop out when adding the virtual, the real, and the counterterms contributions. For our example this can easily be verified by using the expressions in Eqs. (2.14), (2.18), (2.19), and (2.22).

### Established properties and future developments of DS

As mentioned in the introduction, regularization schemes should not only simplify practical calculations but also satisfy certain basic requirements. For decades, dimensional regularization in the two flavours cdr and hv has been the most commonly used regularization, not only because it allows for the use of powerful calculational techniques but also because many all-order statements have been rigorously proved in these schemes.

Using an infinite-dimensional vector space as domain, a definition of the formally *d*-dimensional objects and operations is given in Refs. [3, 4]. Among the implications are mathematical consistency and the absence of possible ambiguities. The equivalence to bphz renormalization and the regularized and renormalized quantum action principle is shown in Refs. [15, 16]. As a caveat, however, in chiral theories these statements rely on the use of a non-anticommuting $\gamma _5$ as defined e.g. in Refs. [2, 16]. In non-chiral theories like QCD, the quantum action principle makes it obvious that non-Abelian gauge invariance is manifestly preserved such that the regularized QCD Green functions automatically satisfy the Slavnov–Taylor identities at all orders.

The situation regarding dred and fdh has been considerably more complicated in the past. However, now these schemes have reached a similar status as cdr and hv. After first one- and two-loop applications of dred [8], the equivalence of fdh/dred and cdr is shown in Refs. [9, 10], indirectly proving that these schemes are compatible with unitarity and causality. In Ref. [5], it is shown how the spaces in Eq. (2.3) can be defined in a rigorous way, avoiding mathematical ambiguities and excluding the possible inconsistency found before in Ref. [17]. In this way also an earlier puzzle regarding unitarity of dred discussed in Ref. [18] is resolved. The key ingredient for the solution is the introduction and separate treatment of $\epsilon$-scalar fields. One important consequence of the additional scalars is the need to distinguish gauge couplings from evanescent couplings during the renormalization procedure, as indicated in Eq. (2.7). The relation between unitarity and the correct renormalization of evanescent couplings in fdh/dred has been further stressed and exemplified with explicit calculations in Refs. [7, 11].

Apart from the UV properties of the dimensional schemes also IR divergences and their scheme dependence have been investigated up to the multi-loop regime. The separate treatment of $\epsilon$-scalars has been used in Ref. [19] to clarify a seeming non-factorization of QCD amplitudes observed earlier in Refs. [20–22]. In Refs. [6, 23], it is shown how dred and fdh can be applied in the computation of NLO cross sections in massless QCD. The scheme independence of a cross section at NLO has also been studied in Ref. [24]. Regarding virtual contributions, these considerations have been extended to NNLO in Refs. [12–14, 25, 26]. Moreover, the latter references provide NNLO transition rules for translating UV-renormalized virtual amplitudes from one dimensional scheme to another. The IR factorization properties of QCD including massive partons have been investigated at NLO in Ref. [27] and recently up to NNLO in Ref. [28]. For the real corrections, a formulation of the sector-improved residue subtraction scheme in the hv scheme is presented in Ref. [29].

Regarding supersymmetry, dred and fdh have significant advantages as in many cases supersymmetry is manifestly preserved although an all-order proof does not exist. For reviews regarding applications of these schemes to supersymmetry, we refer to Refs. [30, 31].

## FDF, SDF: four- and six-dimensional formalism

In the following we discuss some new (re-)formulations of ds. In Sects. 3.1–3.3, we describe fdf, a strictly four-dimensional formulation of the fdh scheme. The remaining two subsections are dedicated to topics that are not directly fdf but that are closely related to it, namely automated NLO calculations using GoSam and the six-dimensional formalism.

### FDF: four-dimensional formulation of FDH

The four-dimensional formulation of the fdh scheme (fdf) is a novel implementation of fdh. Its aim is to achieve the ${d}$-dimensional regularization of one-loop scattering amplitudes in a purely four-dimensional framework [32]. The starting point for the formulation of the scheme is the structure of the quasi-${d}_s$-dimensional fdh space, Eq. (2.3), which we write as

3.1 $$\begin{aligned} \text {QS}_{[{d_{s}}]}&=\text {QS}_{[{d}]}\oplus \text {QS}_{[n_\epsilon ]}\nonumber \\&=\text {S}_{[4]}\oplus \text {QS}_{[-2\epsilon ]}\oplus \text {QS}_{[n_\epsilon ]} \equiv \text {S}_{[4]}\oplus \text {QS}_{[n_\epsilon -2\epsilon ]} . \end{aligned}$$

Accordingly, the underlying space of the fdh scheme is written as an orthogonal sum of a strictly four-dimensional space $\text {S}_{[4]}$ and a quasi-$(n_\epsilon -2\epsilon )$-dimensional space $\text {QS}_{[n_\epsilon -2\epsilon ]}$. Similar to Eq. (2.5), metric tensors and $\gamma$ matrices can then be decomposed as

3.2 $$\begin{aligned} g_{[d_s]}^{\mu \nu }\ =\ g_{[4]}^{\mu \nu } + g_{[n_\epsilon -2\epsilon ]}^{\mu \nu } , \phantom {\bigg |} \quad \gamma _{[d_s]}^{\mu }\ =\ \gamma _{[4]}^{\mu } + \gamma _{[n_\epsilon -2\epsilon ]}^{\mu } , \phantom {\bigg |} \end{aligned}$$

with

3.3a $$\begin{aligned}&(g_{[4]}^{\phantom {\mu }})^{\mu }_{\phantom {\mu }\mu } = 4 \quad (g_{[4]}^{\phantom {\mu }} g_{[n_\epsilon -2\epsilon ]}^{\phantom {\mu }})^{\mu }_{\phantom {\mu }\nu } =0 , \end{aligned}$$

3.3b $$\begin{aligned}&(g_{[n_\epsilon -2\epsilon ]}^{\phantom {\mu }})^{\mu }_{\phantom {\mu }\mu } =(n_\epsilon -2\epsilon ) \mathop \rightarrow \limits _{}^{{d_s} \rightarrow 4} 0 .\qquad \end{aligned}$$

The algebraic properties of the matrices $\gamma ^\mu _{[n_\epsilon -2\epsilon ]}$ can be obtained from Eq. (3.3) and read

3.4a $$\begin{aligned}&\{\gamma ^{\mu }_{[n_\epsilon -2\epsilon ]}, \gamma ^{\nu \phantom {\mu }}_{[n_\epsilon -2\epsilon ]} \} = 2 g^{\mu \nu }_{[n_\epsilon -2\epsilon ]} ,\phantom {\bigg |} \end{aligned}$$

3.4b $$\begin{aligned}&\big \{ \gamma ^{\mu }_{[4]}, \gamma ^{\nu }_{[n_\epsilon -2\epsilon ]} \big \}=0 , \quad [ \gamma ^{5}_{[4]}, \gamma ^{\mu }_{[n_\epsilon -2\epsilon ]} ] = 0 , \end{aligned}$$

Loop momenta, on the other hand, are treated in *d* dimensions like in any dimensional scheme,

3.5 $$\begin{aligned} k_{[{d}]}^{\mu } = k_{[4]}^{\mu } + k_{[-2\epsilon ]}^{\mu } , \end{aligned}$$

with

3.6 $$\begin{aligned} k_{[{d}]}^2 = (k_{[4]}+k_{[-2\epsilon ]} )^2 = k_{[4]}^2+k_{[-2\epsilon ]}^2 \ \equiv \ k_{[4]}^2-\mu ^2 . \end{aligned}$$

Here and in the following, the square of the $( -2\epsilon )$-dimensional component of a loop momentum is identified with $-\mu ^2$. The decomposition of the space-time dimension in Eq. (3.6) then suggests that any integral of the form

3.7 $$\begin{aligned} I_{i_{1}\cdots i_{k}}^{d}[\mathcal {N}(k_{[{d}]})]&=\int \frac{{d}^{{d}}k_{[{d}]}}{(2\pi )^{{d}}} \frac{\mathcal {N}_{i_{1}\cdots i_{k}}(k_{[{d}]})}{D_{i_{1}}\cdots D_{i_{k}}} \end{aligned}$$

can be split according to

3.8 $$\begin{aligned}&I_{i_{1}\cdots i_{k}}^{d}[\mathcal {N}(k_{[4]},\mu ^{2})] = \int \frac{{d}^{4}k_{[4]}}{(2\pi )^{4}}\nonumber \\&\quad \times \int \frac{d^{-2\epsilon }k_{[-2\epsilon ]}}{(2\pi )^{-2\epsilon }} \frac{\mathcal {N}_{i_{1}\cdots i_{k}} (k_{[4]},\mu ^{2})}{D_{i_{1}}\cdots D_{i_{k}}} , \end{aligned}$$

where $i_{1}\dots i_{k}$ are indices labeling the loop propagators. With the decomposition of the integral measure in Eq. (3.8), any one-loop integral in ${d}$ dimensions has a four-dimensional integrand, depending on an additional length $\mu ^2$. The (radial) integration over $\mu ^2$ can be carried out algebraically by redefining the number of dimensions [33],

3.9 $$\begin{aligned} I_{i_{1}\cdots i_{k}}^{d}[(\mu ^{2})^{r}]&=(2\pi )^{r} I_{i_{1}\cdots i_{k}}^{d+2r} [1 ] \prod _{j=0}^{r-1}(d-4+2j) , \end{aligned}$$

so that powers of $\mu ^2$ in the numerator of the integrand generate integrals in shifted dimensions which are responsible for the *rational* terms of one-loop amplitudes.

We remark that an $(n_\epsilon -2\epsilon )$-dimensional metric tensor cannot have a four-dimensional representation. This is due to the fact that according to Eq. (3.3b), its square vanishes. Additionally, in four dimensions the only non-null matrices compatible with conditions (3.4) are proportional to $\gamma ^5_{[4]}$,

3.10 $$\begin{aligned} \gamma _{[n_\epsilon -2\epsilon ]}^{\phantom {\mu }} \sim \gamma ^5_{[4]} . \end{aligned}$$

However, the matrices $\gamma _{[n_\epsilon -2\epsilon ]}$ fulfill the Clifford algebra (3.4a), and thus

3.11 $$\begin{aligned}&\gamma ^{\mu }_{[n_\epsilon -2\epsilon ]} (\gamma _{[n_\epsilon -2\epsilon ]}^{\phantom {\mu }})_{\mu }\nonumber \\&\quad =(n_\epsilon -2\epsilon ) \mathop \rightarrow \limits _{}^{{d_s} \rightarrow 4}, \quad \text{ while } \quad (\gamma ^5_{[4]})^2 = \mathbb {I}_{[4]} . \end{aligned}$$

Equations (3.10) and (3.11) are therefore not compatible with each other. Finally, the component $k_{[-2\epsilon ]}^{\mu }$ of the loop momentum vanishes when contracted with a strictly four-dimensional metric tensor, i.e. $k^\mu _{[-2\epsilon ]} (g_{[4]}^{\phantom {\mu }})_{\mu \nu }= 0$. In four dimensions, the only four vector fulfilling this relation is the null one.

The above arguments exclude any four-dimensional representation of the $(n_\epsilon -2\epsilon )$- and $( -2\epsilon )$-dimensional subspaces. It is possible, however, to find a representation by introducing additional rules, in the following called $( -2\epsilon )$ *selection rules*, $( -2\epsilon )$-SRs. Indeed, the Clifford algebra (3.4a) is equivalent to3.12Therefore, any regularization scheme which is equivalent of fdh has to fulfill conditions (3.3)–(3.6), and (3.12). The orthogonality conditions (3.3) and (3.6) are fulfilled by splitting a $d_s$-dimensional vector field into a strictly four-dimensional one and a scalar field, while the other conditions are fulfilled by performing the substitutions

3.13 $$\begin{aligned} g^{\alpha \beta }_{[n_\epsilon -2\epsilon ]} \rightarrow G^{AB} , \quad \! \gamma ^\alpha _{[n_\epsilon -2\epsilon ]} \rightarrow \gamma ^5_{[4]} \Gamma ^A, \quad \! k^{\alpha }_{[-2\epsilon ]} \rightarrow i \mu Q^A . \phantom {\bigg |} \end{aligned}$$

The $(n_\epsilon -2\epsilon )$-dimensional and $( -2\epsilon )$-dimensional indices are thus traded for ($-2\epsilon$)-SRs such that

3.14 $$\begin{aligned}&G^{AB}G^{BC} = G^{AC} ,\quad G^{AA} = 0 ,\quad G^{AB} = G^{BA} , \nonumber \\&G^{AB} \Gamma ^A = \Gamma ^B ,\quad \Gamma ^{A} \Gamma ^A =0 ,\quad \{\Gamma ^A,\Gamma ^B\}=2 G^{AB} , \nonumber \\&G^{AB} Q^A = Q^B ,\quad Q^A Q^{A} =1 ,\quad Q^A \Gamma ^{A} =1 . \end{aligned}$$

The exclusion of terms containing odd powers of $\mu$ completely defines the fdf scheme. It allows one to build integrands which, upon integration, yield the same results as in the fdh scheme. As mentioned before, the fdf scheme is closely connected to the introduction of an additional scalar field. The role of this field and its relation to the $\epsilon$-scalar present in the fdh scheme will be discussed in Sect. 3.3.

The rules in Eq. (3.14) constitute an abstract algebra which is similar to an algebra related to internal symmetries. For instance, in a Feynman diagrammatic approach, the ($-2\epsilon$)-SRs can be handled as the colour algebra and performed for each diagram once and for all. In each diagram, the indices of the ($-2\epsilon$)-SRs are fully contracted and the outcome of their manipulation is either 0 or $\pm 1$. It is worth to remark that the replacement of $\gamma ^{\alpha }_{[n_\epsilon -2\epsilon ]}$ with $\gamma ^5_{[4]}$ takes care of the $d_s$-dimensional Clifford algebra automatically. Thus, we do not need to introduce any additional scalar field for each fermion flavour.

Depending on the gauge we use, further simplifications can arise. In Feynman gauge, for example, there are no contributions coming from scalar loops, which is due to the $( -2\epsilon )$-SRs,

3.15 $$\begin{aligned} G^{A_1A_2}G^{A_2A_3}\dots G^{A_{k}A_1} = G^{A_1A_1}=0 . \end{aligned}$$

Similarly, for diagrams with internal scalars and fermions we get the same cancellation,

3.16 $$\begin{aligned} \Gamma ^{A_1}G^{A_1A_2}\ldots G^{A_{k-1}A_k}\Gamma ^{A_{k}} = \Gamma ^{A_1}\Gamma ^{A_1}=0 . \end{aligned}$$

With the use of axial gauge, we obtain the opposite behaviour since contributions from internal scalars have to be taken in account,

3.17 $$\begin{aligned}&G^{A_1A_2}\hat{G}^{A_2A_3}\ldots G^{A_{k-1}A_k}\hat{G}^{A_{k}A_1} = G^{A_{1}A_2}\hat{G}^{A_2A_1}\nonumber \\&\quad = - Q^{A_1}Q^{A_1} =-1 , \end{aligned}$$

where $\hat{G}^{AB} \equiv G^{AB} - Q^A Q^B$. Diagrams that contain interactions between generalized gluons and scalars are dropped according to the $( -2\epsilon )$-SRs,

3.18 $$\begin{aligned} Q^{A_1}\hat{G}^{A_1A_2}\ldots Q^{A_{m}}\ldots \hat{G}^{A_kA_1} = \hat{G}^{A_{1}A_2}Q^{A_2} =0 . \end{aligned}$$

### Wave functions in FDF

Generalized-unitarity methods in dimensional regularization require an explicit representation of the polarization vectors and the spinors of $d_s$-dimensional particles. The latter ones are essential ingredients for the construction of the tree-level amplitudes that are sewn along the generalized cuts. In this respect, the fdf scheme is suitable for the four-dimensional formulation of *d*-dimensional generalized unitarity. The main advantage of fdf is that the four-dimensional expression of the propagators in the loop admits an explicit representation in terms of generalized spinors and polarization expressions which is collected below.

In the following discussion, the *d*-dimensional momentum $k_{[{d}]}$ will be put on-shell and decomposed according to Eq. (3.5). Its four-dimensional component, $k_{[4]}$, will be expressed as

3.19 $$\begin{aligned} k_{[4]} = k^\flat _{[4]} + \hat{q}_{[4]} , \quad \text {with}\quad \hat{q}_{[4]} \equiv \frac{m^2+\mu ^2 }{2 k_{[4]} \cdot q_{[4]}} q_{[4]} , \end{aligned}$$

in terms of the two massless momenta $k^\flat _{[4]}$ and $q_{[4]}$.

#### Spinors

The spinors of a $d_s$-dimensional fermion have to fulfill a completeness relation which reconstructs the numerator of the cut propagator, 3.20a
3.20b


The substitutions (3.13) allow one to express the r.h.s. of Eq. (3.20) as, 3.21a
3.21b in terms of generalized four-dimensional massive spinors defined as

3.22a $$\begin{aligned} u_{+} (k_{[4]} )&= | k^{\flat }_{[4]} \rangle +\frac{(m - i \mu )}{ [ k^{\flat }_{[4]} q_{[4]} ]} |q_{[4]} ] ,\nonumber \\ u_{-} (k_{[4]} )&= | k^{\flat }_{[4]}] +\frac{ (m + i \mu )}{ \langle k^{\flat }_{[4]} q_{[4]} \rangle } |q_{[4]} \rangle , \nonumber \\ v_{-} (k_{[4]} )&= | k^{\flat }_{[4]} \rangle -\frac{ (m + i \mu )}{ [ k^{\flat }_{[4]} q_{[4]} ]} |q_{[4]} ] ,\nonumber \\ v_{+} (l )&= | k^{\flat }_{[4]} ] -\frac{ (m - i \mu )}{ \langle k^{\flat }_{[4]} q_{[4]} \rangle } |q_{[4]} \rangle , \end{aligned}$$

3.22b $$\begin{aligned} \bar{u}_{+} (k_{[4]} )&= [k^{\flat }_{[4]} | +\frac{ (m + i \mu )}{\langle q_{[4]} k^{\flat }_{[4]} \rangle } \langle q_{[4]} | ,\nonumber \\ \bar{u}_{-} (k_{[4]} )&= \langle k^{\flat }_{[4]} | +\frac{ (m - i \mu )}{ [q_{[4]} k^{\flat }_{[4]} ]} [q_{[4]} | , \nonumber \\ \bar{v}_{-} (k_{[4]} )&= [k^{\flat }_{[4]} | -\frac{(m - i \mu )}{\langle q_{[4]} k^{\flat }_{[4]} \rangle } \langle q_{[4]} | ,\nonumber \\ \bar{v}_{+} (k_{[4]})&= \langle k^{\flat }_{[4]} | -\frac{(m + i \mu )}{[q_{[4]} k^{\flat }_{[4]}]} [q_{[4]}|. \end{aligned}$$

The spinors in Eq. (3.22a) are solutions of the tachyonic Dirac equations [34–37]3.23It is worth to notice that the spinors in Eq. (3.22) fulfill the Gordon identities

3.24 $$\begin{aligned} \frac{\bar{u}_\lambda (k_{[4]}^{\phantom {\nu }}) \; \gamma ^\nu _{[4]} \; u_\lambda (k_{[4]}^{\phantom {\nu }}) }{2} = \frac{\bar{v}_\lambda (k_{[4]}^{\phantom {\nu }}) \; \gamma ^\nu _{[4]} \; v_\lambda (k_{[4]}^{\phantom {\nu }}) }{2} = k_{[4]}^\nu . \end{aligned}$$

#### Polarization vectors

The $d_s$-dimensional polarization vectors of a spin-1 particle fulfill the relation

3.25 $$\begin{aligned} \sum _{i=1}^{d_s -2} \varepsilon _{i, [d_s]}^\mu ( k_{[{d}]},\eta ) \varepsilon _{i, [d_s]}^{*\nu }( k_{[{d}]} , \eta ) = - g^{\mu \nu }_{[d_s]} +\frac{ k_{[{d}]}^\mu \eta ^\nu + k_{[{d}]}^\nu \eta ^\mu }{ k_{[{d}]} \cdot \eta } , \end{aligned}$$

where $\eta$ is an arbitrary *d*-dimensional massless momentum such that $k \cdot \eta \ne 0$. Gauge invariance in *d* dimensions guarantees that the cut is independent of $\eta$. In particular the choice

3.26 $$\begin{aligned} \eta ^\mu = k^\mu _{[4]} - k^\mu _{[-2\epsilon ]} , \end{aligned}$$

allows one to disentangle the four-dimensional contribution from the $( -2\epsilon )$-dimensional one:

3.27 $$\begin{aligned}&\sum _{i=1}^{d_s -2} \varepsilon _{i (d_s)}^\mu \left( k , \eta \right) \varepsilon _{i (d_s)}^{*\nu }\left( k , \eta \right) =\left( - g^{\mu \nu }_{[4]} +\frac{ k^\mu _{[4]} k^\nu _{[4]}}{\mu ^2} \right) \nonumber \\&\quad -\left( g^{\mu \nu }_{[n_\epsilon -2\epsilon ]} +\frac{ k^\mu _{[-2\epsilon ]} k^\nu _{[-2\epsilon ]}}{\mu ^2} \right) . \end{aligned}$$

The first term is related to the cut propagator of a massive gluon and can be expressed as

3.28 $$\begin{aligned} - g^{\mu \nu }_{[4]} +\frac{ k^\mu _{[4]} k^\nu _{[4]}}{\mu ^2}&= \sum _{\lambda =\pm ,0}\varepsilon _{\lambda }^{\mu }(k_{[4]}) \varepsilon _{\lambda }^{*\nu }(k_{[4]}) \end{aligned}$$

in terms of the four-dimensional polarizations of a vector boson of mass $\mu$ [38],

3.29 $$\begin{aligned}&\varepsilon _{+}^{\mu } (k_{[4]} ) = -\frac{ [k^{\flat }_{[4]} \left| \gamma ^{\mu }\right| \hat{q}_{[4]} \rangle }{\sqrt{2}\mu } ,\nonumber \\&\varepsilon _{-}^{\mu } (k_{[4]} ) = - \frac{ \langle k^{\flat }_{[4]} \left| \gamma ^{\mu }\right| \hat{q}_{[4]} ]}{\sqrt{2}\mu } , \quad \varepsilon _{0}^{\mu } (k_{[4]} ) = \frac{k^{\flat \mu }_{[4]}-\hat{q}_{[4]}^{\mu }}{\mu } .\nonumber \\ \end{aligned}$$

The latter fulfill the well-known relations

3.30 $$\begin{aligned} \varepsilon ^2_{\pm }(k_{[4]})&=\phantom {-} 0 , \quad \varepsilon _{\pm }(k_{[4]})\cdot \varepsilon _{\mp }(k_{[4]})=-1 ,\nonumber \\ \varepsilon _{0}^2(k_{[4]})&=-1 , \nonumber \\ \varepsilon _{\pm }(k_{[4]})\cdot \varepsilon _{0}(k_{[4]})&=\phantom {-} 0 , \quad \varepsilon _{\lambda }(k_{[4]}) \cdot k_{[4]} =\phantom {-} 0 . \end{aligned}$$

The second term of the r.h.s. of Eq. (3.27) is related to the numerator of cut propagator of the scalar and can be expressed in terms of the $( -2 \epsilon )$-SRs as:

3.31 $$\begin{aligned} g^{\mu \nu }_{[n_\epsilon -2\epsilon ]} +\frac{ k^\mu _{[-2\epsilon ]} k^\nu _{[-2\epsilon ]}}{\mu ^2} \rightarrow \hat{G}^{AB} \equiv G^{AB} - Q^A Q^B . \end{aligned}$$

Therefore, we can define the cut propagators as3.32The generalized four-dimensional spinors and polarization vectors defined above can be used for constructing tree-level amplitudes with full $\mu$-dependence.

### Established properties and future developments of FDF

At one-loop, fdf has been successfully applied to compute the scattering amplitudes for multi-gluon scattering $g g\rightarrow n$ gluons with $n=2,3,4$, and for $g g\rightarrow H + n\ \text {gluons}$ with $n=2,3$ [39, 40]. The use of dimensionally regularized tree-amplitudes within fdf has been employed to study the colour-kinematics duality [41] for one-loop dimensionally regularized amplitudes [42].

The extension of fdf beyond the one-loop level is currently under investigation. In particular at two loops, fdf should be able to capture the dependence of the integrand on the extra dimensional terms of the loop momenta, namely on two mass-like variables, say $\mu _1^2$ and $\mu _2^2$, as well as on the scalar product $\mu _1\cdot \mu _2$.

#### Equivalence of FDF and FDH at NLO: virtual contributions to $e^{+}e^{-}\rightarrow \gamma ^{*}\rightarrow q\bar{q}$

To show that the strictly four-dimensional Feynman rules of fdf together with the $( -2\epsilon )$-SRs indeed reproduce the corresponding results in the fdh scheme for $\alpha _e=\alpha _s$, we consider virtual one-loop contributions to the process $e^{+}e^{-} \rightarrow \gamma ^{*} \rightarrow q\bar{q}$.

**Fig. 6:**
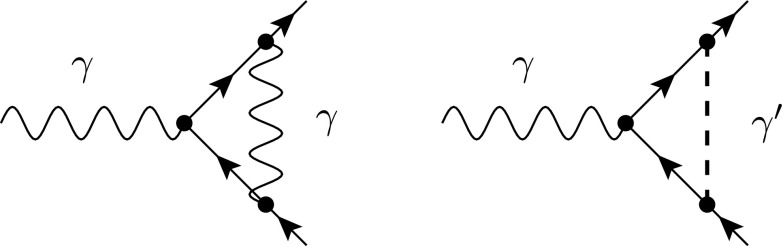
Virtual diagrams contributing to $\gamma ^{*}\rightarrow q\bar{q}$ at NLO including a strictly four-dimensional photon $\gamma$ (*wavy line*) and an fdf scalar $\gamma '$ (*dashed line*), respectively. Using Feynman gauge, the right diagram vanishes according to the $( -2\epsilon )$-SRs

According to the discussion in Sect. 3.1, in fdf each vector field is split into a strictly four-dimensional field and a corresponding scalar field. The vertex correction subgraph $\gamma ^{*} \rightarrow q {\bar{q}}$ therefore receives two contributions in fdf; see Fig. 6. The diagram including an internal fdf-scalar vanishes according to the $(-2\epsilon )$-SRs since in Feynman gauge it is proportional to $\Gamma ^A\Gamma ^B G^{AB}=\Gamma ^A\Gamma ^A=0$. Using only strictly four-dimensional quantities, the amplitude is then given by3.33

where $p_q$ and $p_{\bar{q}}$ denote the four-momenta of the massless quarks. Evaluating the strictly four-dimensional algebra and performing a tensor integral decomposition in ${d}$ dimensions, the amplitude can be written as

3.34 $$\begin{aligned} (\mathcal{A}^{(1)}_{{{\textsc {fdf}}}})_{\mu } =-i (\mathcal{A}^{(0)}_{{{\textsc {fdf}}}})_{\mu } g_s^2 {C_F}\bigg \{ \frac{{d}}{{d}-4} I_{2}^{{d}}[1] -2 I_{3}^{{d}}[\mu ^2] \bigg \} , \end{aligned}$$

with

3.35a $$\begin{aligned} I_{2}^{{d}}[1]&=\int \frac{{d}^{{d}} k_{[{d}]}}{(2 \pi )^{{d}}} \frac{1}{ (k_{[{d}]}+p_{q,[{d}]})^2 (k_{[{d}]}-p_{\bar{q},[{d}]})^2} , \end{aligned}$$

3.35b $$\begin{aligned} I_{3}^{{d}}[\mu ^2]&=\int \frac{{d}^{{d}} k_{[{d}]}}{(2 \pi )^{{d}}} \frac{\mu ^2}{ (k_{[{d}]}+p_{q,[{d}]})^2 (k_{[{d}]}-p_{\bar{q},[{d}]})^2 (k_{[{d}]})^2} . \end{aligned}$$

Note that in the denominators we used Eq. (3.6). In this way, the integral in Eq. (3.35a) is an ordinary ${d}$-dimensional one. The integral in Eq. (3.35b), on the other hand, can be evaluated by using Eq. (3.9),

3.36 $$\begin{aligned} I_{3}^{{d}}[\mu ^2] =(2\pi )(-2\epsilon ) I_{3}^{{d}+2}[1] =\frac{i}{(4\pi )^2}\frac{1}{2}+\mathcal {O}(\epsilon ) . \end{aligned}$$

For the virtual corrections to the (spin summed/averaged) squared matrix element $M_{{{\textsc {fdf}}}}^{(1)}=2{\text {Re}} \langle \mathcal {A}_{{{\textsc {fdf}}}}^{(0)} | \mathcal {A}_{{{\textsc {fdf}}}}^{(1)}\rangle$, we then obtain^5^

3.37 $$\begin{aligned} M^{(1)}_{{{\textsc {fdf}}}} =\omega ^{(1)} M^{(0)}_{{{\textsc {fdf}}}} \bigg (\frac{\alpha _s}{\pi }\bigg ) \bigg [-\frac{1}{\epsilon ^2}-\frac{3}{2 \epsilon }-\frac{7}{2} +\mathcal {O}(\epsilon )\bigg ] . \end{aligned}$$

**Fig. 7:**
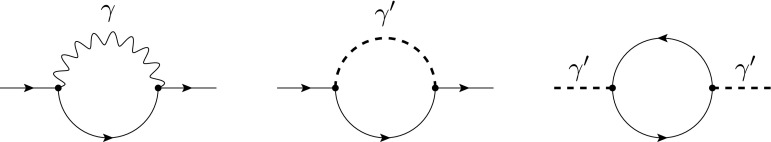
One-loop diagrams contributing to the self-energy of the quark (*left* and *middle*) and of the fdf-scalar (*right*). The diagram with the internal fdf scalar vanishes according to the $(-2\epsilon )$-SRs

#### Renormalization of the FDF-scalar–fermion coupling

In the following we determine the $\beta$ function related to the coupling of the fdf-scalar to fermions in QED with $N_F$ fermion flavours, and compare it to the known renormalization of the gauge and the evanescent coupling in the fdh scheme given in Eq. (2.8).

To start, we consider the fermion self-energy, where two diagrams contribute at the one-loop level; see Fig. 7. Using the Feynman rules of Ref. [32] together with the $( -2\epsilon )$-SRs, we obtain for the case of massless fermions3.38

**Fig. 8:**
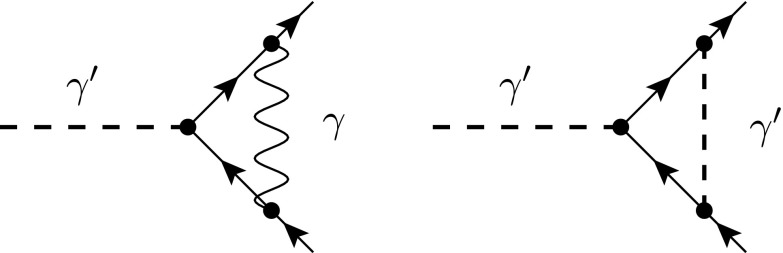
Diagrams contributing to the interaction of the fdf scalar with fermions at the one-loop level. The *right diagram* vanishes according to the $(-2\epsilon )$-SRs

In particular, we applied relation (3.6) and made use of the fact that terms containing odd powers of $\mu$ are set to zero. The diagram including an internal fdf-scalar vanishes according to the $( -2\epsilon )$-SRs since it is proportional to $\Gamma ^A\Gamma ^B G^{AB}=\Gamma ^A\Gamma ^A=0$. Evaluating the ${d}$-dimensional integral in Eq. (3.38), we then obtain^6^
3.39Using minimal subtraction, the renormalization of the fermion field is therefore given by

3.40a $$\begin{aligned} Z_2 =1 +\bigg (\frac{\alpha }{4\pi }\bigg )\bigg [-\frac{1}{\epsilon }\bigg ] +\mathcal {O}(\alpha ^2) . \end{aligned}$$

A calculation similar to Eq. (3.38) yields for the renormalization of the fdf-scalar field

3.40b $$\begin{aligned} Z_{3}' =1 +\bigg (\frac{\alpha }{4\pi }\bigg )\bigg [-\frac{2}{\epsilon } N_F\bigg ] +\mathcal {O}(\alpha ^2) . \end{aligned}$$

Finally, we consider the vertex correction. Again, in fdf two diagrams contribute at the one-loop level; see Fig. 8. According to the $( -2\epsilon )$-SRs, the diagram with an internal fdf-scalar is proportional to $\Gamma ^B \Gamma ^A \Gamma ^B =-\Gamma ^B \Gamma ^B \Gamma ^A+2 \Gamma ^B G^{AB}= 2 \Gamma ^{A}$. Evaluating the strictly four-dimensional Lorentz algebra and performing the ${d}$-dimensional loop integration, the renormalization of the vertex is given by

3.40c $$\begin{aligned} Z_{1}' =1 +\bigg (\frac{\alpha }{4\pi }\bigg )\bigg [-\frac{4}{\epsilon }\bigg ] +\mathcal {O}(\alpha ^2) . \end{aligned}$$

In a similar way, the renormalization constants can be obtained for the case of massive fermions. In the on-shell scheme (os) they read,^7^

3.41a $$\begin{aligned} Z_{2} |_{{{{\textsc {os}}}}}&=1+\bigg (\frac{\alpha }{4\pi }\bigg ) \bigg [ -\frac{3}{\epsilon } +\text {ln}\bigg (\frac{m_e^2}{\mu ^2}\bigg ) -5\bigg ] +\mathcal {O}(\alpha ^2) , \end{aligned}$$

3.41b $$\begin{aligned} Z_{3}' |_{{{{\textsc {os}}}}}&=1+\bigg (\frac{\alpha }{4\pi }\bigg ) N_F \bigg [ -\frac{2}{\epsilon } +2 \text {ln}\bigg (\frac{m_e^2}{\mu ^2}\bigg ) -\frac{2}{3}\bigg ] +\mathcal {O}(\alpha ^2) , \end{aligned}$$

3.41c $$\begin{aligned} Z_{1}' |_{{{{\textsc {os}}}}}&=1+\bigg (\frac{\alpha }{4\pi }\bigg ) \bigg [ -\frac{4}{\epsilon } +4 \text {ln}\bigg (\frac{m_e^2}{\mu ^2}\bigg ) -8\bigg ] +\mathcal {O}(\alpha ^2) . \end{aligned}$$

Combining the results in Eqs. (3.40) or (3.41), the $\beta$ function of the fdf-scalar coupling to fermions is finally given by

3.42 $$\begin{aligned} \beta ' =-\bigg (\frac{\alpha }{4\pi }\bigg )^2 [ 2-2 N_F ] +\mathcal {O}(\alpha ^3) , \end{aligned}$$

and therefore identical to the renormalization of the evanescent coupling in fdh for $e_e=e$, compare with Eq. (2.8b). According to the discussion in Sect. 2.2, the different renormalization of the couplings in the fdh scheme (and therefore in fdf) does not play any role at the one-loop level. At higher perturbative orders, however, it can lead to a breaking of unitarity [25]. The way, how the different renormalization of the scalar coupling can be consistently implemented beyond one loop in the fdf framework is currently under investigation.

### Automated numerical computation

To build a fully consistent procedure that is valid for every Lagrangian is an issue for the complete automation of higher order computations via numerical recipes. In the GoSam [43] actual architecture we adopted a scheme that naturally produces results in fdh.^8^ In this scheme, GoSam can generate the full one-loop amplitude for every process originating from every Lagrangian with the only condition that the power of the loop momentum in the numerator of a diagram cannot exceed the number of loop denominators plus one. On the other hand, we still do not have a completely general procedure for the renormalization. Technically, the algebraic implementation of our procedure is extremely simple and can be summarized in the following three points:

1. Assume that all Lorentz indices are four-dimensional, even if in a following step the loop momentum *k* will be treated as *d*-dimensional.
2. In all fermion chains, also in fermion loops, bring all chiral projectors to the left and all loop momenta to the right.
3. Apply the rule

This is a simplified version of what is effectively coded, which has the same algebraic content and produces the same result. The $\mu ^2$ parameter represents the length of the loop momentum into the $\epsilon$-dependent dimensions.

In GoSam, the generation of amplitudes starts from diagram generation with QGRAF [44] that searches for topologies and fills them with fields in all possible ways. This construction paired with the few rules given above guarantees that no spurious anomalies are generated and, most important, it provides the correct result for all the computations that are anomaly free. In full generality, for every diagram we are then left with two ingredients: a number of non-vanishing integrals with $\mu ^2$, and a polynomial of the four-dimensional part of the loop momentum sitting on every number of denominators. Loop integrals with $\mu ^2$ in the numerator have been computed analytically since long, so that their implementation is trivial. Furthermore, reduction programs like Golem95 [45, 46], Ninja [47–49] or Samurai [50] reduce them easily. The polynomial in the four-dimensional component of the loop momentum is the optimal representation of the loop integral for the numerical reduction with programs like CutTools [51], Golem95, Ninja or Samurai.

When we are computing higher-order differential cross sections using some subtraction scheme [52, 53] to regularize IR divergences, the choice of the dimensional scheme adopted is restricted to the virtual integration, and one can exploit unitarity to derive the transition rules among renormalized amplitudes computed in different (unitary) schemes; see Refs. [23, 24] for more details. For this reason it is trivial to derive transition rules from fdh to cdr for example deducing them from the different finite part of the integrated dipoles computed in the two schemes. We refer to the dipoles-subtraction technique, but the reasoning is completely general and provides the same conversion factors irrespective of the subtraction scheme. To be definite, to convert a one-loop amplitude in the Standard Model, one can start from the massless gauge-boson emissions from QCD radiation to determine the shift as $n_{lq} C_F/2 + n_g C_A/6$ times the underlying tree-level interference, where $n_{lq}(n_g)$ is the number of the external light quarks (gluons) being part of the hard scattering amplitude. This agrees with the shift found in Ref. [23]. Similarly, for QED radiation the shift is again the underlying tree-level interference times the sum of factors $\delta _{\text {RS}}=-q_i \sigma _i q_k \sigma _k/2$ for each pair of emitter (*i*) with electric charge $q_i$ and spectator (*k*) with electric charge $q_k$ and $\sigma$ being $1 (-1)$ for an incoming fermion and outgoing anti-fermions (vice versa).

Now we come to the renormalization. In GoSam, this is still not fully automated. For the QCD part of the Lagrangian that is renormalized with the ${\overline{{{\textsc {ms}}}}}$ prescription, subtracting only the poles, with fdh or dred one is left with a different definition for the renormalized coupling constants w.r.t. cdr. A finite renormalization is needed to restore the customary definition (cdr). There is of course no such problem with the on-shell renormalization that is often used for electroweak corrections. In GoSam we computed and implemented all the renormalization constants of the Standard Model Lagrangian and derived the conversion factors from fdh to cdr. They can be found in Ref. [54].

To conclude, the fdh scheme appears optimal for numerical computations and the conversion rules to other schemes can easily be worked out once and for all exploiting unitarity. Finally, we stress that on the path towards fully automated computations for every Lagrangian, the automated computation of the renormalization constants is mandatory.

### SDF: six-dimensional formalism

In this section we discuss the possibility of implementing dimensional regularization schemes via an embedding of the loop degrees of freedom in a ${d_{e}}$-dimensional space, where ${d_{e}}$ (*e* stands for embedding) is an integer greater than 4 which depends on the loop order. This is possible in dimensional schemes such as fdh and hv, where the degrees of freedom of the external particles live in the genuine four-dimensional space $S_{[4]}$. In particular, we focus on the case ${d_{e}}=6$, which is sufficient up to two loops [55].

Having a finite integer-dimensional embedding of the loop degrees of freedom is especially useful in the context of integrand reduction via generalized unitarity [56–64], which provides an efficient way of generating loop integrands from products of tree-level amplitudes summed over the internal helicity states. In particular, the possibility of using a ${d_{e}}$-dimensional spinor-helicity formalism provides a finite-dimensional (six-dimensional in our case) representation of both external and internal states. The six-dimensional spinor-helicity formalism has been extensively developed in Ref. [65], and used in the context of multi-loop generalized unitarity for producing analytic results for five- and six-point two-loop all-plus amplitudes in (non-supersymmetric) Yang–Mills theory [64, 66, 67].

A useful property of this approach is that it gives both internal and external states an explicit finite-dimensional representation. This means that one can perform both analytic and numerical calculations by working directly with the components of momenta and spinors. Numerical calculations can in turn be used to infer properties of the result before a full analytic calculation, or in order to employ functional reconstruction techniques (see e.g. Ref. [68]) which allow one to reconstruct full analytic results from numerical calculations over finite fields.

As mentioned, in this section we focus on a dimensional regularization scheme where the external states live in the physical four-dimensional space $$\text {S}_{[4]}$$, while we keep the dimension $$d_s$$ of the space $$\text {QS}_{[d_s]}$$ undetermined. The special cases of fdh and hv can be obtained by setting $$d_s =4 $$ and $$d_s =d$$, respectively, at the end of the calculation.

#### Internal degrees of freedom

We consider a generic contribution to an $\ell$-loop amplitude

3.43 $$\begin{aligned} \int \limits _{-\infty }^{\infty } \left( \prod _{i=1}^\ell {d}^d k_i\right) \frac{\mathcal {N}(k_i)}{\prod _j D_j(k_i)} , \end{aligned}$$

where $\mathcal {N}$ and *D* are polynomials in the components of the loop momenta $k_i$ (a rational dependence on the external kinematic variables is always understood). In particular, the denominators $D_i$ correspond to loop propagators and have the generic quadratic form

3.44 $$\begin{aligned}&D_i = \ell _i^2 - m_i^2 ,\quad l_i^\mu = \sum _{j=1}^\ell \alpha _{ij} k_j^\mu + \sum _{j=1}^n \beta _{ij} p_j^\mu ,\nonumber \\&\alpha _{ij},\beta _{ij}\in \{0,\pm 1\} , \end{aligned}$$

with $p_j$ being the external momenta. It is often useful to split the loop momenta $k_i^\mu$ into a four-dimensional part $k_{i,[4]}^\mu$ and a $(d -4)$-dimensional part $k_{i,[d-4]}^\mu$ as

3.45 $$\begin{aligned} k_i^\mu = k_{i,[4]}^\mu + k_{i,[d-4]}^\mu . \end{aligned}$$

In a regularization scheme where the external states are four-dimensional, a loop integrand can only depend on the $(d -4)$ extra-dimensional components of each loop through scalar products $\mu _{ij}$ defined as

3.46 $$\begin{aligned} \mu _{ij} = - ( k_{i,[d-4]}\cdot k_{j,[d-4]} ) . \end{aligned}$$

The scalar products $\mu _{ij}$ can in turn be reproduced by embedding the loop momenta in an integer-dimensional space with dimension ${d_{e}}\ge 4+ \ell$. In particular, as stated, the choice ${d_{e}}=6$ is sufficient up to two loops. Although we will focus on the case ${d_{e}}=6$ and scattering amplitudes at one loop or two loops, unless stated otherwise our statements are valid for any multi-loop amplitude, provided that the integer ${d_{e}}$ is sufficiently large.

In order to correctly reconstruct the dependence of the integrand on the dimension $d_s$ of the space $\text {QS}_{[d_s]}$ where internal gluon polarizations live, we add $({d}_s -{d_{e}})$ flavours of scalar particles to the theory, which represent gluon polarizations orthogonal to both the external and the loop momenta. The Feynman rules for these scalars can easily be derived from the ones of gluons (see e.g. Ref. [64]).

#### Internal states: six-dimensional spinor-helicity formalism

External states of helicity amplitudes can be efficiently described using the well-known four-dimensional spinor-helicity formalism [69, 70]. After a higher-dimensional embedding of internal states, one can similarly describe these by means of a higher-dimensional spinor-helicity formalism. In particular, the spinor-helicity formalism in six dimensions has been developed in Refs. [65, 71, 72]. While a comprehensive treatment of the subject is beyond the purpose of this report (we refer the reader to Ref. [65] for more details), it is worth pointing out a few properties of six-dimensional spinors which are useful for providing an integer-dimensional embedding of the loop internal states, in particular for applications in the context of integrand reduction via generalized unitarity, as we shall see in the next section.

Six-dimensional Weyl spinors $|p^a\rangle$ and $|p_{\dot{a}}]$ (with $a,\dot{a} \in \{0,1\}\equiv \{+,-\}$) are defined as independent solutions of the six-dimensional Dirac equation

3.47 $$\begin{aligned} p^\mu \sigma ^{(6)}_\mu |p^a\rangle = p^\mu \tilde{\sigma }^{(6)}_\mu |p_{\dot{a}}] = 0 , \end{aligned}$$

where $\sigma ^{(6)}_\mu$ and their dual $\tilde{\sigma }^{(6)}_\mu$ are six-dimensional generalizations of the Pauli matrices (see Ref. [65] for an explicit representation). Six-dimensional momenta can be built from spinors,

3.48 $$\begin{aligned} p^\mu = -\frac{1}{4} \langle p^a|\sigma ^\mu |p^b\rangle \epsilon _{a b} ,\quad p^\mu = -\frac{1}{4} [ p_{\dot{a}} | \tilde{\sigma }^\mu | p_{\dot{b}} ] \epsilon ^{\dot{a} \dot{b}} . \end{aligned}$$

Similarly, given a six-dimensional momentum $p^\mu$, a representation for the spinors $|p^a\rangle$ and $|p_{\dot{a}}]$ satisfying the previous equations, while not unique, is not hard to find. Note that, when building loop integrands, the internal spinors always combine as on the r.h.s. of Eq. (3.48), hence the physical results are always unambiguous and independent of the chosen representation. Moreover, a subset of the six-dimensional spinor components can be identified with the components of four-dimensional Weyl spinors $|p\rangle$ and |*p*], which ensures a smooth four-dimensional limit.

Internal gluon states are described by six-dimensional polarization vectors, which can be built out of these spinors

3.49 $$\begin{aligned} \epsilon ^\mu _{a \dot{a}}(p,\eta ) = \frac{1}{\sqrt{2} (p\cdot \eta )} \langle p_a | \sigma ^\mu | \eta _{b} \rangle \langle \eta _c | p _{\dot{a}} ] \epsilon ^{b c} \end{aligned}$$

with

3.50 $$\begin{aligned}&(a \dot{a}) \in \{(00), (11), (01), (10) \} \nonumber \\&\quad \equiv \{(++), (--), (+-), (-+) \} . \end{aligned}$$

While $(++)$ and $(--)$ correspond to positive and negative helicity in the four-dimensional limit, respectively, the polarizations $(+-)$, $(-+)$ only exist in six dimensions. One can show [65] that these polarization vectors satisfy all the expected properties, including the completeness relation

3.51 $$\begin{aligned} \epsilon ^\mu _{a \dot{a}}(p,\eta ) \epsilon ^\nu {}^{a \dot{a}}(p,\eta ) = g^{\mu \nu } - \frac{1}{(p\cdot \eta )} ( p^\mu \eta ^\nu + p^\nu \eta ^\mu ) . \end{aligned}$$

When building an integrand via generalized unitarity, internal polarization states always combine as on the l.h.s. of the previous equation.

#### Applications to integrand reduction via generalized unitarity

Integrand reduction methods rewrite loop integrands as a sum of irreducible contributions,

3.52 $$\begin{aligned} \frac{\mathcal {N}(k_i)}{\prod _j D_j(k_i)} = \sum _{T} \frac{\Delta _T(k_i)}{\prod _{j\in T} D_j(k_i)} , \end{aligned}$$

where the sum on the r.h.s. runs over the non-vanishing sub-topologies of the parent topology identified by a set of denominators $\{D_j\}$. The on-shell numerators or residues $\Delta _T$ can be written as a linear combination of polynomials $\mathbf {q}_T =\{q_{T,1}, q_{T,2},\ldots \}$ which can be combined to form an integrand basis up to terms proportional to the denominators of the corresponding sub-topology *T*,

3.53 $$\begin{aligned} \Delta _{T}(k_i) = \sum _{\alpha } c_{T,\alpha } (\mathbf {q}_T(k_i))^\alpha ,\quad \mathbf {q}_T^\alpha \equiv \prod _j q_{T,j}^{\alpha _j} , \end{aligned}$$

where $\alpha =(\alpha _1,\alpha _2,\ldots )$ runs over an appropriate set of multi-indices. Techniques for choosing an appropriate integrand basis have been proposed e.g. in Refs. [61–63, 67].

The coefficients $c_{T,\alpha }$ only depend on the external kinematics (they also have a polynomial dependence on $d_s$) and they can be determined by evaluating the integrand on values of the loop momenta such that the propagators of the corresponding loop sub-topology are put on-shell $\{D_j=0\}_{j\in T}$. These constraints are also known as multiple cuts. On these values of the loop momenta, the integrand factorizes as a product of tree-level amplitudes summed over the internal helicities corresponding to the cut on-shell loop momenta. Hence, an efficient way of computing the integrands on the cut conditions is by sewing together tree-level amplitudes. This is known as generalized unitarity. As explained, by means of a higher-dimensional spinor-helicity formalism, one can build products of trees which contain the full dependence of the integrand on the loop degrees of freedom.

More explicitly, the solutions of the cut conditions in ${d_{e}}$ dimensions can be expressed as a linear combination of terms of a ${d_{e}}$-dimensional vector basis $\{e_{ij}\}_{j=1}^{d_{e}}$,

3.54 $$\begin{aligned} k_i^\mu = \sum _{j=1}^{d_{e}}y_{ij} e_{ij}^\mu , \end{aligned}$$

where, in turn, the coefficient of this linear combination can be expressed as $y_{ij}=y_{ij}(\{\tau _k\})$, where $\{\tau _k\}$ is a set of free variables which are not constrained by the cut conditions. From these ${d_{e}}$-dimensional on-shell momenta, we thus build the corresponding ${d_{e}}$-dimensional spinors, which in turn are used to evaluate the tree-level helicity amplitudes which define the integrand on the considered multiple cut.

As we mentioned, the correct dependence of the integrand on $d_s$ is obtained by adding to the theory $({d}_s -{d_{e}})$ flavours of scalars representing additional polarizations of the internal gluons. At two loops, an integrand can have at most a quadratic dependence on scalar flavours

3.55 $$\begin{aligned} \Delta _T = \Delta _T^{({d_{e}},0)} + (d_s-{d_{e}}) \Delta _T^{({d_{e}},1)} + (d_s-{d_{e}})^2 \Delta _T^{({d_{e}},2)} . \end{aligned}$$

More in general, each scalar loop can add at most one power of $({d}_s -{d_{e}})$. We stress that the result for $\Delta _T$ does not depend on the dimension ${d_{e}}$ of the chosen embedding, unlike each of the terms on the r.h.s. of the previous equation.

This setup has been used for the calculation of planar five- and six-point two-loop amplitudes in Yang–Mills theory presented in Refs. [64, 66, 67], as well as for the first application of multivariate reconstruction techniques to generalized unitarity presented in Ref. [68]. The latter includes the calculation of the on-shell integrands of the maximal cuts of the two-loop planar pentabox and the non-planar double pentagon topology, for a complete set of independent helicity configurations. This shows that this strategy is suitable for performing complex multi-leg calculations at two loops, which is currently a very active field of research.

## IREG: implicit regularization

### Introduction to IREG and electron self-energy at NLO

Implicit regularization (ireg) is a regularization framework proposed by the end of the 1990s [73–75] as an alternative to well-known dimensional schemes. A main characteristic of the method is that it stays in the physical dimension of the underlying quantum field theory, avoiding, in principle, some of the drawbacks of ds such as the mismatch between fermionic and bosonic degrees of freedom which leads to the breaking of supersymmetry. ireg is proposed to work in momentum space and relies on the following observation: the UV divergent piece of any Feynman integral should not depend on physical parameters such as external momenta or particles masses.^9^ This simple fact leads to profound consequences as we are going to see.

For ease of the reader, we will develop the basic concepts of ireg by considering a familiar example of massless QED, the one-loop corrections to the fermion propagator. We write the initial (unregularized) expression as4.1where *p* is an external momentum. The first step is to perform simplifications using Dirac algebra in strictly four dimensions. In this example, the result is particularly simple

4.2 $$\begin{aligned} -i \Sigma ^{(1)}(p) =2 e^2 \gamma _{\mu }\int \frac{{d}^4k}{(2 \pi )^4} \frac{k^{\mu }}{k^2(k-p)^{2}} . \end{aligned}$$

The next step is just to introduce a fictitious mass in the propagators which will allow us to control spurious IR divergences introduced in the course of the evaluation. Thus, the integral can be rewritten as

4.3 $$\begin{aligned}&-i \Sigma ^{(1)}(p) \nonumber \\&\quad =\lim _{\mu ^{2}\rightarrow 0} 2 e^2 \gamma _{\mu }\int \frac{{d}^4k}{(2 \pi )^4} \frac{k^{\mu }}{(k^2-\mu ^{2})[(k-p)^{2}-\mu ^{2}]}\nonumber \\&\quad \equiv \lim _{\mu ^{2}\rightarrow 0} [ -i \Sigma ^{(1)}_{{{\textsc {ireg}}}}(p,\mu ) ] . \end{aligned}$$

At this point one uses the main observation of iregthat the intrinsic divergent integral should not depend on physical parameters, the external momentum in this case. To achieve that, one just uses the following identity as many times as necessary to isolate the physical parameters in the finite part:

4.4 $$\begin{aligned} \frac{1}{(k-p)^2-\mu ^2} =\frac{1}{(k^2-\mu ^2)} +\frac{(-1)(p^2-2 p \cdot k)}{(k^2-\mu ^2) [(k-p)^2-\mu ^2 ]} . \end{aligned}$$

In our example, one ends up with the following divergent expression:

4.5 $$\begin{aligned} -i \Sigma _{{{\textsc {ireg}}}}^{(1)}(p,\mu ) |_{\text {div}}&=2 e^2 \gamma _{\mu }\Bigg [ \int \frac{{d}^4k}{(2 \pi )^4}\frac{k^{\mu }}{(k^{2}-\mu ^{2})^{2}}\nonumber \\&\quad +2 p_{\nu }\int \frac{{d}^4k}{(2 \pi )^4}\frac{k^{\mu }k^{\nu }}{(k^{2}-\mu ^{2})^{3}} \Bigg ] , \end{aligned}$$

in which all dependence on the external momenta is only in the numerator. The latter can be therefore pulled outside the integration. Focusing on the divergences, one notices the existence of linear and logarithmic terms. The first piece is automatically null (as in cdr) and we are left with only the logarithmic term, whose integral is a particular example of the general expression

4.6 $$\begin{aligned} I_{\text {log}}^{\nu _{1}\cdots \nu _{2N}}(\mu ^{2}) \equiv \int \frac{{d}^4k}{(2 \pi )^4} \frac{k^{\nu _{1}}\cdots k^{\nu _{2N}}}{(k^{2}-\mu ^{2})^{N+2}} . \phantom {\Bigg |} \end{aligned}$$

This is a characteristic of ireg that the UV divergence can be always expressed in terms of a precise set of Basic Divergent Integrals (BDI), composed of scalar and tensorial ones. However, it can be shown that *all* tensorial integrals can be further expressed in terms of the scalar ones plus surface terms. In our particular example one has

4.7 $$\begin{aligned} \Upsilon _{0}^{\mu \nu }&=\int \frac{{d}^4k}{(2 \pi )^4} \frac{\partial }{\partial k_{\mu }}\frac{k^{\nu }}{(k^{2}-\mu ^{2})^{2}} =g^{\mu \nu }I_{\text {log}}(\mu ^{2})\nonumber \\&\quad -4 I_{\text {log}}^{\mu \nu }(\mu ^{2})\equiv g^{\mu \nu }\upsilon _{0,2} , \end{aligned}$$

where $\Upsilon _{0}^{\mu \nu }$ is a surface term, arbitrary in principle. More comments regarding the surface terms and their relation to momentum routing invariance will be given at the end of this section.

After all UV divergences are taken care of, one needs to evaluate the finite part, for which we obtain4.8 It should be noticed that the limit $\mu ^{2} \rightarrow 0$ has still to be taken in the final result. However, it can easily be seen that both $I_{\text {log}}(\mu ^{2})$ and the logarithm term then develop an IR singularity which is spurious since our starting integral was IR safe. To avoid this issue, one still needs to introduce a scale $\lambda ^{2} \ne 0$, which plays the role of a renormalization scale in renormalization-group equations,

4.9 $$\begin{aligned} I_{\text {log}}(\mu ^2) =I_{\text {log}}(\lambda ^2)-b \ln \bigg (\mu ^2/\lambda ^2\bigg ) . \end{aligned}$$

Combining the divergent and finite part and writing the dimension of the external momentum explicitly, one finally gets^10^
4.10In summary, the treatment of UV divergent amplitudes in ireg can be described as follows:

1. Introduce a fictitious mass $\mu ^{2}$ in propagators to avoid spurious IR divergences in the course of the evaluation.
2. Use Eq. (4.4) as many times as necessary to free the divergent part from physical parameters like external momenta and masses. In the case of massive theories, a similar identity can be applied; see Ref. [76] for details.
3. Express the divergent part in terms of scalar and tensorial basic divergent integrals.
4. Reduce tensorial BDIs to the scalar ones plus surface terms.
5. Remove the $\mu ^{2}$ dependence by introducing a scale $\lambda ^{2}$ which plays the role of a renormalization scale on renormalization-group equations.

At this point, we would like to emphasize the role played by the surface terms which, as defined, are just differences between integrals with the same degree of divergence. As shown in Ref. [77], these objects are at the root of momentum routing invariance (the freedom one has in the assignment of internal momenta inside a given Feynman diagram). This can only be respected when the surface terms are set to zero. It can also be shown that the same conclusion holds for Abelian gauge invariance, allowing one to conjecture that surface terms are at the root of symmetry breaking in general. In Ref. [77], it is shown that this conjecture may hold for supersymmetric theories as well. Similar analyses, in many different theories and contexts, have been carried out in Refs. [78–91].

### Application example: $e^{+} e^{-}\rightarrow \gamma ^{*}\rightarrow q\bar{q}$ at NLO

In this section we perform the computation of the total cross section of the process $e^{+}e^{-} \rightarrow \gamma ^{*} \rightarrow q\bar{q}$, showing an example on how ireg deals with different kinds of divergences. We divide the presentation in two parts, as usual.

#### Virtual contributions

The (unregularized) amplitude for the one-loop vertex correction subgraph $\gamma ^{*} \rightarrow q {\bar{q}}$ reads4.11where $p_q$ and $p_{\bar{q}}$ denote the four-momenta of the massless quarks. Using the Dirac equation for massless quarks, the integral can be decomposed as

4.12 $$\begin{aligned} \mathcal{A}^{(1)}_{\mu }&= -4e {Q_{q}}g_s^2 {C_F}\{{\bar{u}}(p_q) \gamma _\mu u(p_{\bar{q}})\nonumber \\&\quad \times [ (p_q\cdot p_{\bar{q}}) I -(p_{q,\alpha } -p_{\bar{q},\alpha }) I^\alpha -I_2/2 ] \nonumber \\&\quad + {\bar{u}}(p_q) \gamma _\alpha u(p_{\bar{q}})\nonumber \\&\quad \times [ (p_{q,\mu } -p_{\bar{q},\mu }) I^\alpha +I^\alpha _{\phantom {\alpha }\mu } ] \} , \end{aligned}$$

with

4.13a $$\begin{aligned} \{I,I^\alpha ,I^{\alpha \beta }\}&=\int \frac{{d}^4 k}{(2 \pi )^4} \frac{\{1,k^\alpha ,k^{\alpha }k^{\beta }\}}{k^2(k+p_q)^2(k-p_{\bar{q}})^2} , \end{aligned}$$

4.13b $$\begin{aligned} I_2&=\int \frac{{d}^4 k}{(2 \pi )^4} \frac{k^2}{k^2(k+p_q)^2(k-p_{\bar{q}})^2}\nonumber \\&= \int \frac{{d}^4 k}{(2 \pi )^4} \frac{1}{(k+p_q)^2(k-p_{\bar{q}})^2} . \end{aligned}$$

One notices the prescription of ireg to cancel denominators as in $I_2$ *before* introducing a regulating mass in the propagators.^11^

The integrals in Eq. (4.13) are IR divergent for $p_q^2 =p_{\bar{q}}^2 =0$. In addition, the integral in Eq. (4.13a) carrying two Dirac indices and the integral in Eq. (4.13b) are logarithmically UV divergent. To deal with the latter, a regulating mass $\mu$ is introduced in all propagators,

4.14 $$\begin{aligned}&\{ I_{{{\textsc {ireg}}}}^{\phantom {\alpha }}, I_{{{\textsc {ireg}}}}^{\alpha }, I_{{{\textsc {ireg}}}}^{\alpha \beta } \}\nonumber \\&\quad =\int \frac{{d}^4 k}{(2\pi )^4} \frac{ \{1,k^\alpha ,k^{\alpha }k^{\beta }\}}{ [k^2-\mu ^2][(k+p_q)^2-\mu ^2][(k-p_{\bar{q}})^2-\mu ^2]} , \end{aligned}$$

and, after cancellation of one of the denominators, also in

4.15 $$\begin{aligned} I_{2,{{\textsc {ireg}}}} =\int \frac{{d}^4 k}{(2 \pi )^4} \frac{1}{[(k+p_q)^2-\mu ^2][(k-p_{\bar{q}})^2-\mu ^2]} . \end{aligned}$$

The limit $\mu ^2 \rightarrow 0$ in the divergent contributions is only to be taken after the cross section of the whole process has been evaluated. Endowed with the regulating mass, all integrals are IR finite. Using $\mu _0 \equiv \mu ^2/s$ and $s \equiv (p_q+p_{\bar{q}})^2 =2 p_q\cdot p_{\bar{q}}$, one obtains^12^

4.16a $$\begin{aligned} I_{{{\textsc {ireg}}}} |_{{p_q^2=p_{\bar{q}}^2=0}}&=\frac{i}{(4\pi )^2}\frac{1}{s}\nonumber \\&\quad \times \bigg [ \frac{\ln ^2(\mu _0)}{2} +i\pi \ln (\mu _0) -\frac{\pi ^2}{2} +\mathcal {O}(\mu _0)\bigg ] ,\end{aligned}$$

4.16b $$\begin{aligned} I_{{{\textsc {ireg}}}}^{\alpha } |_{{p_q^2=p_{\bar{q}}^2=0}}&=\frac{i}{(4\pi )^2}\frac{(p_q-p_{\bar{q}})^{\alpha }}{s} \nonumber \\&\quad \times [\ln (\mu _0)+i\pi +2+ \mathcal {O}(\mu _0) ] , \end{aligned}$$

4.16c $$\begin{aligned} I_{{{\textsc {ireg}}}}^{\alpha \beta } |_{{p_q^2=p_{\bar{q}}^2=0}}&= \frac{g^{\alpha \beta }}{4} \{ I_{\text {log}}(\mu ^2) + \frac{i}{(4\pi )^2} [\ln (\mu _0)+i\pi +3 ]\} \nonumber \\&\quad -\frac{i}{(4\pi )^2}\frac{1}{2s} \{{p_q}^\alpha ( {p_{\bar{q}}}^\beta +{p_q}^\beta [\ln (\mu _0)+i\pi +2 ] )\nonumber \\&\quad +(q,\bar{q}) \rightarrow (\bar{q}, q) \} + \mathcal {O}(\mu _0), \end{aligned}$$

4.16d $$\begin{aligned} I_{2,{{\textsc {ireg}}}} |_{{p_q^2=p_{\bar{q}}^2=0}}&= I_{\text {log}}(\mu ^2) + \frac{i}{(4\pi )^2} [\ln (\mu _0)+i\pi +2 +\mathcal {O}(\mu _0) ]. \end{aligned}$$

In the UV divergent integrals, the BDI $I_{\text {log}}(\mu ^2)$ has been isolated, according to the rules of ireg. Inserting the integrals from Eq. (4.16) into Eq. (4.12) and performing the remaining contractions, one obtains for the one-loop vertex correction

4.17 $$\begin{aligned} (\mathcal{A}^{(1)}_{{{\textsc {ireg}}}})_{\mu }&=(\mathcal{A}^{(0)}_{{{\textsc {ireg}}}})_{\mu } \bigg (\frac{\alpha _s}{\pi }\bigg ) {C_F}\bigg [ -\frac{\ln ^2(\mu _0)}{4} -\frac{3+2i\pi }{4}\nonumber \\&\quad \times \ln (\mu _0) -\frac{7-\pi ^2+3i\pi }{4} +\mathcal {O}(\mu _0) \bigg ] , \end{aligned}$$

where the UV divergent contributions $\sim \! I_{\text {log}}(\mu ^2)$ are dropped. Taking twice the real part of the one-loop correction, the virtual contribution to the total cross section is then given by^13^

4.18 $$\begin{aligned} \sigma ^{(v)}_{{{\textsc {ireg}}}}&=\sigma ^{(0)} \bigg (\frac{\alpha _s}{\pi }\bigg ) {C_F}\bigg [ -\frac{\ln ^2(\mu _0)}{2} -\frac{3}{2} \ln (\mu _0)\nonumber \\&\quad -\frac{7-\pi ^2}{2} +\mathcal {O}(\mu _0) \bigg ] , \end{aligned}$$

with $\sigma ^{(0)}$ given in Eq. (2.15). The divergences occurring in the limit of a vanishing regulator mass $\mu _0$ will be exactly canceled by the cross section related to the bremsstrahlung diagrams, as shown in the next section.

#### Real contributions

In the following we obtain the bremsstrahlung contribution to the total cross section, using the same regulator mass $\mu$ for the gluon and the quarks, as in the previous section. At least at NLO, apart from minor technical differences, the treatment of IR singularities in ireg is equivalent to the fdr solution proposed in Ref. [92] (see also Sect. 5.3).

The total cross section pertaining to the real emission process $e^{+}(p') e^{-}(p)\rightarrow \gamma ^*(q) \rightarrow q(k_1) {\bar{q}}(k_2) g(k_3)$ is obtained:

4.19 $$\begin{aligned} \sigma ^{(r)}_{{{\textsc {ireg}}}}&=\frac{1}{2 s} \int \frac{{d}^3k_1}{(2\pi )^3 2\omega _1} \int \frac{{d}^3k_2}{(2\pi )^3 2\omega _2} \nonumber \\&\quad \times \int \frac{{d}^3k_3}{(2\pi )^3 2\omega _3} (2\pi )^4 \delta ^{(4)}(q-k_1-k_2-k_3)\nonumber \\&\quad \times M^{(0)}_{{{\textsc {ireg}}}}(q\bar{q}g) , \end{aligned}$$

in terms of $k_i^0 =\omega _i=\sqrt{{\vec k}_i^2 +\mu ^2}$.

Let us first analyze how the regulating mass enters the phase-space integration boundaries. Using the CM frame of the virtual photon, $\delta ^{(4)}(q -k_1 -k_2 -k_3)= \delta (q_0 -\omega _1 -\omega _2 -\omega _3)\times$ $\delta ^{(3)}({\vec k}_1 +{\vec k}_2 +{\vec k}_3)$, and after integrating out the three-momentum of the gluon, the phase-space integration *P* reduces to

4.20a $$\begin{aligned} P&=\int \frac{{d}^3k_1}{(2\pi )^3 2\omega _1} \int \frac{{d}^3k_2}{(2\pi )^3 2\omega _2}\nonumber \\&\quad \times \int \frac{{d}^3k_3}{(2\pi )^3 2\omega _3} (2\pi )^4 \delta ^{(4)}(q-k_1-k_2-k_3) , \end{aligned}$$

4.20b $$\begin{aligned}&=\int \frac{{d}^3k_1}{(2\pi )^3 2\omega _1} \int \frac{{d}^3k_2}{(2\pi )^3 2\omega _2} \left( \frac{\pi }{\omega _3}\right) \nonumber \\&\quad \times \delta (q_0-\omega _1-\omega _2-\omega _3) , \end{aligned}$$

with $\omega _3 ={\sqrt{({\vec k}_1 +{\vec k}_2)^2 +\mu ^2}}$. The integration over the angle $\theta$ between ${\vec k}_1$ and ${\vec k}_2$ is performed, noting that $\omega _3 {d}\omega _3=|{\vec k}_1||{\vec k}_2| {d}\text {cos}(\theta )$. In addition, with $|{\vec k}_i| {d}|{\vec k}_i|=\omega _i {d}\omega _i$ we get

4.21 $$\begin{aligned} P&=\frac{1}{32\pi ^3} \int _{\omega _{1m}}^{\omega _{1M}} {d}\omega _1 \int _{\omega _{2m}}^{\omega _{2M}} {d}\omega _2\nonumber \\&\quad \times \int _{\omega _{3m}}^{\omega _{3M}} {d}\omega _3\ \delta ^{(0)}(q_0-\omega _1-\omega _2-\omega _3) . \end{aligned}$$

The boundary values for the $\omega _3$ integration can be traced back from the range of allowed $\theta$ angle values. At fixed ${\vec k}_1$ and ${\vec k}_2$ one thus obtains $\omega _{3m} ={\sqrt{\mu ^2 +(|{\vec k}_1| -|{\vec k}_2|)^2}}$ corresponding to $\theta =\pi$ and $\omega _{3M} ={\sqrt{\mu ^2 +(|{\vec k}_1| +|{\vec k}_2|)^2}}$ for $\theta =0$. In the first case, the quark and antiquark have opposite momenta and thus a soft gluon momentum ${\vec k}_3$ can be emitted together with hard fermion momenta. In the second case, the fermions move parallel and soft gluon emission is accompanied with soft fermion momenta. Introducing now dimensionless variables

4.22 $$\begin{aligned} \chi _i=\frac{(k_i-q)^2}{q^2}-\frac{\mu ^2}{q^2} \end{aligned}$$

with $k_i^2=\mu ^2$ and $q^2=q_0^2$, one gets $\chi _i=1-2 \frac{\omega _i}{q_0}$ and ${d}\chi _i=-2 \frac{{d}\omega _i}{q_0}$. In these variables, the phase-space integral becomes

4.23 $$\begin{aligned} P =\frac{ q_0^2}{(4\pi )^3} \int _{\chi _{1m}}^{\chi _{1M}} {d}\chi _1 \int _{\chi _{2m}}^{\chi _{2M}} {d}\chi _2 , \end{aligned}$$

keeping in mind the interval allowed for non-vanishing contributions of the $\delta$-integration. The latter restrict the boundaries of the $\chi _2$ integration to

4.24 $$\begin{aligned} \chi _2^{\pm }=\frac{1-\chi _1}{2} \pm \sqrt{\frac{(\chi _1-3 \mu _0) [(1-\chi _1)^2-4\mu _0]}{4 (\chi _1+\mu _0)}} , \end{aligned}$$

with the notation $\chi _2^{+}=\chi _{2M}$, $\chi _2^{-}=\chi _{2m}$. Finally, the $\chi _1$ integration boundaries are obtained as follows. From $\chi _1=1-2 \frac{\omega _1}{q_0}$, the upper limit is easily extracted, given when ${\vec k}_1=0$,

4.25a $$\begin{aligned} \chi _{1M} = 1 - 2 \sqrt{\mu _0} . \end{aligned}$$

The lower boundary is obtained for maximal $\omega _1$, i.e. for ${\omega _1}_M=\mu ^2+|{\vec k}_{1M}|^2 =\mu ^2+(|{\vec k}_2|+|{\vec k}_3|)^2$, achieved when the angle ${\theta }_{23}$ between the fermion and the gluon is zero. Using further that energy conservation is expressed in the $\chi$ variables as $1=\chi _1+\chi _2+\chi _3$ and rewriting Eq. (4.22) as $\frac{|{\vec k}_i|^2}{q_0^2} =\frac{(1-\chi _i)^2}{4}-\frac{\mu ^2}{q_0^2}$, one can express ${\omega _1}_M$ only in terms of the variables $\chi _1, \chi _2, \mu _0$. The minimum value of $\chi _1$ then occurs for $\chi _2=\frac{(1-3\mu _0)}{2}$, leading to

4.25b $$\begin{aligned} \chi _{1m} = 3\mu _0 . \end{aligned}$$

Using Eqs. (4.25) and (4.24) together with $q_0^2=q^2=s$, we obtain for the phase-space integral

4.26 $$\begin{aligned} P = \frac{s}{(4\pi )^3} \int _{3\mu _0}^{1-2 \sqrt{\mu _0}} {d}\chi _1 \int _{\chi _{2m}}^{\chi _{2M}} {d}\chi _2 \ \equiv \ \frac{s}{(4\pi )^3} \iint \limits _{\chi _1 \chi _2} . \end{aligned}$$

We now turn back to Eq. (4.19) and evaluate the matrix element squared. Following Sect. 2.3, it can be written as

4.27 $$\begin{aligned} M^{(0)}_{{{\textsc {ireg}}}}(q\bar{q}g) = e^2 g_s^2 \omega ^{(r)} L_{\mu \nu } G^{\mu \nu } , \end{aligned}$$

with 4.28a
4.28b where we use the leptonic tensor of Eq. (2.13) and $\omega ^{(r)}=2 {Q_{q}^{2}}{C_F}/s^2$.

The result can be simplified by considering gauge invariance, which implies that $G^{\mu \nu }$, after phase-space integration, must be transverse to the photon momentum *q*. Thus, the total cross section due to real contribution can be expressed as

4.29 $$\begin{aligned} \sigma _{{{\textsc {ireg}}}}^{(r)} = \sigma ^{(0)} \bigg (\frac{\alpha _{s}}{\pi }\bigg ) {C_F}\iint \limits _{\chi _1 \chi _2} \bigg [-\frac{1}{2} g_{\mu \nu } G^{\mu \nu }\bigg ] . \end{aligned}$$

After a tedious, yet straightforward computation, one obtains

4.30 $$\begin{aligned} -\frac{1}{2} g_{\mu \nu }G^{\mu \nu }= & {} -\left[ \frac{1}{\mu _{0}+\chi _{1}} +\frac{1}{\mu _{0}+\chi _{2}}\right] \nonumber \\&+\,\frac{1}{2}\left[ \frac{\chi _{2}}{\mu _{0}+\chi _{1}} +\frac{\chi _{1}}{\mu _{0}+\chi _{2}}\right] \nonumber \\&+\,\frac{1}{(\mu _{0}+\chi _{1})(\mu _{0}+\chi _{2})} +\mathcal {O}(\mu _{0}) , \end{aligned}$$

where we use the definition of $\chi _{i}$ in Eq. (4.22) and $k_{i}^{2}=\mu ^{2}$. Finally, the integrals can be evaluated with^14^

4.31a $$\begin{aligned} \iint \limits _{\chi _1 \chi _2} \frac{1}{\mu _{0}+\chi _{1}} = \iint \limits _{\chi _1 \chi _2} \frac{1}{\mu _{0}+\chi _{2}}&= -\ln (\mu _{0})-3 + \mathcal {O}(\mu _{0}) , \end{aligned}$$

4.31b $$\begin{aligned} \iint \limits _{\chi _1 \chi _2} \frac{\chi _{2}}{\mu _{0}+\chi _{1}} = \iint \limits _{\chi _1 \chi _2} \frac{\chi _{1}}{\mu _{0}+\chi _{2}}&= -\frac{\ln (\mu _{0})}{2}-\frac{7}{4} + \mathcal {O}(\mu _{0}) ,\end{aligned}$$

4.31c $$\begin{aligned} \iint \limits _{\chi _1 \chi _2} \frac{1}{(\mu _{0}+\chi _{1})(\mu _{0}+\chi _{2})}&= \frac{\ln ^{2}(\mu _{0})}{2}-\frac{\pi ^{2}}{2} + \mathcal {O}(\mu _{0}). \end{aligned}$$

Finally, the total cross section due to the real contribution is given by^15^

4.32 $$\begin{aligned} \sigma _{{{\textsc {ireg}}}}^{(r)}&= \sigma ^{(0)}\left( \frac{\alpha _{s}}{\pi }\right) {C_F}\nonumber \\&\quad \times \bigg [ \frac{\ln ^{2}(\mu _{0})}{2} +\frac{3}{2}\ln (\mu _0) +\frac{17}{4} -\frac{\pi ^{2}}{2} + \mathcal {O}(\mu _0) \bigg ] . \end{aligned}$$

The procedure of obtaining the real corrections in ireg can be summarized as follows: compute the matrix element squared for *massless* external and internal particles as in Eq. (4.28b). However, the on-shell limit $k_i^2=0$ should not be applied. Instead, wherever a squared momentum appears it should be replaced by $k_i^2=\mu ^2$. The phase-space integration is to be carried out for massive external particles. IR divergences appear as $\ln (\mu _0)$ terms.^16^

Finally, adding the virtual contribution, Eq. (4.18), one obtains the well-known UV and IR finite result

4.33 $$\begin{aligned} \sigma ^{(1)}= & {} \sigma ^{(0)} + \sigma ^{(v)}_{{{\textsc {ireg}}}} + \sigma ^{(r)}_{{{\textsc {ireg}}}} |_{\mu _0\rightarrow 0} = \frac{{Q_{q}^{2}}N_c}{3 s}\bigg (\frac{e^4}{4\pi }\bigg ) \nonumber \\&\times \bigg [ 1+\bigg (\frac{\alpha _s}{4\pi }\bigg ) 3 {C_F}\bigg ] . \end{aligned}$$

### Established properties of IREG

#### Gauge invariance

In gauge theories, the initial structure of a given Feynman diagram contains Dirac matrices, Lorentz contractions, etc. These operations may generate terms with squared momenta in the numerator which must be canceled against propagators *before* applying the rules of ireg. This point was first emphasized in differential regularization whose rules have a one-to-one correspondence with the ireg prescription [78]. As an example, consider the (unregularized) off-shell vacuum polarization tensor in massless QED at one loop4.34which, after evaluating the Dirac algebra, can be expressed as

4.35a $$\begin{aligned} \Pi ^{\mu \nu }&=-4e^{2}\int \frac{{d}^4k}{(2 \pi )^4}\frac{ 2k^{\mu }k^{\nu } -g^{\mu \nu }k^{2} -k^{\mu }p^{\nu }-k^{\nu }p^{\mu } +g^{\mu \nu }(k\cdot p) }{k^{2}(k-p)^{2}}, \end{aligned}$$

4.35b $$\begin{aligned}&\equiv -4e^{2}\big [ 2I^{\mu \nu } -g^{\mu \nu }J -I^{\mu }p^{\nu }-I^{\nu }p^{\mu } +g^{\mu \nu }(I_{\alpha }p^{\alpha }) \big ]. \end{aligned}$$

The integrals, after applying the rules of ireg, are given as

4.36a $$\begin{aligned} J_{{{\textsc {ireg}}}}&= \int \frac{{d}^4k}{(2 \pi )^4}\frac{k^2}{k^{2}(k-p)^{2}}\nonumber \\&= \int \frac{{d}^4k}{(2 \pi )^4}\frac{1}{(k-p)^{2}} =-p^{2}\upsilon _{0,2} , \end{aligned}$$

4.36b $$\begin{aligned} I_{{{\textsc {ireg}}}}^{\mu }&= \int \frac{{d}^4k}{(2 \pi )^4}\frac{k^{\mu }}{k^{2}(k-p)^{2}}\nonumber \\&= \frac{p^{\mu }}{2}\left[ I_{\text {log}}(\lambda ^{2}) -b\ln \left( -\frac{p^{2}}{\lambda ^{2}}\right) +2 b -\upsilon _{0,2} \right] , \end{aligned}$$

4.36c $$\begin{aligned} I_{{{\textsc {ireg}}}}^{\mu \nu }&= \int \frac{{d}^4k}{(2 \pi )^4}\frac{k^{\mu }k^{\nu }}{k^{2}(k-p)^{2}}\nonumber \\&= \frac{1}{3}p^{\mu }p^{\nu }\left[ I_{\text {log}}(\lambda ^{2}) -b\ln \left( -\frac{p^{2}}{\lambda ^{2}}\right) +\frac{11}{6}b \right] \nonumber \\&\quad -\frac{1}{12}g^{\mu \nu }p^{2}\left[ I_{\text {log}}(\lambda ^{2}) -b\ln \left( -\frac{p^{2}}{\lambda ^{2}}\right) +\frac{4}{3}b \right] \nonumber \\&\quad -\frac{g^{\mu \nu }}{2}\upsilon _{2,2} -\frac{1}{6}(g^{\mu \nu }p^{2}+2p^{\mu }p^{\nu })\upsilon _{0,4}\nonumber \\&\quad +\frac{1}{4}g^{\mu \nu }p^{2}\upsilon _{0,2} , \end{aligned}$$

where we have suppressed quadratic divergences (in the example they cancel exactly), and $\nu _{i,j}$ are surface terms defined as

4.37 $$\begin{aligned} g^{\{\nu _{1}\cdots \nu _{j}\}}\upsilon _{i,j} \equiv \Upsilon _{i}^{\nu _{1}\cdots \nu _{j}} \equiv \int {\frac{{d}^{4}k}{(2 \pi )^4}} \frac{\partial }{\partial k_{\nu _{1}}}\frac{k^{\nu _{2}}\cdots k^{\nu _{j}}}{(k^{2}-\mu ^{2})^{\frac{2+j-i}{2}}} , \end{aligned}$$

where we use $g^{\{\nu _{1}\cdots \nu _{j}\}} \equiv g^{\nu _{1}\nu {2}}\cdots g^{\nu _{j-1}\nu _{j}}$ + symmetric combinations. Inserting all results in $\Pi _{}^{\mu \nu }$, one obtains

4.38 $$\begin{aligned} \Pi _{{{\textsc {ireg}}}}^{\mu \nu }&=-\frac{4}{3}e^{2} ( g^{\mu \nu }p^{2} -p^{\mu }p^{\nu } )\nonumber \\&\quad \times \left[ I_{\text {log}}(\lambda ^{2}) -b\ln \left( -\frac{p^{2}}{\lambda ^{2}}\right) +\frac{7}{3}b \right] \nonumber \\&\quad -4 e^{2}\bigg [ -\frac{1}{3}(g^{\mu \nu }p^{2}+2p^{\mu }p^{\nu })\upsilon _{0,4} \nonumber \\&\quad +p^{\mu }p^{\nu }\upsilon _{0,2} -g^{\mu \nu }\upsilon _{2,2} \bigg ] . \end{aligned}$$

As can be seen, to enforce gauge invariance (expressed in the transversality of $\Pi _{{{\textsc {ireg}}}}^{\mu \nu }$), surface terms should be null as previously discussed [77].

We remark the appearance of a $k^{2}$ term in Eq. (4.35), defined as the divergent *J* integral, and the importance of applying ireg rules only *after* cancelling such term against propagators. Proceeding otherwise, by rewriting $k^2=g^{\mu \nu }k_{\mu }k_{\nu }$ for instance, one would obtain

4.39 $$\begin{aligned}&\int \frac{{d}^4k}{(2 \pi )^4}\frac{k^2}{k^{2}(k-p)^{2}} = g^{\mu \nu }\int \frac{{d}^4k}{(2 \pi )^4}\frac{k_{\mu }k_{\nu }}{k^{2}(k-p)^{2}}\nonumber \\&\quad = \frac{p^{2}}{6}b -2 \upsilon _{2,2} - p^{2}(\upsilon _{0,4}-\upsilon _{0,2}) , \end{aligned}$$

which is different from the *J* integral, Eq. (4.36a), not only by arbitrary terms encoded in the $\upsilon _{i,j}$ but also by a finite term. In this way, gauge invariance would be broken even if the surface terms are systematically set to zero. It should be emphasized that the discussion above is restricted to divergent integrals.

#### UV renormalization

We would also like to briefly show how renormalization-group functions can be computed in the framework of ireg. For simplicity, we adopt the background field method [93] which relates the wave function renormalization of the background field, $B_{0}=Z_{B}B$, with the coupling renormalization, $e_{0}=Z_{e} e$, through the equation $Z_{e}=Z_{B}^{-1/2}$. Therefore, by applying this method to QED, the $\beta$ function can be obtained only with the knowledge of the vacuum polarization tensor. Performing a minimal subtraction, which in ireg amounts to subtract only basic divergent integrals as $I_{\text {log}}(\lambda ^{2})$, and remembering that $\lambda$ plays the role of a renormalization-group scale, one obtains^17^

4.40 $$\begin{aligned} \beta&=\lambda ^2\frac{\partial }{\partial \lambda ^2}\bigg (\frac{e}{4\pi }\bigg )^2 =\frac{e^4}{(4\pi )^2} \frac{4}{3}N_F i \lambda ^2\frac{\partial }{\partial \lambda ^2} I_{\text {log}}(\lambda ^{2}) +\mathcal {O}(e^6)\nonumber \\&=-\bigg (\frac{e}{4\pi }\bigg )^4\bigg [-\frac{4}{3}N_F\bigg ] +\mathcal {O}(e^{6}) . \end{aligned}$$

Further examples can be found in Refs. [79, 81, 89, 94, 95].

## FDR: four-dimensional regularization/renormalization

fdr [96] is a fully four-dimensional framework to compute radiative corrections in QFT. The calculation of the loop corrections is conceptually simplified with respect to more traditional approaches in that there is no need to include UV counterterms in the Lagrangian $\mathcal {L}$. In fact, the outcome of an fdr calculation at any loop order is directly a UV-renormalized quantity. Moreover, this particular way of looking at the UV problem may open new perspectives in the present understanding of fundamental and effective QFTs [97]. In the following, we review the fdr treatment of UV and IR divergences, also using the $e^{+} e^{-}\rightarrow \gamma ^{*}\rightarrow q\bar{q}(g)$ process as an explicit example.

### FDR and UV infinities

Let $J(q_1,\ldots ,q_\ell )$ be an integrand depending on $\ell$ integration momenta $q_1,\ldots ,q_\ell$. The fdr integral over *J* is *defined* as follows:

5.1 $$\begin{aligned}&\int [d^4 q_1] \cdots [d^4 q_\ell ] J(q_1,\ldots ,q_\ell ,\mu ^2)\nonumber \\&\quad \equiv \lim _{\mu \rightarrow 0} \int d^4 q_1 \cdots d^4 q_\ell J_\mathrm{F}(q_1,\ldots ,q_\ell ,\mu ^2) , \end{aligned}$$

where $J_\mathrm{F}(q_1,\ldots ,q_\ell ,\mu ^2)$ is the UV-finite part of $J(q_1,\ldots ,q_\ell ,\mu ^2)$ (specified below), $\mu$ is an infinitesimal mass needed to extract $J_\mathrm{F}$ from *J*, and $\int [d^4q_i]$ denotes the fdr integration. The integrands $J(q_1,\ldots ,q_\ell ,\mu ^2)$ and $J_\mathrm{F}(q_1,\ldots ,q_\ell ,\mu ^2)$ are obtained from $J(q_1,\ldots ,q_\ell )$ with the help of the following rules:

(i) Squares of integration momenta appearing both in the denominators of $J(q_1,\ldots ,q_\ell )$ and in contractions generated in the numerator by Feynman rules are shifted by $\mu ^2$,
  5.2 $$\begin{aligned} q^2_i \rightarrow q^2_i -\mu ^2 \equiv \bar{q}^2_i . \end{aligned}$$
  This replacement is called *global prescription*.
(ii) A splitting
  5.3 $$\begin{aligned} J(q_1,\ldots ,q_\ell ,\mu ^2)&= [J_\mathrm{INF}(q_1,\ldots ,q_\ell ,\mu ^2)]\nonumber \\&\quad +J_\mathrm{F}(q_1,\ldots ,q_\ell ,\mu ^2) \end{aligned}$$
  is performed in such a way that UV divergences are entirely parametrized in terms of divergent integrands contained in $[J_\mathrm{INF}]$, which solely depend on $\mu ^2$. By convention, we write divergent integrands in square brackets and call them fdr *vacua*, or simply *vacua*.
(iii) The global prescription in Eq. (5.2) should be made compatible with a key property of multi-loop calculus:
  5.4 $$\begin{aligned}&\text {In an } \ell \text {-loop diagram, one should be able to}\nonumber \\&\quad \text {calculate a subdiagram}, \nonumber \\&\text {insert the integrated form into the full diagram}\nonumber \\&\quad \text { and get the same answer.} \end{aligned}$$
  We dub this *subintegration consistency*.

Finally, after $\lim _{\mu \rightarrow 0}$ is taken, $\ln \mu \rightarrow \ln \mu _{\mathrm{R}}$ is understood on the r.h.s. of Eq. (5.1), where $\mu _{\mathrm{R}}$ is an arbitrary renormalization scale. Note that inserting Eq. (5.3) into Eq. (5.1) gives an alternative definition

5.5 $$\begin{aligned}&\int [d^4 q_1] \cdots [d^4 q_\ell ] J(q_1,\ldots ,q_\ell ,\mu ^2) \nonumber \\&\quad = \lim _{\mu \rightarrow 0} \int _{{{\textsc {r}}}} d^4 q_1 \cdots d^4 q_\ell \{ J(q_1,\ldots ,q_\ell ,\mu ^2)\nonumber \\&\qquad -[J_\mathrm{INF}(q_1,\ldots ,q_\ell ,\mu ^2)] \} , \end{aligned}$$

where r denotes an arbitrary UV regulator. Equation (5.5) tells us that the UV subtraction is directly encoded in the definition of fdr loop integration: no divergent integrand is considered separately from its subtraction term.

fdr integration preserves shift invariance which is easy to prove when using Eq. (5.5) with r = ds,

5.6 $$\begin{aligned}&\int [d^4q_1] \ldots [d^4q_\ell ] J(q_1,\ldots , q_\ell ,\mu ^2)\nonumber \\&\quad =\int [d^4q_1] \ldots [d^4q_\ell ] J(q_1+p_1,\ldots , q_\ell +p_\ell ,\mu ^2) , \end{aligned}$$

and the possibility of cancelling numerators and denominators

5.7 $$\begin{aligned}&\int [d^4q_1] \cdots [d^4q_\ell ] \frac{\bar{q}^2_i-m^2_i}{ (\bar{q}^2_i-m^2_i)^m \cdots } \nonumber \\&\quad = \int [d^4q_1] \cdots [d^4q_\ell ] \frac{1}{ (\bar{q}^2_i-m^2_i)^{m-1}\cdots } , \end{aligned}$$

which are properties needed to retain the symmetries of $\mathcal {L}$ [98]. From Eqs. (5.6) and (5.7) it follows that algebraic manipulations in fdr integrands are allowed as if they where convergent ones. This authorizes one to reduce complicated multi-loop integrals to a limited set of Master integrals (MI) by using four-dimensional tensor decomposition [99] or integration-by-parts identities [100]. In other words, the definition in Eq. (5.1) [or Eq. (5.5)] can be applied just at the end of the calculation, when the actual value of the MIs is needed.

An important subtlety implied by Eq. (5.7) is that the needed cancellation works only if integrands involving explicit powers of $\mu ^2$ in the numerator are also subtracted *as if* $\mu ^2 = q^2_i$, where $q^2_i$ is the momentum squared which generates $\mu ^2$. For instance, one computes

5.8 $$\begin{aligned}&\int [d^4q] \frac{\mu ^2}{(q^2-M^2)^3}\nonumber \\&\quad = \lim _{\mu \rightarrow 0} \mu ^2 \int d^4q \bigg \{ \frac{1}{(q^2-M^2)^3}-\bigg [\frac{1}{\bar{q}^6}\bigg ]\bigg \} = \frac{i\pi ^2}{2} , \end{aligned}$$

in accordance with Eq. (5.5). In this case both integrals on the r.h.s. are UV convergent and the only contribution which survives the ${\mu \rightarrow 0}$ limit is generated by the subtraction term. As a consequence, although only one kind of $\mu ^2$ exists, one has to keep track of its origin when it appears in the numerator of $J(q_1,\ldots ,q_\ell ,\mu ^2)$. For this purpose we use the notation $\mu ^2|_i$, which understands the same subtraction required for the case $\mu ^2= q^2_i$. fdr integrals with powers of $\mu ^2|_i$ in the numerator are called ‘extra integrals’.^18^ Their computation is elementary, as illustrated by Eq. (5.8). Additional one- and two-loop examples can be found in Refs. [96, 99]. fdr extra integrals play an important role in maintaining the theory unitary without the need of introducing counterterms in $\mathcal{L}$, as will be discussed in Sect. 5.4.

As a simple example of an fdr integration, we consider the scalar one-loop integrand

5.9 $$\begin{aligned} J(q)= \frac{1}{(q^2-M^2)^2} , \end{aligned}$$

which diverges logarithmically for $q\rightarrow \infty$. The steps to define its fdr integral are as follows:

- Shift squares of the integration momentum,
  5.10 $$\begin{aligned}&J(q) \rightarrow J(q,\mu ^2) \nonumber \\&\quad \equiv \frac{1}{(\bar{q}^2-M^2)^2} , \quad \mathrm{with}\quad \bar{q}^2 \equiv q^2-\mu ^2 . \end{aligned}$$
- Subtract the divergent part of the integrand $[J_\mathrm{INF}(q,\mu ^2)] = \left[ \frac{1}{\bar{q}^4} \right]$ in the $\mu \rightarrow 0$ limit, setting $\mu \rightarrow \mu _{\mathrm{R}}$ in the logarithms
  5.11 $$\begin{aligned}&\int [d^4q] \frac{1}{(\bar{q}^2-M^2)^2} \nonumber \\&\quad \equiv \lim _{\mu \rightarrow 0} \int _{{{\textsc {r}}}} d^4q \bigg \{ \frac{1}{(\bar{q}^2-M^2)^2} -\bigg [\frac{1}{\bar{q}^4}\bigg ] \bigg \} \Bigg |_{\mu \rightarrow \mu _{\mathrm{R}}} . \end{aligned}$$
- The dependence on r is eliminated by using the partial fraction identity
  5.12 $$\begin{aligned} \frac{1}{\bar{q}^2-M^2} = \frac{1}{\bar{q}^2} \bigg (1+ \frac{M^2}{\bar{q}^2-M^2}\bigg ) \end{aligned}$$
  in the first integrand on the r.h.s. of Eq. (5.11). This exactly cancels the divergent term $\big [\frac{1}{\bar{q}^4}\big ]$ *before* integration, leaving the UV-finite result^19^
  5.13a $$\begin{aligned}&\int [d^4q] \frac{1}{(\bar{q}^2-M^2)^2} \equiv \lim _{\mu \rightarrow 0}\nonumber \\&\qquad \times \int d^4q\bigg \{ \frac{M^2}{\bar{q}^4(\bar{q}^2-M^2)} +\frac{M^2}{\bar{q}^2(\bar{q}^2-M^2)^2}\bigg \} \Bigg |_{\mu \rightarrow \mu _{\mathrm{R}}}\end{aligned}$$
  5.13b $$\begin{aligned}&\quad = -i \pi ^2 \ln \frac{M^2}{\mu _{\mathrm{R}}^2} . \end{aligned}$$

In practice, one can directly start from the integrand in Eq. (5.10) and expand it by means of Eq. (5.12). This procedure allows one to naturally separate $[J_\mathrm{INF}(q_1,\ldots ,q_\ell ,\mu ^2)]$ from any integrand $J(q_1,\ldots ,q_\ell ,\mu ^2)$ and write down definitions analogous to Eq. (5.13) at any loop order. Explicit examples for the extraction of fdr vacua from two-loop integrands are presented in Ref. [99].

Given the fact that the definition of fdr loop integration is compatible with a graphical proof of the Slavnov–Taylor identities through Eqs. (5.6) and (5.7) and can be made congruent with the subintegration consistency of Eq. (5.4) without the need of introducing UV counterterms in $\mathcal{L}$ (see Sect. 5.4 and Ref. [101] for more details on this point), fdr quantities are directly interpretable as UV-renormalized ones. As an example, the correspondence between off-shell two-loop QCD correlators computed in fdr and ds has been worked out in Ref. [101].

**Fig. 9:**
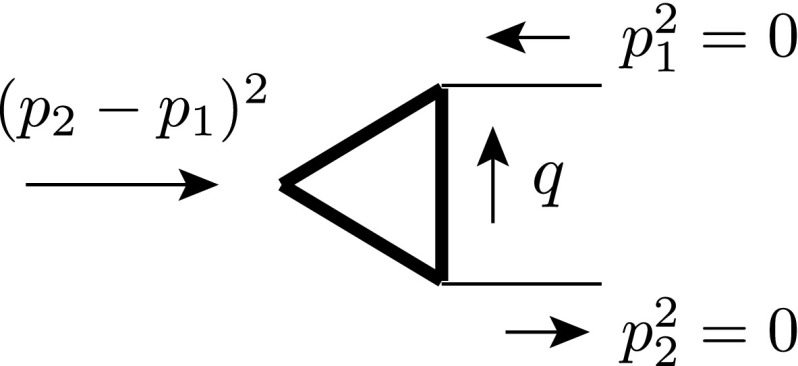
Massless scalar one-loop three-point function. *Thick internal lines* denote the insertion of the infinitesimal mass $\mu$, which generates $\mu$-massive propagators

### FDR and IR infinities

The modification of the propagators induced by Eq. (5.2) also regularizes soft and collinear divergences in the virtual integrals [92]. As an example, the massless one-loop three-point function corresponding to the Feynman diagram shown in Fig. 9 is interpreted in fdr as

5.14a $$\begin{aligned} I_{{{\textsc {fdr}}}} =\int [d^4q]\frac{1}{\bar{q}^2 \bar{D}_1 \bar{D}_2} =\lim _{\mu \rightarrow 0} \int d^4q \frac{1}{\bar{q}^2 \bar{D}_1 \bar{D}_2} , \end{aligned}$$

with $\bar{q}^2= q^2 -\mu ^2$ and $\bar{D}_i= (q+ p_i)^2-\mu ^2$. It is worth noticing that this is the same definition as given in Eq. (5.5). In fact, there is no $[J_\mathrm{INF}]$ term to subtract in this case since the integrand is UV finite. It is easy to compute

5.14b $$\begin{aligned} I_{{{\textsc {fdr}}}} = \frac{i \pi ^2}{s}\bigg [ \frac{\ln ^2(\mu _0)}{2} +i \pi \ln (\mu _0) -\frac{\pi ^2}{2} +\mathcal {O}(\mu _0) \bigg ] , \end{aligned}$$

with $s=(p_2-p_1)^2$ and $\mu _0=\mu ^2/s$. Thus, IR divergences take the form of logarithms of $\mu _0$. In the case at hand, the squared logarithm is generated by an overlap of soft and collinear divergences when $q\rightarrow 0$ and *q* is collinear to $p_i$.

This prescription certainly allows one to assign a precise meaning to virtual integrals also in the presence of IR singularities. Nevertheless, the correct final result is obtained only if the real part of the radiative corrections is treated likewise. This is obtained by carefully analyzing the Cutkowsky rules [102] relating real and virtual contributions with different cuts of diagrams at a higher perturbative level, where cutting a propagator means going on-shell, $\frac{i}{q^2+ i 0} \rightarrow (2\pi ) \delta (q^2) \theta (q_0)$. This correspondence is linked to the identity^20^

5.15 $$\begin{aligned} \frac{i}{q^2+ i 0} = (2\pi ) \delta (q^2) \theta (q_0) + \frac{i}{q^2- i 0 q_0} . \end{aligned}$$

In fact, IR singularities on the l.h.s. of Eq. (5.15) manifest themselves as pinches of the integration path by two (or more) singularities in the $q_0$ complex plane, which occur in the virtual part of the radiative corrections. On the other hand, the first term on the r.h.s. gives end-point singularities, typical of the real radiation, and the last term generates IR finite contributions. It is then clear that the fdr modification $\frac{i}{q^2+ i 0} \rightarrow \frac{i}{\bar{q}^2+ i 0}$ in the virtual contribution is matched by the $\mu$-massive version of Eq. (5.15), namely

**Fig. 10:**
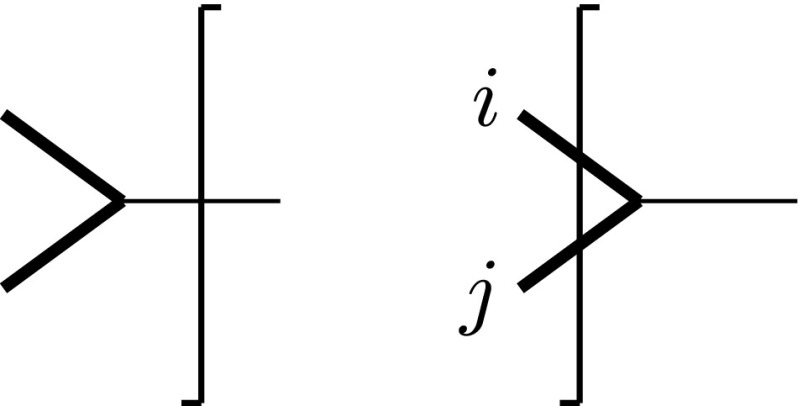
Splitting regularized by $\mu$-massive (*thick*) unobserved particles. The one-particle cut contributes to the virtual part, the two-particle cut to the real radiation

5.16 $$\begin{aligned} \frac{i}{\bar{q}^2+ i 0} =(2\pi ) \delta (\bar{q}^2) \theta (q_0) + \frac{i}{\bar{q}^2- i 0 q_0} , \end{aligned}$$

which in turn is responsible for the correspondence $\frac{i}{\bar{q}^2+ i 0} \rightarrow (2\pi ) \delta (\bar{q}^2) \theta (q_0)$ depicted in Fig. 10; see also Ref. [92]. For example, Eq. (5.16) can be used to rewrite the real part of Eq. (5.14a) as an integral over an eikonal factor

5.17 $$\begin{aligned} \frac{\pi ^2}{4} {\text {Re}}\left( \frac{1}{i \pi ^2}\int [d^4q] \frac{1}{\bar{q}^2 \bar{D}_1 \bar{D}_2} \right) = \lim _{\mu \rightarrow 0} \int \limits _{\bar{\Phi }_3} \frac{1}{\bar{s}_{13} \bar{s}_{23}} , \end{aligned}$$

where $\bar{s}_{ij}=(\bar{p}_i+\bar{p}_j)^2$, $\bar{p}^2_{i,j}=\mu ^2\rightarrow 0$ and $\bar{\Phi }_3$ denotes the $\mu$-massive 3-particle phase space.

**Fig. 11:**
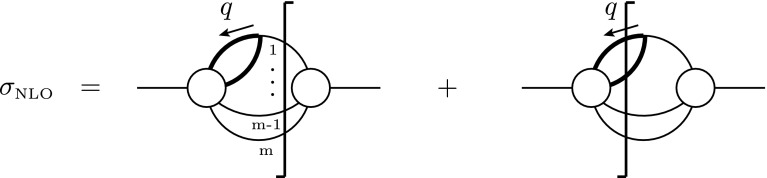
IR divergences drop out when summing the *m*-particle virtual piece $\sigma ^{(v)}_{}$ and its real $(m + 1)$-particle counterpart $\sigma ^{(r)}_{}$. Adding the contributions gives the fully inclusive NLO cross section

In summary, the IR divergent $1\rightarrow 2$ massless splitting gets regularized by the introduction of an infinitesimal mass $\mu$ for all unobserved particles. In the case of external particles, this is equivalent to trade a massless phase space for a $\mu$-massive one. Furthermore, IR infinities cancel when summing real and virtual contributions, for instance

5.18 $$\begin{aligned} \sigma _{} =\int \limits _{\Phi _{m}} d \sigma ^{(v)} +\lim _{\mu \rightarrow 0} \int \limits _{\bar{\Phi }_{m+1}} d \sigma ^{(r)}(\{\bar{s}_{ij}\}) =\sigma ^{(v)}_{} +\sigma ^{(r)}_{} , \end{aligned}$$

as illustrated in Fig. 11. Finally, when $d \sigma ^{(r)}_{}(\{s_{ij}\})$ is analytically known in terms of massless invariants $s_{ij}=(p_i+p_j)^2$ with $p^2_{i,j}=0$, Eq. (5.18) prescribes the replacement $s_{ij}\rightarrow \bar{s}_{ij}$. If, instead, $d \sigma ^{(r)}_{}$ is known only numerically, one can construct a mapping from a massive to a massless phase space, ${\bar{\Phi }}_{m+1} \mathop \rightarrow \limits ^{\mathrm{mapping}}\Phi _{m+1}$, use $\Phi _{m+1}$ to compute massless invariants and rewrite the real contribution as

5.19 $$\begin{aligned} \sigma ^{(r)}_{} = \lim _{\mu \rightarrow 0} \int \limits _{\bar{\Phi }_{m+1}} d \sigma ^{(r)}_{}(\{s_{ij}\})~ \prod _{i < j} \frac{s_{ij}}{\bar{s}_{ij}} . \end{aligned}$$

In this way, $d \sigma ^{(r)}_{}(\{s_{ij}\})$ is gauge invariant, since it is computed with massless kinematics and the fudge factor $\prod _{i < j} \frac{s_{ij}}{\bar{s}_{ij}}$ effectively replaces $s_{ij}\rightarrow {\bar{s}}_{ij}$ in all relevant IR singular configurations. This is because $d \sigma ^{(r)}_{}(\{s_{ij}\}) \sim {\frac{1}{s_{ij}}}$ when $s_{ij} \rightarrow 0$.

### Application example: $e^{+} e^{-}\rightarrow \gamma ^{*}\rightarrow q{\bar{q}}$ at NLO

#### Virtual contributions

In this section we perform the computation of the total cross section of the process $e^{+} e^{-}\rightarrow \gamma ^{*}\rightarrow q\bar{q}$ in QCD to illustrate a typical fdr calculation. As for the virtual part of the corrections, scaleless integrals vanish. More precisely, in fdr they are proportional to $\ln \mu _{\mathrm{R}}/\mu$ (where $\mu$ is the IR regulator), which gives zero when choosing $\mu _{\mathrm{R}}=\mu$ [99]. Thus, only the vertex diagram where a virtual gluon connects the quark with the antiquark has to be considered. The only subtle point of the calculation is the replacement

5.20 $$\begin{aligned} / q \gamma ^\alpha / q = - q^2\gamma ^\alpha +2 \gamma _\beta q^\alpha q^\beta \rightarrow -\bar{q}^2\gamma ^\alpha +2 \gamma _\beta q^\alpha q^\beta \end{aligned}$$

in the fermion string, dictated by the global prescription. Note that this is fully equivalent to the ireg recipe of performing simplifications *before* introducing $\mu ^2$ in the denominators. In fact, the replacement in Eq. (5.20) produces a contribution proportional to

5.21 $$\begin{aligned}&\int [d^4q] \frac{-\bar{q}^2\gamma ^\alpha +2 \gamma _\beta q^\alpha q^\beta }{ \bar{q}^2 \bar{D}_1 \bar{D}_2} =-\gamma ^\alpha \int [d^4q] \frac{1}{\bar{D}_1 \bar{D}_2}\nonumber \\&\quad +2 \gamma _\beta \int [d^4q] \frac{q^\alpha q^\beta }{\bar{q}^2 \bar{D}_1 \bar{D}_2} , \end{aligned}$$

which is the same result one would obtain by simplifying before introducing $\mu$-massive propagators. In both cases, the gauge-preserving simplification between the numerator and the denominator of the first integral on the r.h.s. of Eq. (5.21) is achieved. Differences between fdr and ireg start when evaluating the second integral. A customary Passarino–Veltman tensor decomposition is possible in fdr *before* using the definition of the fdr integral given in Eq. (5.1):^21^

5.22 $$\begin{aligned} C^{\alpha \beta }&\equiv \int [d^4q] \frac{q^\alpha q^\beta }{\bar{q}^2 \bar{D}_1 \bar{D}_2} = C_{00} (g^{\alpha \beta }) + C_{11} (p_1^\alpha p_1^\beta )\nonumber \\&+ C_{22} (p_2^\alpha p_2^\beta ) + C_{12} (p_1^\alpha p_2^\beta +p_2^\alpha p_1^\beta ) . \end{aligned}$$

To obtain the coefficients $C_{ij}$, one needs to contract $C^{\alpha \beta }$ with $g_{\alpha \beta }$, resulting in

5.23 $$\begin{aligned} C^{\alpha }_{\phantom {\alpha }\alpha } = \int [d^4q] \frac{q^2}{\bar{q}^2 \bar{D}_1 \bar{D}_2} . \end{aligned}$$

Since $q^2$ in the numerator is *not* generated by Feynman rules, now it would be incorrect to simplify it with the $\bar{q}^2$ denominator, in the sense that one would not obtain the correct value of $C^{\alpha \beta }$. Here is the place where the fdr ’extra integrals’ play an active role. In fact, by adding and subtracting $\mu ^2$, one rewrites

5.24 $$\begin{aligned} C^{\alpha }_{\phantom {\alpha }\alpha }&= \int [d^4q] \frac{\bar{q}^2 + \mu ^2}{\bar{q}^2 \bar{D}_1 \bar{D}_2}= \int [d^4q] \frac{1}{\bar{D}_1 \bar{D}_2}\nonumber \\&\quad +\int [d^4q] \frac{\mu ^2}{\bar{q}^2 \bar{D}_1 \bar{D}_2} , \end{aligned}$$

which produces the correct answer in terms of a minimum set of scalar MIs. In other words, thanks to the introduction of extra integrals, Eq. (5.1) can be considered as a convenient way to define a loop integration for divergent integrals that survives algebraic four-dimensional manipulations. This is a peculiar property of fdr.

In the computation at hand, only $C_{00}$ and $C_{12}$ are needed. The reduction gives

5.25 $$\begin{aligned} C_{00} = \frac{I_{2,{{\textsc {fdr}}}}}{4} + \frac{EI}{2} ,\quad C_{12} = \frac{EI}{s}, \end{aligned}$$

with^22^

5.26a $$\begin{aligned} I_{2,{{\textsc {fdr}}}}&= \int [d^4q] \frac{1}{\bar{D}_1 \bar{D}_2} = \int _0^1 dx \int [d^4q] \nonumber \\&\quad \times \frac{1}{[\bar{q}^2+s x (1-x)+i 0]^2}\nonumber \\&= -\pi ^2 \bigg (\ln \frac{-s-i 0}{\mu _{\mathrm{R}}^2}-2\bigg ) , \end{aligned}$$

5.26b $$\begin{aligned} EI&= \int [d^4q] \frac{\mu ^2}{\bar{q}^2 \bar{D}_1 \bar{D}_2} = \frac{i \pi ^2}{2} ; \end{aligned}$$

see Eqs. (5.13) and (5.8). Analogously, one reduces the rank-one tensor

5.27 $$\begin{aligned} C^{\alpha } \equiv \int [d^4q] \frac{q^\alpha }{\bar{q}^2 \bar{D}_1 \bar{D}_2}= C_{1}^{\phantom {\alpha }} p_1^\alpha + C_{2}^{\phantom {\alpha }} p_2^\alpha , \end{aligned}$$

obtaining

5.28 $$\begin{aligned} C_{1} = C_{2}= \frac{I_{2,{{\textsc {fdr}}}}}{s}. \end{aligned}$$

In summary, the virtual amplitude can be expressed as a linear combination of the scalar integrals in Eqs. (5.14b) and (5.26). Multiplying with the Born amplitude and taking the real part, one obtains^23^

5.29 $$\begin{aligned} \sigma ^{(v)}_{{{\textsc {fdr}}}}&=\sigma ^{(0)} \bigg (\frac{\alpha _s}{\pi }\bigg ) {C_F}\bigg [-\frac{\ln ^2(\mu _0)}{2} -\frac{3}{2} \ln (\mu _0)\nonumber \\&\quad -\frac{7-\pi ^2}{2}+\mathcal {O}(\mu _0) \bigg ] , \end{aligned}$$

where $\sigma ^{(0)}$ is the Born total cross section given in Eq. (2.15) and $\ln \mu _0$ is the IR logarithm. The process at hand is UV finite, so that the dependence on the logarithms has to drop in the final result. As a consequence, the effect of all scaleless integrals (nullified by our particular choice $\mu _{\mathrm{R}}=\mu$) is nothing but $\ln s/\mu _{\mathrm{R}}^2 \rightarrow \ln s/\mu ^2$ in Eq. (5.26a), as can easily be checked with an explicit calculation.

#### Real contributions

As for the bremsstrahlung contribution $e^+e^-\rightarrow \gamma ^{*}\rightarrow q(p_1) \bar{q}(p_2) g(p_3)$, a tensor decomposition of the three-particle phase-space integrals produces the matrix element squared^24^

5.30 $$\begin{aligned}&M_{{{\textsc {fdr}}}}^{(0)}(s_{12},s_{13},s_{23}) = \frac{16 \pi \alpha _s}{s} {C_F}M^{(0)}_{{{\textsc {fdr}}}}(s) \bigg ( -\frac{s}{ s_{13}} -\nonumber \\&\quad \times \frac{s}{ s_{23}} +\frac{ s_{13}}{2 s_{23}} +\frac{ s_{23}}{2 s_{13}} +\frac{s^2}{s_{13} s_{23}} \bigg ) , \end{aligned}$$

where $M^{(0)}_{{{\textsc {fdr}}}}(s)$ is the fully inclusive Born matrix element squared of $e^+ e^-\rightarrow \gamma ^*\rightarrow q(k_1)\bar{q}(k_2)$,

5.31 $$\begin{aligned} M^{(0)}_{{{\textsc {fdr}}}}(s) = \frac{2}{\pi } \int \limits _{\Phi _2} M^{(0)}_{{{\textsc {fdr}}}}(k_1,k_2) . \end{aligned}$$

In accordance with Eq. (5.18), we now replace all the invariants by their massive counterparts, $s_{ij} \rightarrow \bar{s}_{ij}$, and integrate over a $\mu$-massive three-body phase space,

5.32 $$\begin{aligned}&\int \limits _{\bar{\Phi }_3} M_{{{\textsc {fdr}}}}^{(0)}(\bar{s}_{12},\bar{s}_{13},\bar{s}_{23}) = \frac{4 \pi ^3 \alpha _s}{s^2} {C_F}M^{(0)}_{{{\textsc {fdr}}}}(s)\nonumber \\&\quad \times \int \limits _{\bar{R}_3} d\bar{s}_{13} d\bar{s}_{23} \bigg ( -\frac{s}{ \bar{s}_{13}} -\frac{s}{ \bar{s}_{23}} +\frac{ \bar{s}_{13}}{2 \bar{s}_{23}} +\frac{ \bar{s}_{23}}{2 \bar{s}_{13}} +\frac{s^2}{\bar{s}_{13} \bar{s}_{23}} \bigg ) . \end{aligned}$$

The quantity $\bar{R}_3$ represents the physical region of the Dalitz plot for the $\mu$-massive three-particle phase-space parametrized in terms of $\bar{s}_{13}$ and $\bar{s}_{23}$. The limit $\mu \rightarrow 0$ is understood from now on. The needed integrals can be expressed in terms of the scaled invariants

5.33 $$\begin{aligned} \bar{x}&= \frac{\bar{s}_{13}}{s}-\mu _0 ,\quad \bar{y}= \frac{\bar{s}_{23}}{s}-\mu _0 ,\quad \nonumber \\ \bar{z}&= \frac{\bar{s}_{12}}{s}-\mu _0 ,\quad \mathrm{with}\quad \mu _0=\frac{\mu ^2}{s} , \end{aligned}$$

and they are listed in Ref. [92]. We report them here for completeness^25^

5.34a $$\begin{aligned}&\int \limits _{\bar{R}_3} d\bar{s}_{13} d\bar{s}_{23} \frac{1}{\bar{s}_{13}} =\int _{\bar{R}_3} d\bar{s}_{13} d\bar{s}_{23} \frac{1}{\bar{s}_{23}}\nonumber \\&\quad =s\int \limits _{\bar{R}_3} d \bar{x} d \bar{y} \frac{1}{\bar{y}+\mu _0} =s \bigg [ -\ln (\mu _0) -3 +\mathcal {O}(\mu _0) \bigg ] , \phantom {\bigg |} \end{aligned}$$

5.34b $$\begin{aligned}&\int \limits _{\bar{R}_3} d\bar{s}_{13} d\bar{s}_{23} \frac{\bar{s}_{13}}{\bar{s}_{23}} =\int \limits _{\bar{R}_3} d\bar{s}_{13} d\bar{s}_{23} \frac{\bar{s}_{23}}{\bar{s}_{13}}\nonumber \\&\quad =s^2 \int \limits _{\bar{R}_3} d \bar{x} d \bar{y} \frac{\bar{y}}{\bar{x}+\mu _0} =s^2\bigg [ -\frac{\ln (\mu _0)}{2} -\frac{7}{4} +\mathcal {O}(\mu _0)\bigg ] , \end{aligned}$$

5.34c $$\begin{aligned}&\int \limits _{\bar{R}_3} d\bar{s}_{13} d\bar{s}_{23} \frac{1}{\bar{s}_{13} \bar{s}_{23}} =\int \limits _{\bar{R}_3} d \bar{x} d \bar{y} \frac{1}{(\bar{x}+\mu _0)(\bar{y}+\mu _0)}\nonumber \\&\quad =\frac{\ln ^2(\mu _0)}{2}-\frac{\pi ^2}{2}+\mathcal {O}(\mu _0) . \phantom {\bigg |} \end{aligned}$$

The final result of the bremsstrahlung contribution reads^26^

5.35 $$\begin{aligned} \sigma _{{{\textsc {fdr}}}}^{(r)}&= \sigma ^{(0)}\left( \frac{\alpha _{s}}{\pi }\right) {C_F}\bigg [ \frac{\ln ^{2}(\mu _{0})}{2} +\frac{3}{2}\ln (\mu _0)\nonumber \\&\quad +\frac{17}{4} -\frac{\pi ^{2}}{2} + \mathcal {O}(\mu _0) \bigg ] . \phantom {\Bigg |} \end{aligned}$$

Adding the virtual contribution given in Eq. (5.29) produces the total NLO correction,

5.36 $$\begin{aligned} \sigma ^{(1)}&= \sigma ^{(0)} + \sigma ^{(v)}_{{{\textsc {fdr}}}} + \sigma ^{(r)}_{{{\textsc {fdr}}}}\bigg |_{\mu _0\rightarrow 0} = \frac{{Q_{q}^{2}}N_c}{3 s}\bigg (\frac{e^4}{4\pi }\bigg )\nonumber \\&\quad \times \bigg [ 1+\bigg (\frac{\alpha _s}{4\pi }\bigg ) 3 {C_F}\bigg ] . \phantom {\Bigg |} \end{aligned}$$

Finally, we remark that it is possible to set up the entire calculation in a fully local fashion. To achieve this, one has to rewrite the double and single logarithms in Eq. (5.29) as local counterterms to be added to the real integrand. For instance, Eq. (5.34c) gives

5.37 $$\begin{aligned} \ln ^2(\mu _0)-\pi ^2 = 2 \int \limits _{\bar{R}_3} d\bar{s}_{13} d\bar{s}_{23} \frac{1}{\bar{s}_{13} \bar{s}_{23}} . \end{aligned}$$

The full counterterm needed for the case at hand can be inferred uniquely from the factorization properties of the matrix element squared,

5.38 $$\begin{aligned}&M^{{{\textsc {ct}}}}_{{{\textsc {fdr}}}}(p_1,p_2,p_3)\nonumber \\&\quad = \frac{16 \pi \alpha _s}{s} {C_F}M^{(0)}_{{{\textsc {fdr}}}}(\hat{p}_1,\hat{p}_2)\nonumber \\&\qquad \times \bigg ( -\frac{s}{\bar{s}_{13}} -\frac{s}{\bar{s}_{23}} +\frac{\bar{s}_{13}}{2\bar{s}_{23}} +\frac{\bar{s}_{23}}{2\bar{s}_{13}} +\frac{s^2}{\bar{s}_{13} \bar{s}_{23}} -\frac{17}{2} \bigg ) . \end{aligned}$$

This equation is in agreement with Eq. (5.30) when integrating over $\hat{p}_1$ and $\hat{p}_2$. The constant $\frac{17}{2}$ is chosen in such a way that only the logarithms and the $\pi ^2$ term in Eq. (5.35) are reproduced upon integration over $\bar{R}_3$. The quantity $M^{(0)}_{{{\textsc {fdr}}}}(\hat{p}_1,\hat{p}_2)$ is computed with mapped quark and antiquark momenta defined as

5.39 $$\begin{aligned} \hat{p}_1^{\alpha }&= \kappa \Lambda ^{\alpha }_{\phantom {\alpha }\beta } p_1^\beta \bigg (1+\frac{s_{23}}{s_{12}}\bigg ),\quad \hat{p}_2^{\alpha } = \kappa \Lambda ^{\alpha }_{\phantom {\alpha }\beta } p_2^\beta \bigg (1+\frac{s_{13}}{s_{12}}\bigg ),\nonumber \\ \kappa&= \sqrt{\frac{s s_{12}}{(s_{12}+s_{13})(s_{12}+s_{23})}} , \end{aligned}$$

where $\Lambda ^{\alpha }_{\phantom {\alpha }\beta }$ is the boost that brings the sum of the momenta back to the original center of mass frame, $\hat{p}_1+\hat{p}_2= (\sqrt{s},0,0,0)$. After subtracting $M^{{{\textsc {ct}}}}_{{{\textsc {fdr}}}}(p_1,p_2,p_3)$ from the exact matrix element squared, $\mu$ can be set to zero before integration. In this case, an analytic knowledge of $M_{{{\textsc {fdr}}}}^{(0)}(s_{12},s_{13},s_{23})$ is not necessary. A simple flat Monte Carlo with $10^5$ phase-space points reproduces the result in Eq. (5.36) at the 1 per mil level in a quarter of second.

### Established properties and future developments of FDR

#### Correspondence between integrals in FDR and DS

At one loop, a one-to-one correspondence exists between integrals regularized in fdr and ds. More precisely, according to the definition of fdr, any result of a loop integration is UV finite, whereas IR divergences are expressed in powers of (logarithms of) $\mu _0=\mu ^2/s$. In ds, on the other hand, results of an integration in ${d}$ dimensions can be expanded in powers of $\epsilon$; UV and IR divergences are then parametrized as poles $1/\epsilon ^n$.

To provide an example for the relation between IR divergences of integrals in fdr and ds, we consider the integral in Eq. (5.14). Using ${d}$-dimensional integration, its result reads

5.40 $$\begin{aligned} I_{{{\textsc {ds}}}} =c_{\Gamma }(\epsilon ) \frac{i\pi ^2}{s} \bigg [ \frac{1}{\epsilon ^2} +\frac{i\pi }{\epsilon } -\frac{\pi ^2}{2}+\mathcal {O}(\epsilon ) \bigg ] . \end{aligned}$$

The factor $c_{\Gamma }(\epsilon )$ is directly related to integration in ${d}$ dimensions. It is given in Eq. (2.20b). Comparing the result in Eq. (5.40) with Eq. (5.14b), the relation between the (regularized) IR divergences is given by

5.41 $$\begin{aligned} \frac{1}{\epsilon ^2}\leftrightarrow \frac{1}{2} \text {ln}^2(\mu _0) , \quad \frac{1}{\epsilon }\leftrightarrow \text {ln}(\mu _0) . \end{aligned}$$

Extending this to the ‘finite’ terms, the following generalized relation for a (potentially UV and IR divergent) integral over a generic integrand *F* holds:

5.42 $$\begin{aligned}&\bigg [\frac{1}{(2\pi )^{4}}\int [d^4q] F(\bar{q}^2,q)\bigg ]_{\mu ^0}\nonumber \\&\quad =\bigg [c_{\Gamma }(\epsilon )^{-1} \mu ^{4-{d}}\int \frac{d^d q}{(2\pi )^{{d}}} F(q^2,q) \bigg ]_{\epsilon ^0} . \end{aligned}$$

Analogously, for the real contribution one finds

5.43 $$\begin{aligned}&\Bigg [ \int \limits _{\bar{R}_3} d\bar{x} d\bar{y} d\bar{z}\ F(\bar{x},\bar{y},\bar{z})\ \delta (1-\bar{x}-\bar{y}-\bar{z})\Bigg ]_{\mu ^0}\nonumber \\&\quad =\Bigg [ \bigg (\frac{\mu ^2}{s}\bigg )^\epsilon \int \limits _{R_3} dx dy dz\ F(x,y,z)\ \frac{\delta (1-x-y-z)}{(x y z)^{\epsilon }} \Bigg ]_{\epsilon ^0} , \end{aligned}$$

where $R_3, x, y$, and *z* are the massless counterparts of $\bar{R}_3, \bar{x}, \bar{y}$, and $\bar{z}$, respectively; see also Eq. (5.33).

Finally, there exists a connection between the fdr ‘extra integrals’ and fdf integrals containing powers of the $(-2\epsilon )$-dimensional part of the loop momentum, $q_{[-2\epsilon ]}\equiv \tilde{q}$,

5.44 $$\begin{aligned} \int [d^4q] F(\bar{q}^2,q,-\mu ^2) =\mu ^{4-{d}}\int d^dq F(q^2,q, \tilde{q}^2) . \end{aligned}$$

For more comments on the interplay $-\mu ^2\leftrightarrow \tilde{q}^2$; see also the discussion around Eq. (3.6) and Ref. [96].

#### Gauge invariance, unitarity, and extra integrals

Global prescriptions, such as the one described at one loop in Eq. (5.20), can be defined at any loop order. Their role is maintaining the needed gauge cancellations. However, this is not enough to guarantee that results are compatible with unitarity. In fact, in a unitary QFT, all perturbative orders are linked by unitarity relations, and any renormalization procedure compatible with unitarity has to fulfill the following two requirements:

- The UV divergences generated at any perturbative level should have no influence on the next perturbative orders.
- The subintegration consistency in Eq. (5.4) should hold true.

Schemes based on ds automatically respect subintegration consistency when all objects (including $\gamma$ matrices) are treated in *d* dimensions, while requirement (a) is fulfilled only if $1/\epsilon$ poles are subtracted order by order by introducing counterterms in $\mathcal{L}$. This forbids one to define ds loop integrals beyond one loop by simply dropping $1/\epsilon$ poles. See the discussion is Section 2.5 of Ref. [99] for more details.

On the other hand, fdr automatically respects requirement (a) since the UV subtraction is embedded in the definition of the fdr integral, so that there is no room for any UV divergence to have any influence at higher perturbative levels. For instance, products of two one-loop fdr integrals give the same result at any perturbative order, which is not the case in ds.

On the contrary, subintegration consistency is not automatically obeyed in fdr. The reason for this can be traced back to the fact that the global prescription needed at the level of divergent subdiagrams (sub-prescription) clashes with the global prescription required at the level of the full diagram (full-prescription), so that one has to correct for this mismatch. However, this can be done directly at a diagrammatic level. This is possible thanks to the fdr extra integrals. They can be used to parametrize, in an algebraic way, the difference between the result one gets when cancellations do or do not take place between numerators and denominators, as illustrated, for example, in Eq. (5.24). In practice, one looks at all possible UV divergent subdiagrams, adds the piece needed to restore the sub-prescription and subtracts the *wrong* behaviour induced in the subdiagram by the full-prescription. The net result of this process is the addition of fdr *extra–extra integrals* to the amplitude that enforce requirement (b) without the need of an order-by-order renormalization [101]. For example, a two-loop extra–extra integral can be defined as the insertion of a one-loop extra integral into a two-loop fdr integral. Thus, an fdr calculation directly produces renormalized quantities, which is a unique property of the fdr formalism.

Work is in progress to find the connection between fdr extra–extra integrals and evanescent fdh couplings. Preliminary results indicate that the introduction of fdr extra–extra integrals is equivalent to a restoration of the correct behaviour under renormalization in an fdh calculation in which one sets equal gauge and evanescent couplings from the beginning.

## FDU: four-dimensional unsubtraction

The four-dimensional unsubtraction (fdu) [103–107] approach constitutes an alternative to the traditional subtraction method. It is based on the loop–tree duality (LTD) theorem [108–111], which establishes a connection among loop and dual integrals, the latter being similar to standard phase-space integrals. In this way, the method provides a natural way to implement an integrand-level combination of real and virtual contributions, thus leading to a fully local cancellation of IR singularities. Moreover, the addition of local UV counterterms allows one to reproduce the proper results in standard renormalization schemes.

In the following, we describe briefly the general facts about the method, using the computation of the NLO QCD corrections to $\gamma ^{*}\rightarrow q \bar{q} (g)$ as a practical guideline.

### Introduction to LTD

The LTD theorem is based on Cauchy’s residue theorem. Let us consider a generic one-loop scalar integral for an *N*-particle process, where the external momenta are labeled as $p_i$ with $i\in \{1,2,\ldots N\}$, whilst the loop momentum is denoted by $\ell$. With these conventions, the internal virtual momenta become $q_{i} = \ell + k_i$ where $k_{i} = p_{1} + \cdots + p_{i}$ and $k_N=0$ because of momentum conservation. If the mass of the internal particles is $m_i$, a scalar integral can be expressed as

6.1 $$\begin{aligned} L^{(1)}(p_1, \dots , p_N) = \int _{\ell } \prod _{i=1}^{N} G_F(q_i) , \end{aligned}$$

with the Feynman propagators $G_F(q_i) = (q_i^2-m_i^2+i 0)^{-1}$. As usual, $q_i$ represents a four momentum which can be decomposed as $q_{i,\mu } = (q_{i,0},\mathbf {q}_i)$, independently of the specific space-time dimension.^27^ The energy component is $q_{i,0}$, whilst $\mathbf {q}_{i}$ denotes the spatial components.

At one-loop level, the dual representation of the loop integral is obtained by cutting one by one all the available internal lines and applying the residue theorem accordingly. The cut condition is implemented by restricting the integration measure through the introduction of

6.2 $$\begin{aligned} \tilde{\delta }\left( q_i\right) \equiv 2 \pi i \theta (q_{i,0}) \delta (q_i^2-m_i^2) , \end{aligned}$$

which transforms the loop integration domain into the positive energy section (i.e. $q_{i,0}>0$) of the corresponding on-shell hyperboloid (i.e. $q_i^2=m_i^2$). When the scattering amplitude under consideration is composed of single powers of the propagators, the computation of the residue simplifies to removing the cut propagator and replacing the uncut ones with their *duals*, i.e.

6.3 $$\begin{aligned} G_D(q_i;q_j) = \frac{1}{q_j^2 -m_j^2 - i 0 \eta \cdot k_{ji}} , \end{aligned}$$

where $i,j \in \{1,2,\ldots N\}$, $k_{ji}= q_j - q_i$ and $\eta$ is an arbitrary future-like or light-like vector, $\eta ^2 \ge 0$, with positive definite energy $\eta _0 > 0$. It is worth noticing that the dual prescription takes care of the multiple-cut correlations introduced in the traditional Feynman-tree theorem (FTT) [112, 113], thus allowing one to prove their formal equivalence.

In this way, the *dual integrand* looks like a tree-level amplitude whose building blocks are the same as in the standard theory with a modified *i*0 prescription. Thus, the one-loop scalar integral in Eq. (6.1) reads

6.4 $$\begin{aligned} L^{(1)}(p_1, \dots , p_N) = - \sum _{i=1}^N \int _{\ell } \tilde{\delta }\left( q_i\right) \prod _{j\ne i} G_D(q_i;q_j) . \end{aligned}$$

The existence of a dual representation for loop integrals straightforwardly leads to a dual representation for loop scattering amplitudes. As explained in Ref. [108], any loop contribution to scattering amplitudes in any relativistic, local, and unitary quantum field theory can be computed through the decomposition into *dual contributions*. Of course, this idea generalizes to multi-loop amplitudes, where dual contributions involve iterated single-cuts [108, 110].

For amplitudes containing higher powers of the propagators, the previous result can be extended, as studied in Ref. [111]. It is worth appreciating that higher powers of the propagators explicitly manifest when dealing with self-energy corrections at one loop, self-energy insertions at higher orders, and when computing the local version of the UV counterterms [104, 105].

### Momentum mapping and IR singularities

The application of the LTD theorem to a virtual amplitude leads to a set of dual contributions. From them, we can extract useful information as regards the location of the singularities in the corresponding integration domain, as well as the components (or cuts) that originate them. As explained in Refs. [114–116], the intersection of forward and backward hyperboloids defined by the on-shell conditions allows one to identify the IR (and threshold) singularities. Moreover, this study is crucial to prove the compactness of the region developing IR divergences [103–105], which constitutes a very important result by itself. This is because the real-radiation contributions are computed on a physical phase space, which is also compact.^28^ In consequence, since the Kinoshita–Lee–Nauenberg (KLN) theorem states that there is a cross-cancellation of IR singularities between real and virtual terms, the compactness of the IR region inside the dual integration domain allows one to implement a local real-virtual cancellation of singularities by applying a suitable momentum mapping. In this way, the singularities in the real phase space (PS) are mapped to the dual integration domain where the corresponding virtual singularities are generated; then an integrand-level cancellation takes place and there is no need of introducing any external regulator to render the combination integrable.

**Fig. 12:**
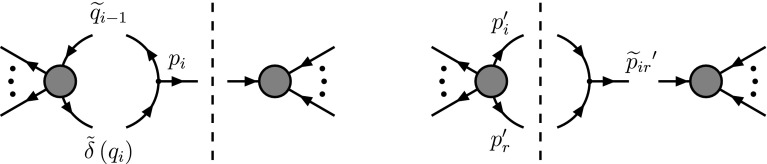
Diagrammatic contributions in the collinear limit, for both the dual one-loop (*left*) and the real-emission tree-level squared amplitudes (*right*). The *lines* that are crossed by a *dashed line* correspond to on-shell states. When particles are collinear, the parent becomes on-shell and the diagrams factorize

In order to connect the Born kinematics (*m*-particle PS) with the real- emission one ($(m+1$)-particle PS), we rely on techniques similar to those applied for the dipole method [53, 117]. To be more concrete, let us start thinking about the virtual contribution. After obtaining the dual amplitudes, we have a set of *m* external momenta and a free on-shell loop momentum. In this way, the dual amplitudes introduce an *extra* on-shell momentum. Since there are $(m+1)$ on-shell momenta available, the kinematics of the dual components exactly matches the kinematics of the real contribution.

Then it is necessary to isolate the real-emission IR singularities by properly splitting the complete real PS. If $p_i'^{\mu }$ are the momenta of the real-emission partons, we start by defining the partition

6.5 $$\begin{aligned} \mathcal{R}_i = \{{y'}_{i r} < \mathrm{min} {y'}_{jk} \} , \quad \sum _{i=1}^{m} \mathcal{R}_i =1 , \end{aligned}$$

where ${y'}_{ij}=2 p_i' \cdot p_j'/Q^2$, *r* is the radiated parton from parton *i*, and *Q* is the typical hard scale of the scattering process. It is important to notice that, inside $\mathcal{R}_i$, the only allowed collinear/soft configurations are $i\parallel r$ or $p_r'^{\mu }\rightarrow 0$. Thus, collinear singularities manifest in non-overlapping regions of the real-emission PS which allows one to introduce an optimized transformation to describe the collinear configuration.

On the other hand, there are *m* dual contributions, each one associated with a single cut of an internal line. So, we can establish an identification among partitions and dual amplitudes, based on the picture shown in Fig. 12. Concretely, the cut-line in the dual amplitude must be interpreted as the extra-radiated particle in the real contribution; i.e. $q_i \leftrightarrow p_r'$. Then we settle in one of the partitions, for instance $\mathcal{R}_i$. Because the only collinear singularity allowed is originated by $i \parallel r$, we distinguish particle *i* and call it the *emitter*. After that, we single out all the squared amplitude-level diagrams in the real contribution that become singular when $i \parallel r$ and cut the line *i*. These have to be topologically compared with the dual-Born interference diagrams whose internal momenta $q_i$ are on-shell (i.e. the line *i* is cut), as suggested in Fig. 12. In conclusion, the dual contribution *i* is to be combined with the real contribution coming from region $\mathcal{R}_i$.

The required momentum mapping is motivated by general factorization properties in QCD [114, 118] and the topological identification in Fig. 12. Explicitly, let us take the $(m+1)$-particle real-emission kinematics, with *i* as the emitter and *r* as the radiated particle, and we introduce a reference momentum, associated to the spectator *j*. For the massless case, the generic multi-leg momentum mapping with $q_i$ on-shell is given by

6.6 $$\begin{aligned}&p_i'^\mu = p_i^\mu - q_i^\mu + \alpha _i p_j^\mu , \quad p_j'^\mu = (1-\alpha _i) p_j^\mu ,\nonumber \\&p_k'^\mu = p_k^\mu \ \ k \ne i,j , \nonumber \\&p_r'^\mu = q_i^\mu , \quad \alpha _i = \frac{(q_i-p_i)^2}{2 p_j\cdot (q_i-p_i)} , \end{aligned}$$

with the primed momenta associated to the particles involved in the real-emission process. In this case, note that $p_i'^2=p_j'^2=p_r'^2=0$ because we restrict ourselves to massless particles. On the other hand, the initial-state momenta ($p_a$ and $p_b$) are not altered by the transformation, neither is $p'_k$ with $k\ne i,j$. Besides that, since

6.7 $$\begin{aligned} p_i+ p_j +\sum _{k\ne i,j} p_k = p_i'+p_r'+p_j'+\sum _{k\ne i,j,r} p_k'\ , \end{aligned}$$

the transformation shows momentum conservation. It is worth appreciating that this momentum mapping can be extended to the massive case, even if the involved particles have different masses, as we explained in Ref. [105].

### Integrand-level renormalization and self-energies

Besides dealing with IR singularities, any attempt to provide a complete framework for higher-order computations must be able to treat UV divergences. In this case, a suitable local version of the UV counterterms is required. This topic is deeply discussed in Ref. [104] for the massless case, whilst the massive one is studied in Ref. [105]. In the last case, the self-energy and vertex corrections become non-trivial and some technical subtleties arise: there are noticeable changes in the IR singular structure compared to the massless case. On one hand, the mass acts as an IR regulator, preventing collinear singularities to emerge. But, on the other hand, soft singularities arising from gluon emissions become non-vanishing because they are proportional to the mass of the emitting leg. Since we are looking for a complete local cancellation of singularities and a smooth massless transition, it is necessary that the expressions for the massive case reduce to those already available for massless processes, even at the *integrand level*.

Let us start with the well-known expression for the wave-function renormalization. Working in Feynman gauge with on-shell renormalization conditions, its integrated form is given by^29^

6.8 $$\begin{aligned} \Delta Z_2 = \bigg (\frac{\alpha _{s}}{4\pi }\bigg ) {C_F}\left[ -\frac{1}{\epsilon _{{{\textsc {uv}}}}}-\frac{2}{\epsilon _{{{\textsc {ir}}}}} + 3 \ln \left( \frac{M^2}{\mu ^2}\right) -4\right] ,\nonumber \\ \end{aligned}$$

where we kept track of the IR and UV origin of the $\epsilon$ poles within ds. The unintegrated expression [105] is given by

6.9 $$\begin{aligned}&\Delta Z_2(p_1)\nonumber \\&\quad = -g_{s}^2 {C_F}\int _{\ell } G_F(q_1) G_F(q_3) \nonumber \\&\qquad \times \left[ (d-2)\frac{q_1 \cdot p_2}{p_1 \cdot p_2} \right. +4 M^2 \left. \left( 1- \frac{q_1 \cdot p_2}{p_1 \cdot p_2}\right) G_F(q_3)\right] ,\nonumber \\ \end{aligned}$$

which includes higher-order powers of the propagators, and where we define $q_1=\ell +p_1$, $q_2=\ell +p_1+p_2$, and $q_3=\ell$. It is worth appreciating that there are many equivalent integrand-level expressions to describe $\Delta Z_2(p_1)$, but the one presented in Eq. (6.9) develops the proper IR behaviour to cancel singularities coming from real-emission processes. Besides this, notice that the corresponding formula for the massless case [104] is simply recovered by considering $M\rightarrow 0$ at the integrand level. The term proportional to $M^2$ is responsible for soft divergences that appear when $q_1$ is set on-shell, and it vanishes as $M \rightarrow 0$ since soft singularities are absent in the massless self-energy computation. On the contrary, the collinear singularities that appear in $\Delta Z_2(M=0)$ manifest themselves as quasi-collinear divergences, i.e. terms that behave like $\ln (M^2/\mu ^2)$, as shown in Eq. (6.8). Once we combine the self-energy contributions with the virtual matrix elements, there are still UV singularities present. These have to be removed by performing an expansion around the UV propagator $G_F(q_{{\textsc {uv}}}) =(q_{{\textsc {uv}}}^2-\mu _{{\textsc {uv}}}^2+i 0)^{-1}$,

6.10 $$\begin{aligned} G_F(q_i)&= G_F(q_{{{\textsc {uv}}}}) \nonumber \\&\quad \left( 1- \frac{2q_{{\textsc {uv}}} \cdot k_{i,{{\textsc {uv}}}}+k_{i,{{\textsc {uv}}}}^2+\mu _{{\textsc {uv}}}^2-m_i^2}{q_{{\textsc {uv}}}^2-\mu _{{\textsc {uv}}}^2+i 0}+ \ldots \right) , \end{aligned}$$

with the renormalization scale $\mu _{{\textsc {uv}}}$ and $k_{i,{{\textsc {uv}}}}=q_i-q_{{\textsc {uv}}}$. A similar expansion is carried out in the numerator, which leads to the UV counterterm for the wave-function renormalization,

6.11 $$\begin{aligned}&\Delta Z_2^{{{\textsc {uv}}}}(p_1) \nonumber \\&\quad = (2-d) g_{s}^2 {C_F}\int _{\ell } \big [G_F(q_{{\textsc {uv}}})\big ]^2 \nonumber \\&\quad \quad \times \left( 1+\frac{q_{{\textsc {uv}}} \cdot p_2}{p_1 \cdot p_2}\right) [1-G_F(q_{{\textsc {uv}}})(2 q_{{\textsc {uv}}} \cdot p_1 + \mu ^2_{{\textsc {uv}}}) ] \nonumber \\&\quad = - (4\pi )^{\epsilon } \Gamma (1+\epsilon ) \frac{\alpha _{s}}{4\pi }{C_F}\left( \frac{\mu _{{\textsc {uv}}}^2}{\mu ^2}\right) ^{-\epsilon } \frac{1-\epsilon ^2}{\epsilon }. \end{aligned}$$

The integrated form exactly reproduces the UV pole present in Eq. (6.8). The subleading terms proportional to $\mu _{{\textsc {uv}}}^2$ are chosen to subtract only the pole part from Eq. (6.8) and, in this way, settle in the ${\overline{{{\textsc {ms}}}}}$ scheme. Finally, we define the UV-free wave-function renormalization

6.12 $$\begin{aligned} \Delta Z_2^{{{\textsc {ir}}}} = \Delta Z_2 - \Delta Z_2^{{{\textsc {uv}}}} , \end{aligned}$$

that only contains IR singularities. To conclude this discussion, it is important to emphasize that this construction is completely general and that the subleading terms can be adjusted to reproduce the desired scheme-dependent contributions.

Besides the wave-function renormalization, it is also necessary to remove the UV singularities associated to the vertex corrections. The corresponding renormalization counterterm in its unintegrated form is given by6.13where ${\Gamma }^{(0)}_{A}$ represents the tree-level vertex. Again, the term proportional to $\mu _{{{\textsc {uv}}}}^2$ in the numerator is subleading in the UV limit and its coefficient, $d_{A,{{\textsc {uv}}}}$, must be adjusted in order to implement the desired renormalization scheme [105].

### Application example: $e^{+} e^{-}\rightarrow \gamma ^{*}\rightarrow q\bar{q}$ at NLO

In order to compute the NLO QCD corrections to $e^{+} e^{-}\rightarrow \gamma ^{*}\rightarrow q\bar{q}$, we start from the complete set of $\mathcal{O}(\alpha _{s}^2)$ real and virtual diagrams, including the self-energy ones. The total *unrenormalized* virtual cross section is

6.14 $$\begin{aligned} \sigma ^{(v)}_{{{\textsc {fdu}}}}&= \frac{1}{2 s_{12}} \int d\Phi _{1\rightarrow 2} \{ 2 {\text {Re}} \langle \mathcal{A}^{(0)}_{{{\textsc {fdu}}}}| \mathcal{A}^{(1)}_{{{\textsc {fdu}}}} \rangle \nonumber \\&\quad + \big [\Delta Z_2(p_1)+\Delta Z_2(p_2)\big ] M^{(0)}_{{{\textsc {fdu}}}} \} , \end{aligned}$$

where we distinguish contributions originated in the triangle diagram from those related to self-energies. After that, we must introduce the local UV counterterms which implements the desired renormalization scheme and replace the self-energy contributions by the wave-function renormalization constants, $\Delta Z_2^{{\textsc {ir}}}$. In this case, we apply LTD to Eq. (6.14) and obtain a set of three dual contributions, $\widetilde{\sigma }^{(v)}_{i,{{\textsc {fdu}}}}$.

Once the dual contributions are computed, we turn attention to the real-emission terms. As explained in Sect. 6.2, we need to isolate the different collinear singularities by introducing a partition of the real phase space. This leads to

6.15 $$\begin{aligned}&\widetilde{\sigma }^{(r)}_{i,{{\textsc {fdu}}}} = \frac{1}{2 s_{12}} \int d\Phi _{1\rightarrow 3} M_{{{\textsc {fdu}}}}^{(0)}({q \bar{q} g}) \theta (y_{jr}' -y_{ir}' ) \nonumber \\&\quad i,j \in \{ 1,2\} , \quad i \ne j , \end{aligned}$$

which fulfills $\widetilde{\sigma }^{(r)}_{1,{{\textsc {fdu}}}}+ \widetilde{\sigma }^{(r)}_{2,{{\textsc {fdu}}}} = \sigma ^{(r)}_{{{\textsc {fdu}}}}$. After that, we apply the real-virtual mapping in each partition. This converts the real terms into fully local IR counterterms for the dual contributions; this guarantees a complete cancellation of IR singularities at the integrand level, thus rendering the full expression integrable in four dimensions. This is a really important fact, because it allows one to put aside ds safely by directly considering the limit $\epsilon \rightarrow 0$ at the integrand level [103]. Finally, the master formula for computing the *finite* cross-section correction is

6.16 $$\begin{aligned} \sigma ^{(1)} = \mathcal{T}\left( \sum _{i=1}^3 \widetilde{\sigma }^{(v)}_{i,{{\textsc {fdu}}}} + \sum _{j=1}^2 \widetilde{\sigma }^{(r)}_{j,{{\textsc {fdu}}}}\right) - \widetilde{\sigma }^{{{\textsc {uv}}}} , \end{aligned}$$

where $\widetilde{\sigma }^{{{\textsc {uv}}}}$ is the dual representation of the local UV counterterms and $\mathcal{T}$ is an operator that implements the unification of dual coordinates at the integrand level (with the corresponding Jacobians). If we add all the contributions at the integrand level and deal with a single master integration, the expression in Eq. (6.16) is directly implementable in four space-time dimensions and leads to the correct result after numerical computation. It is worth mentioning that, in order to improve the numerical stability, it helps to compactify the integration domain, applying a transformation as suggested in Ref. [105].

### Further considerations and comparison with other schemes

As we depicted in the previous paragraphs, the fdu approach is based on a fully local cancellation of IR and UV singularities in strictly four dimensions. In this way, we avoid many of the practical/conceptual problems related to the extension of physical properties to *d* space-time dimensions. In particular, the $\gamma ^5$ issue is naturally absent here. Moreover, the idea of using the mapped real contributions as local IR counterterms for the dual part simplifies the treatment of IR divergences, as well as it provides a better understanding of their origin.

On the other hand, the application of the traditional renormalization procedure within this framework implies to recompute the renormalization constants in an unintegrated form (i.e. for the integrand-level implementation). In any case, by fixing subleading terms in the UV expansion it is possible to specify the finite part of the counterterms, thus reproducing the results in any scheme (for instance, in $\overline{\mathrm{MS}}$). Moreover, this algorithm is completely process-independent and, in consequence, fully compatible with higher-order computations. In this sense, the treatment of UV divergences is similar to the procedure proposed within fdr. The main difference is that we transform the local counterterms to the dual space, in order to combine it with virtual amplitudes.

Besides this, it is worth mentioning that LTD can handle loop amplitudes, as any other method described in this report, but fdu is designed to work directly with physical observables. For instance in Ref. [119], we applied our framework to deal with the Higgs boson decay to massless gauge bosons, which although known to be finite still requires a proper regularization due to the fact that the amplitudes are UV singular locally.

Finally, we would like to emphasize that fdu is compatible with the desired requirements mentioned in the introduction. In fact, since it is a four-dimensional approach which relies on proper physically motivated changes of variables, fdu does not alter the four-dimensional properties of the underlying theory (i.e. unitarity, causality, and associated symmetries). Moreover, it fulfills the crucial requirement of mathematical consistency because singularities are completely removed by a local mapping. In this way, all the singularities are canceled before they manifest themselves in the integration.

## Summary and outlook 

The vast majority of higher-order calculations are done using cdr. While there is no doubt that this made possible impressive progress in perturbative calculations, there is a certain danger that this success stifles the progress of other methods. Whether such alternative methods will ever result in a viable way to perform actual computations can only be established by actually using them. In order to facilitate this, this article provides an overview of recent (and not so recent) developments of regularization schemes other than cdr. Some are very close to cdr, for others the differences are much larger. Using simple examples, we have illustrated the differences and similarities of these methods and their relation to cdr. Let us summarize the key points by means of the following list.

- *FDH and DRED* are perfectly consistent regularizations schemes, at least up to NNLO. However, they require the introduction of additional (evanescent) couplings with (in general) different counterterms. In non-supersymmetric theories, for dred this is already mandatory at NLO, for fdh this is unavoidable only at NNLO and beyond. Supersymmetry might protect the equivalence of the couplings even beyond these approximations. Statements in the literature that fdh is inconsistent always refer to ‘naive fdh’, i.e. fdh without distinguishing the couplings.
- *Conversions* between results in cdr, hv, fdh, and dred can be made for individual parts contributing to a cross section. For the virtual contributions this is known to NNLO and can be elegantly described solely through the scheme dependence of $\beta$ functions and anomalous dimensions. For real corrections and initial-state factorization terms the explicit scheme dependence is only known to NLO. These results have been used to explicitly demonstrate the scheme independence of a cross section at NLO.
- *FDF* is an adaption of the (naive) fdh scheme that can be used in strictly four dimensions. This enables the use of unitarity methods, writing loop integrands as products of tree-level amplitudes and performing numerical calculations with the components of spinors and momenta. At NLO, fdf gives results that are equivalent to fdh. How to extend this beyond NLO is currently under investigation. The scalars of fdf are not identical to the $\epsilon$-scalars of fdh.
- *GoSam* makes use of fdf and other four-dimensional techniques. The one-loop virtual amplitudes that are called ‘dred’ and ‘cdr’ in GoSam correspond to what we call ‘naive fdh’ and ’hv’, respectively, in this article. Virtual one-loop amplitudes in other schemes are obtained indirectly through conversion formulae.
- *SDF* is based on the same idea as fdf. However, having two-loop amplitudes in mind, the integer dimension is set to ${d_{e}}=6$. Hence, the spinor formalism has to be extended to 6 dimensions.
- *UV singularities in IREG and FDR* The basic idea of ireg and fdr is similar and based on the observation that UV singularities are independent of the kinematics. This is used to isolate the UV singular part of loop integrals. In ireg, the UV singular part is expressed in terms of (implicit) integrals $I_{\text {log}}$ and boundary terms (which have to be set to zero to respect gauge invariance), whereas in fdr they are set to zero. The resulting UV-finite integrals are evaluated in (strictly) four dimensions.
- *IR singularities in IREG and FDR* are also treated in strictly four dimensions. The matrix elements squared are computed for massless particles (in four dimensions) and the phase-space integration is also carried out in four dimensions. IR singularities are regularized by modifying the phase-space boundaries through a shift $q\rightarrow q+\mu$ and result in logarithms $\ln (\mu _0)=\ln (\mu ^2/s)$. In this sense the method is similar to the introduction of a photon or gluon mass. However, the procedures used by ireg and fdr are superior as they preserve gauge invariance.
- *Differences between IREG and FDR* In ireg, gauge invariance is achieved by performing first the Dirac algebra in the numerator and then cancel terms in the numerator and denominator before the shift $q\rightarrow q+\mu$. In fdr, the shift is done universally in the numerator and denominator. Then additional terms with $\mu ^2$ in the numerator (called ‘extra integrals’) are included. ireg produces expressions where the UV singularities are still present in the form of implicit integrals $I_{\text {log}}$. They have to be removed by a suitable renormalization procedure, as in ds. Applying fdr, on the other hand, results directly in UV renormalized quantities.
- *Relation between IREG/FDR and dimensional schemes:* In ireg and fdr, ‘singularities’ related to real contributions are encoded in powers of $\ln (\mu _0)$. At NLO, there is a direct mapping between these terms and the $1/\epsilon ^n$ singularities in the fdh scheme, namely $1/\epsilon ^2 \leftrightarrow 1/2 \ln ^2(\mu _0)$ and $1/\epsilon \leftrightarrow \ln (\mu _0)$. The extension to NNLO of such a correspondence between the four-dimensional schemes and the traditional dimensional schemes is under active investigation. This also includes on how to compensate for the absence of evanescent couplings in ireg and fdr.
- *FDU* is an even more radical method in that it does not split a cross section into (potentially IR divergent) virtual and real parts. Rather, the combination of the two parts (and thus the cancellation of IR singularities) is done at the integrand level. Local counterterms are used to perform ${\overline{{{\textsc {ms}}}}}$ renormalization. The extension to initial-state singularities is also possible; the application of a slightly modified momentum mapping allows one to cancel the soft singularities. The remaining initial-state collinear singularities can be canceled by adding unintegrated initial-state counterterms. This is currently under investigation.
- *Evanescent couplings* are a fact of life! Even though they can be avoided at NLO in some four-dimensional formulations (like fdh) or do not show up in some particular processes even at NNLO (like $g g\rightarrow g g$ in fdh), they are present in all (partly) four-dimensional regularizations of QED and QCD. In particular, they have an effect at NNLO in fdh (like e.g. for $g g\rightarrow q\bar{q}$). The connection of these effects to the ‘extra–extra integrals’ in fdr is under investigation.

The list above illustrates that there are promising alternatives available that at least at NLO are well understood. They can and have been used for NLO calculations and in some cases have proved to be more efficient.

Currently, a huge effort in perturbative calculations is made going beyond NLO towards automated computations at NNLO. Many of the schemes above have been revisited in the hope they provide a smoother road towards this goal. We are convinced that this deserves to be investigated more thoroughly. In any case, for an alternative scheme to be consistent, there must – at least in principle – exist a well-defined relation to cdr. At NNLO, these relations are fairly well established for other traditional dimensional schemes like hv, fdh, and dred. Regarding new formulations of dimensional schemes like fdf or non-dimensional schemes such as ireg and fdr, first steps towards establishing such relations have been made. fdu has the advantage that a separate regularization of the final-state IR singularities is not required, but only the UV singularities have to be treated in a well-defined way, such as ${\overline{{{\textsc {ms}}}}}$.

Comparing to the impressive list of NNLO calculations for physical cross sections that have been made using cdr, it is fair to say, that none of the other methods has had a similar impact so far. Since cdr is the best established scheme, it is tempting to keep using it. However, it is not clear at all, if cdr is really the most efficient scheme. Hence, the investigation of other regularization schemes is an important aspect of making further progress in perturbative computations. Are there more efficient dimensional schemes? Or is it ultimately advantageous to work completely in four dimensions?

That is the question.

## Footnotes

1 In this section and in Sect. 3, the (quasi)dimensionality $\mathrm{dim}$ of an object is indicated by a subscript $[\mathrm{dim}]$. In Sects. 4–6, where loop integrations are performed in strictly four dimensions, the subscript is suppressed unless stated otherwise.

2 In many original references, objects such as $\hat{\gamma }^\mu$, $\tilde{\gamma }^\mu$, $\breve{\gamma }^\mu$ are introduced with specific meanings which differ, depending on the paper, the scheme, and the context. Hence, we avoid such short-hand notations here and stick with a more explicit one to make the meaning of expressions more apparent.

3 In fdh and dred, $d_s$ is usually taken to be 4, and therefore $n_\epsilon =2\epsilon$.

4 Note that compared to Ref. [6] we replaced the terms ‘internal’ and ‘external’ by ‘singular’ and ‘regular’, respectively, to avoid possible confusion in later considerations.

5 Since $M^{(0)}_{{{\textsc {fdf}}}}\equiv M^{(0)}_{{{\textsc {fdh}}}}$, this result coincides with the one obtained in fdh for $n_\epsilon =2\epsilon$ and $g_e=g_s$ at least up to $\mathcal {O}(\epsilon ^0)$, compare with Eq. (2.19c).

6 This result can be compared to the one obtained in fdh; see Eq. (2.11). After subtraction of the UV divergence, the limit ${d}\rightarrow 4$ can be taken and both results coincide. However, due to the vanishing scalar contribution it is clear that the additional scalar field in fdf is different from the $\epsilon$-scalar of fdh.

7 The result of $Z_2$ in the on-shell scheme has already been obtained in Ref. [28] for the case of fdh. It coincides with Eq. (3.41a) for $n_\epsilon =2\epsilon$ and the case of equal couplings.

8 The scheme is actually called dimensional reduction in GoSam and in Ref. [23], but corresponds to what we call fdh in this article.

9 This point of view is shared by other methods as well, for instance by fdr which is described in Sect. 5. In the latter scheme, these intrinsic divergent pieces are called ‘vacua’.

10 This result can be compared with the corresponding one obtained in fdh; see Eq. (2.11). Setting the surface term $\upsilon _{0,2}$ to zero which is necessary to preserve gauge invariance; see also Sect. 4.3, the *finite* terms of the electron self-energy in ireg and ds are the same for ${d}=4$ and $\lambda ={\mu _{\textsc {ds}}}$. The relation for the UV divergence is given by $b^{-1}I_{\text {log}}(\lambda ^2)\leftrightarrow \frac{1}{\epsilon }$.

11 This is a crucial difference compared to fdr, where $\mu ^2$-terms remain in the numerators. In a second step, they are then removed by so-called ‘extra integrals’. Further discussions can be found in Sect. 4.3

12 The results of the ireg integrals can be compared with the corresponding ones in ds. Setting ${\mu _{\textsc {ds}}^{2}}=s$, the integrals in Eqs. (4.16a) and (4.16b), for example, are given by $I_{{{\textsc {ds}}}}\big |_{{p_q^2=p_{\bar{q}}^2=0}} =c_{\Gamma }(\epsilon ) \frac{i}{(4\pi )^2} \frac{1}{s} \big [ \frac{1}{\epsilon ^2} +\frac{i\pi }{\epsilon } -\frac{\pi ^2}{2}+\mathcal {O}(\epsilon ) \big ]$ and $I_{{{\textsc {ds}}}}^{\alpha }\big |_{{p_q^2=p_{\bar{q}}^2=0}} =c_{\Gamma }(\epsilon ) \frac{i}{(4\pi )^2} \frac{(p_q-p_{\bar{q}})^{\alpha }}{s} \big [ \frac{1}{\epsilon } +i\pi +2 +\mathcal {O}(\epsilon )\big ]$. Using $c_{\Gamma }(\epsilon =0) =1$, the one-to-one correspondence between double and single IR poles in ds and ireg then reads $\frac{1}{\epsilon ^2} \leftrightarrow \frac{1}{2}\ln ^2(\mu _0)$ and $\frac{1}{\epsilon } \leftrightarrow \ln (\mu _0)$; see also Sect. 5.4.

13 The virtual cross section in ireg can be compared with the ones obtained in ds; see Eq. (2.21). Using the aforementioned translation rules for IR divergences in ireg and ds, and $\Phi _2(\epsilon =0)=c_\Gamma (\epsilon =0)=1$, it follows that Eq. (4.18) coincides with the results obtained in fdh and dred, Eq. (2.21c). In Sect. 5.3, it will be shown that the result of the virtual cross section in ireg also coincides with the one obtained in fdr.

14 These integrals are the same in ireg and fdr; see e.g. Eqs. (34) and (35) of Ref. [92]. They can be compared with the corresponding ones obtained in ds; see Eqs. (2.26). Again, the transition rules for the IR divergences between ds and ireg/fdr read $\frac{1}{\epsilon ^2}\leftrightarrow \frac{1}{2}\ln ^{2}(\mu _{0})$ and $\frac{1}{\epsilon }\leftrightarrow \ln (\mu _{0})$.

15 This result can be compared with the ones obtained in ds; see Eq. (2.27). Using the rules for translating IR divergences between ireg and ds together with $\Phi _3(\epsilon =0)=1$, it follows that Eq. (4.32) coincides with the results in fdh and dred, Eq. (2.27c). In Sect. 5.3, it will be shown that Eq. (4.32) also coincides with the corresponding result in fdr.

16 The only technical difference from the evaluation of real corrections in fdr is that in fdr the matrix elements are computed in the strict massless limit, i.e. using $k_i^2=0$. Thus, at least at NLO the two schemes differ at most by terms $\mathcal {O}(\mu _0)$.

17 This result coincides with the well-known value of the QED $\beta$ function of the gauge coupling obtained in ds; see Eq. (2.8a).

18 This is different compared to ireg where no extra integrals are introduced. While extra integrals are not strictly needed in fdr, they are introduced for convenience to allow the decomposition of fdr tensor integrals into MIs and to avoid introducing counterterms in $\mathcal{L}$.

19 The alternative definition in Eq. (5.11) with, for example, r = ds gives the same result, $\int [d^4q] \frac{1}{(\bar{q}^2-M^2)^2} =\mu _{{{\textsc {ds}}}}^{4-{d}}\int d^dq \frac{1}{(q^2-M^2)^2} -\lim _{\mu \rightarrow 0}\mu _{{{\textsc {ds}}}}^{4-{d}} \int d^dq \frac{1}{\bar{q}^4}\bigg |_{\mu \rightarrow \mu _{{{\textsc {ds}}}}} =-i \pi ^2 \ln \frac{M^2}{\mu _{{{\textsc {ds}}}}^2}$ . In the first integral, $\mu$ can be directly set to zero since it is IR convergent.

20 This relation is also one of the starting points of the fdu scheme described in Sect. 6.

21 In ireg, $C^{\alpha \beta }$ is directly computed by subtracting its UV divergent part.

22 The value of the ‘extra integral’ *EI* is the same as the one of $(2\pi )^4 I_{3}^{{d}}[\mu ^2]$ obtained in fdf; see Eq. (3.36).

23 This result is identical to the one obtained in ireg, compare with Eq. (4.18).

24 This corresponds to the usual matrix element squared for massless particles computed in four dimensions, as given in Eq. (2.23).

25 Similar integrals have to be evaluated when using the ireg framework to determine the real contributions; see Eqs. (4.31). Their counterparts in ds are given in Eqs. (2.26).

26 This result is identical to the one obtained in ireg, compare with Eq. (4.32).

27 In other words, we could be working in any of the ds schemes mentioned in this article, with the only requirement that the associated manifold is Lorentzian, i.e. that it only contains *time* component and an arbitrary number of *spatial* ones.

28 This assumption is true whenever the incoming particles have fixed momentum, thus leading to a global constraint on the energy available for generating final-state radiation.

29 The result of the field renormalization coincides with the one of cdr and not with the one of fdh. In the latter scheme, the constant ‘$-4$’ would be replaced by ‘$-5$’; see e.g. Eq. (2.24) of Ref. [28].
